# A Radiolabeled Photoswitchable G Protein-Coupled Receptor Antagonist Enlightens Ligand Binding Kinetics Associated with Photoswitching

**DOI:** 10.1021/jacs.5c07349

**Published:** 2025-06-26

**Authors:** Lars C. P. Binkhorst, Ivana Josimovic, Bas De Boer, Icaro A. Simon, Frank Van Der Aa, Barbara A. Zarzycka, Iwan J. P. De Esch, Henry F. Vischer, Albert D. Windhorst, Maikel Wijtmans, Rob Leurs

**Affiliations:** † Division of Medicinal Chemistry, Amsterdam Institute of Molecular and Life Sciences, Faculty of Science, Vrije Universiteit Amsterdam, De Boelelaan 1108, 1081 HZ Amsterdam, The Netherlands; ‡ Department of Radiology and Nuclear Medicine, Amsterdam UMC, Location Vrije Universiteit, De Boelelaan 1117, 1081 HV Amsterdam, The Netherlands

## Abstract

Photopharmacology offers powerful opportunities to control protein signaling using photoresponsive ligands. Despite the vast potential of photoswitchable ligands for spatiotemporal target protein control, research on ligand-protein binding kinetics of these ligands remains limited. Herein, we describe the discovery of the first radiolabeled photoswitchable ligand, [^3^H]­VUF26063 ([^3^H]**3f**), to assess light-dependent ligand-protein binding kinetics in real time. The key compound (**3f**) is an arylazopyrazole-based antagonist targeting a prototypic family A G protein-coupled receptor (GPCR), the histamine H_3_ receptor (H_3_R), and enabled convenient radiolabeling via a growth vector on the pyrazole. Its photochemical properties, subnanomolar affinity of the *trans* isomer and a 50-fold decrease in affinity upon switching, allowed for reversible photochemical control of H_3_R binding kinetics in real time. The kinetic binding data obtained with this radiolabeled ligand indicate that **3f** isomerizes in the H_3_R extended binding pocket upon illumination. Our results shed light on the binding kinetics of photoswitchable ligands and will have relevance beyond GPCRs as targets.

## Graphical abstract

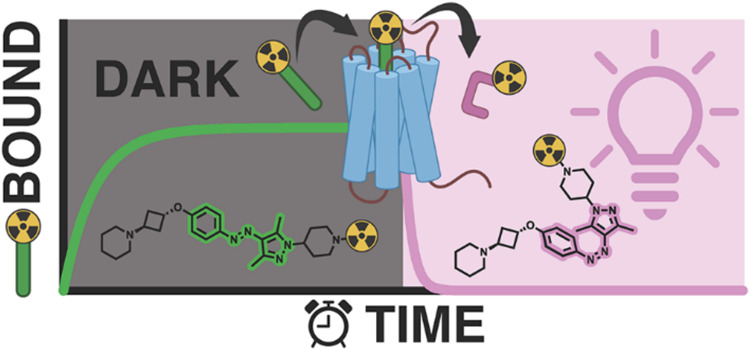

## Introduction

Photopharmacology offers a powerful approach to regulate protein function with high spatial and temporal precision through the use of photoresponsive ligands (e.g., photocaging and photoswitching).^1–3^ A variety of photoswitchable small-molecule ligands have been developed for various signaling proteins or enzymes, and are designed to reversibly isomerize between two distinct photostationary states (PSS) upon illumination, each capable of differentially modulating biology.^4,5^ While equilibrium properties such as PSS composition and target affinity have been the primary focus of most studies with photoswitchable ligands, kinetic parameters and particularly ligand-protein binding kinetics have received comparatively little attention.^6–8^

In this study, we addressed this gap using the histamine H_3_ receptor (H_3_R), an archetypal class A G protein-coupled receptor (GPCR), as a model system. The photopharmacology of GPCRs, one of the most extensively studied protein families and the target of approximately 36% of approved therapeutics,^9^ has seen remarkable advances in recent years and therefore these targets constitute an excellent model protein family.^10–13^ The H_3_R is predominantly expressed in the central nervous system, where it modulates the release of histamine and other key neurotransmitters.^14^ It is implicated in various neuropathologies, including Parkinson’s and Alzheimer’s diseases, neuropsychiatric conditions such as Tourette’s syndrome and schizophrenia, and sleep disorders like narcolepsy.^15–17^ Its therapeutic importance is evident from the approval of inverse agonist pitolisant for the treatment of narcolepsy.^18–20^

We have previously developed first-generation azobenzene-based H_3_R antagonists (e.g., VUF14862)^21^ and agonists (e.g., VUF15000).^22^ Indeed, our 2021 in-depth analysis of the chemical diversity of photoswitchable ligands for class A GPCRs showed that azobenzenes were predominantly used as photoswitchable moieties (84%).^12^ Yet, azobenzenes exhibit well-described drawbacks, such as low aqueous solubility, dependence on ultraviolet (UV) light for *trans*/*cis* isomerization, and suboptimal PSS_*trans*_ values. Hence, improved tools for advanced studies are eagerly awaited and the photopharmacology field at large has shifted toward next-generation photoswitchable moieties to improve PSS_*trans*_ values, increase solubility and enable photoisomerization at longer wavelengths for better tissue penetration and lower tissue damage.^23–25^ Among these, arylazopyrazoles have recently emerged as superior alternatives to azobenzenes, offering highly efficient and reversible switching at longer wavelengths, greater PSS percentages in both directions, and enhanced aqueous solubility owing to their heteroatom-rich structure.^5,26–29^

Using these recent advancements in the field, we developed an arylazopyrazole-based second-generation series of photoswitchable H_3_R ligands with improved physicochemical, photochemical, and pharmacological properties compared to our earlier H_3_R antagonist^22^ series. Key compound **3f** binds H_3_R with subnanomolar affinity, undergoes efficient and quantitative photoisomerization between *trans* and *cis* states and exhibits a 50-fold decrease in H_3_R affinity upon switching. Importantly, the pyrazole growth vector enabled straightforward radiolabeling of **3f**, yielding [^3^H]**3f** which is, to our knowledge, the first radiolabeled photoswitchable ligand. This unique and novel tool allowed us to probe the role of ligand-protein binding kinetics in light-mediated regulation of GPCR function and provided compelling evidence that photoisomerization can occur within the binding pocket of a target protein.

## Results and Discussion

### Design

Compound **1** ^21^ was chosen as a starting point (Figure 1 and Table 1). The generally accepted pharmacophore model for nonimidazole H_3_R antagonists entails a basic *N*,*N*-dialkylamino moiety separated by an aliphatic linker to an aromatic core decorated with additional substituents.^30,31^ Indeed, **1** comprises a piperidine bound to an unsubstituted azobenzene with a classical aminopropyloxy linker.^30–34^ To increase the affinity for H_3_R, the propyloxy side chain was rigidified through a *trans*-cyclobutyl group (**2**). Rigidification is a general strategy to improve target binding affinity^35^ and has proven to be successful in the H_3_R field.^21,36–39^ The azobenzene was subsequently replaced by a trimethylarylazopyrazole moiety (**3a**). In addition to its known benefits (*vide supra*), the pyrazole has the additional advantage of potentially improving the required molecular recognition event between ligand and protein, as heteroatoms are often involved in productive interactions with proteins, and presenting a synthetically tractable growth vector for appending additional protein recognition elements.^5^ We pursued growing from the nitrogen atom of the pyrazole with specific groups (**3b**–**f**), aiming to further improve solubility by increasing the dipole moment,^40^ probe additional molecular interactions within the H_3_R pocket,^41,42^ and potentially increase the affinity shift by inducing clashes between one of the photochemical isomers and the protein. To achieve this, relatively large and rigid substituents, i.e., six-membered rings, were chosen. A substituent without a hydrogen bond donor or acceptor in the six-membered ring (cyclohexyl analogue **3b**) and substituents with hydrogen bond donors and/or acceptors such as a tetrahydropyran, piperidine and *N*-methylpiperidine moiety (**3c**,**e**,**f** respectively) were introduced. The secondary amine in **3e** also provided a vector that could be used for radiolabeling. The designed compounds were prepared by 3 to 4 steps via routine synthetic sequences (Scheme S1 and Figure S1) using amongst others, a Boc-protected intermediate (**3d**) as key precursor.

**Figure 1:**
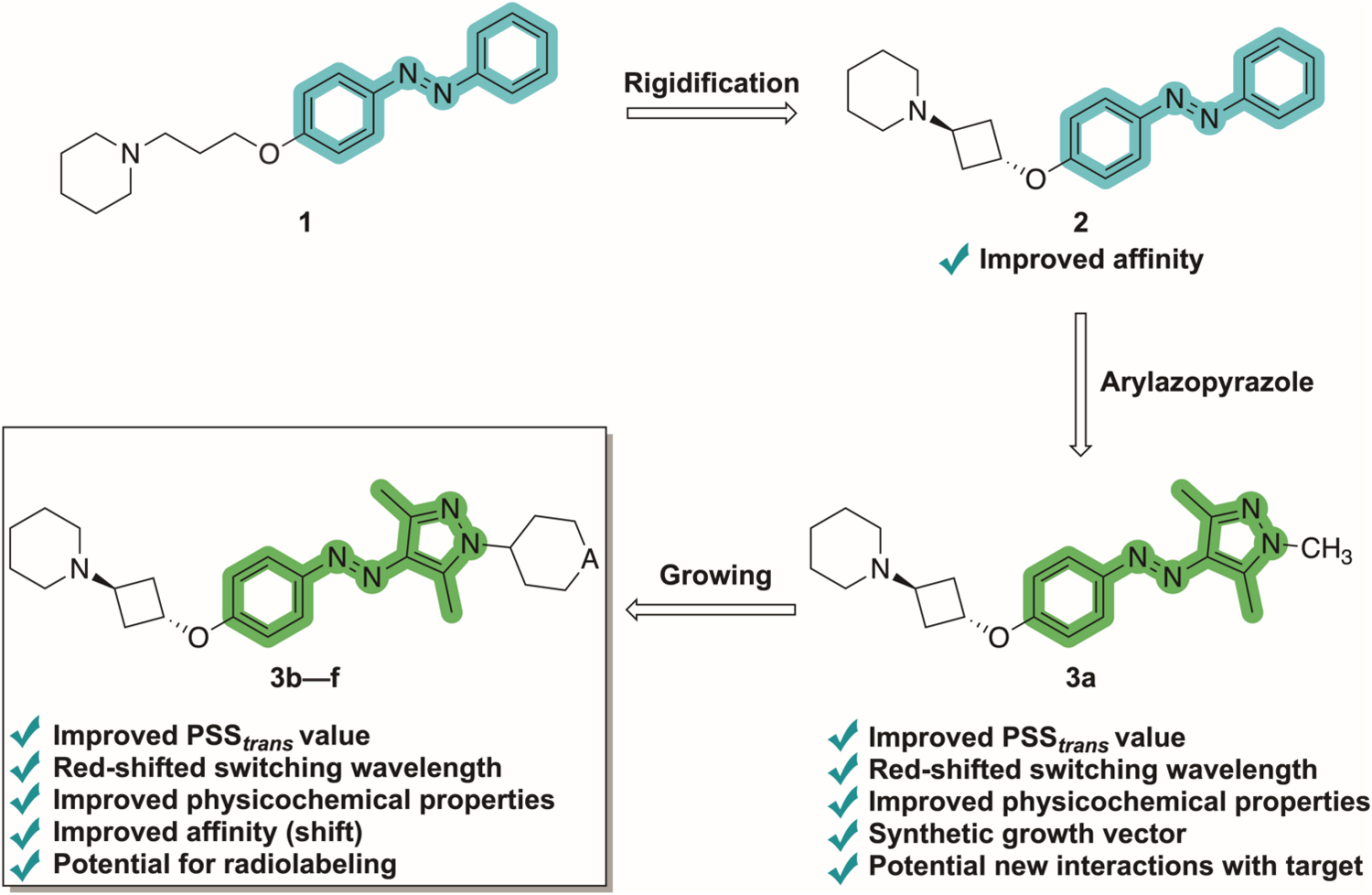
Design strategy toward high-affinity arylazopyrazole-based H_3_R antagonists. Improvements listed are relative to azobenzene ligand **1**.

**Table 1:**
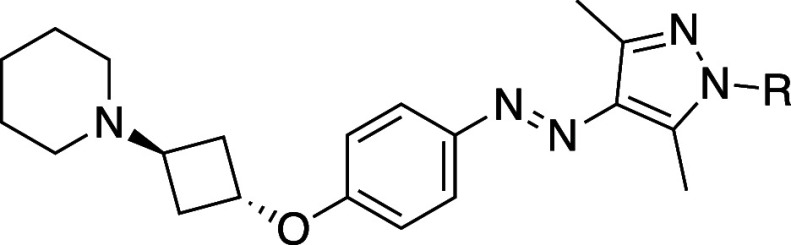

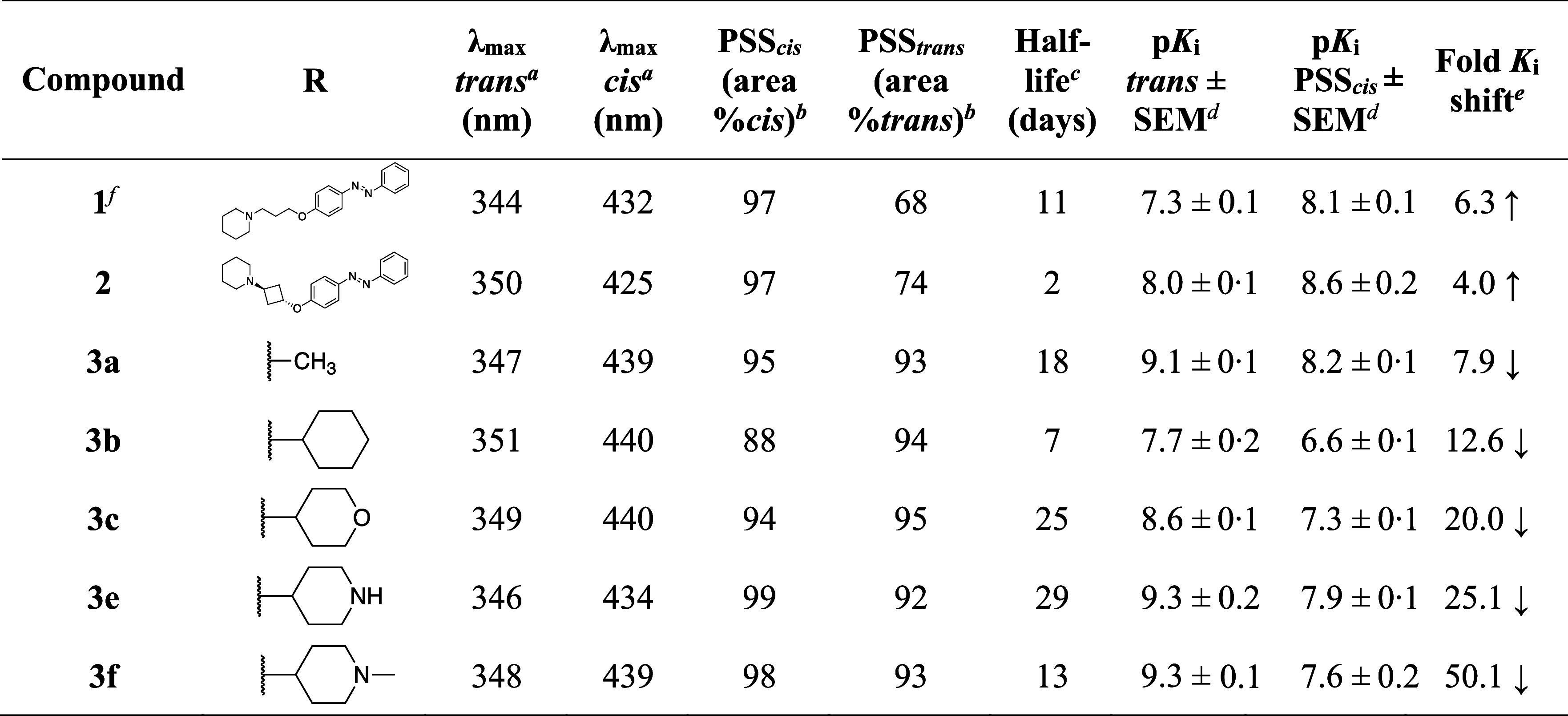
Photochemical Parameters, H_3_R Affinity (p*K*_i_) Values and Corresponding Affinity Shifts between PSS_*cis*_ and *trans* States

a Determined at 25 μM in HBSS buffer containing 1% DMSO.

b Photostationary state area percentages after illumination of *trans* isomer with 365 ± 10 nm at 1 mM in DMSO for 10 min as determined by LC-MS analysis at the isosbestic point, or illumination of PSS_*cis*_ states with 506 ± 20 nm (for **3**) or 434 ± 20 nm (for **1** and **2**) at 1 mM in DMSO for 10 min as determined by LCMS analysis at the isosbestic point.

c Approximate thermal relaxation half-life times of PSS_*cis*_ states in HBSS buffer + 1% DMSO, as estimated by the method of Ahmed et al.^46^ by extrapolating to 20 °C. Arrhenius plots are available in Figures S2–S7.

d Affinity (p*K*_i_) values as obtained from [^3^H]­NAMH competition experiments on cell homogenates overexpressing H_3_R. Values are means of *n* = 3 experiments, performed in triplicate ± SEM.

e Fold shift calculated as (high-affinity state)/(low-affinity state), with the arrow indicating lower (↓) or higher (↑) affinity of the PSS_*cis*_ state in comparison to the *trans* state.

f Data reported by Hauwert et al.^21^

### Structure–Photochemistry and Structure–Activity Relationships

Photochemical properties and target binding affinities were monitored in parallel to guide the design and synthesis cycles (Table 1). As expected, rigidification of the side chain of **1** improves target interaction, with **2** having nanomolar affinity and a 4-fold difference in affinity between *trans* **-2** and **2** at PSS_*cis*_ (“*cis*-on”). Replacing the azobenzene in **2** with a trimethylarylazopyrazole (**3a**) results in a more than 10-fold improved *K*_i_ value for *trans*-**3a** compared to *trans-* **2**. Since **3a** at PSS_*cis*_ shows reduced affinity compared to **2** at PSS_*cis*_, **3a** has an improved photochemically induced affinity shift but, interestingly, with a *trans*-on profile. Next to subnanomolar H_3_R affinity, **3a** also shows improved (photo)­chemical properties compared to **2** and these were, as expected, also observed for its analogues **3b**,**c**,**e**,**f**. First, as reported for many arylazopyrazole-based photoswitchable ligands,^26,27^ the *trans*-arylazopyrazole moiety in **3a**–**c**,**e**,**f** can be efficiently isomerized upon illumination (PSS_*cis*_ = 88–99%). Second, the estimated thermal half-life of PSS_*cis*_ states of **3a**–**c**,**e**,**f** at 20 °C in HBSS buffer (1% DMSO) is increased from 2 days for **2** up to at least a week (Figure S2–S7, and Table 1). Third, the enhanced band separation of the arylazopyrazoles^26,27^ allows the use of 506 nm (green light) for **3b**,**c**,**e**,**f** instead of 434 nm (blue light) required for **2**, to more efficiently (92–95 versus 74% for **2**) switch the arylazopyrazole-based ligands back from the PSS_*cis*_ state to the PSS_*trans*_ state. This observation is in line with previously reported differences in the efficiency of back-switching of azobenzene- and arylazopyrazole-based photoswitchable ligands.^27^

While, as expected, the photochemical properties of **3a**–**c**,**e**,**f** are not greatly affected by substitution on the pyrazole, the molecular interaction with the H_3_R binding pocket is sensitive to *N*-substitution of the pyrazole (Figure S8 and Table 1). Introducing a lipophilic cyclohexyl ring (**3b**) results in a 30-fold decrease in H_3_R affinity compared to **3a** (Figure S8A,B). Interestingly, incorporating an oxygen or nitrogen atom in the cyclohexyl ring (**3c**,**e**,**f**) restores high-affinity target binding in combination with an improved light-induced affinity shift of 20–50 fold (Figure S8C,D and Table 1). As mentioned, (substituted) arylazopyrazole moieties potentially also improve solubility compared to azobenzene-based ligands. Therefore, nephelometry^43,44^ was used to qualitatively compare the solubility of arylazopyrazole **3f** to our first-generation azobenzene-based antagonist VUF14862 ^21^ (Figure S9). Neither of these compounds form aggregates^45^ nor precipitate at concentrations of relevance in our pharmacology assays (vide infra). However, *trans*-VUF14862 shows a relatively high tendency to aggregate/precipitate at high concentrations (>10^–4.5^ M), while this was not observed for **3f** in either state, suggesting an improved solubility profile of **3f**. Ligand **3f** was selected for in-depth studies given its excellent switching properties (PSS_*cis*_ = 98%, PSS_*trans*_ = 93%), improved solubility, subnanomolar H_3_R affinity (p*K*_i_ = 9.3 ± 0.1) of the *trans* isomer, and the 50-fold difference in affinity between *trans* and PSS_*cis*_ states. Its clean and robust photoswitching was further confirmed by liquid chromatography–mass spectrometry (LC–MS), NMR and UV studies (Figures S10–S12).

### Binding Mode of **3f**

Molecular modeling studies were performed to rationalize the observed affinities of the different isomers of **3f**. Both isomers were docked in the crystal structure of the human H_3_R in complex with antagonist PF03654746 (PDB: 7F61).^41^ Four independent 1 μs all-atom molecular dynamics (MD) simulations were performed for each complex, to validate predicted binding poses. The predicted pose of *trans*-**3f** (Figure 2A,B) is located within the orthosteric pocket with its aromatic photoswitchable moiety extending into the extended binding pocket (EBP) formed by TM2, TM7 and ECL2. This region forms a tight aromatic cluster surrounded by residues Y91^2.61^, Y94^2.64^, W110^3.28^, Y189^ECL2^, F193^ECL2^, and F398^7.39^. Notably, the predicted binding pose of *trans* **-3f** indicates a substantial overlap with the pose of PF03654746 in the H_3_R X-ray structure (Figure S13). In contrast, the folded conformation of the *cis* isomer does not fit within the EBP and needs an enlarged binding pocket to be accommodated, where the arylazopyrazole moiety is located toward TM5 and 6 moving away from the EBP and its key aromatic cluster residues Y91^2.61^, Y94^2.64^, W110^3.28^ (Figure 2C,D). Thus, this binding pose does not overlap well with the crystallographic binding mode of PF03654746 (Figure S13). Both *trans*- and *cis*-**3f** form an ionic interaction with D114^3.32^, that anchors the piperidine in a similar position, deep inside the orthosteric binding pocket (Figure 2B,D, respectively). In the MD trajectories, the more extended, linear shape of *trans*-**3f** reaches the EBP, which results in a binding pose making increased contact with the extracellular loop region, with the *N*-methylpiperidine group being solvent exposed (Figure 2A,B). This solvent exposure can explain the structure–activity relationship (SAR) listed in Table 1 for the arylazopyrazole-based *trans* isomers. While the small *N*-methyl group (**3a**) likely experiences minimal desolvation costs near the entrance of the binding pocket, the larger and apolar cyclohexyl group (**3b**) will experience a greater desolvation penalty. In contrast, more polar substituents (**3c**,**e**,**f**) are better accommodated in a solvent-exposed environment and can maintain favorable interaction with surrounding water molecules. *Trans*-**3f** also engages in aromatic interactions with Y189^ECL2^, F193^ECL2^, F398^7.39^, an additional salt bridge with D391^7.32^ and a transient H-bond interaction with Y394^7.35^ (Figures 2B and S14A). While the folded conformation of *cis*-**3f** (Figure 2C) also engages in aromatic interactions with F193^ECL2^, it further forms a secondary salt bridge with E395^7.36^ and occasional cation-π interactions with R381^6.58^ and F398^7.39^ (Figures 2D and S14B). The conformational differences between the isomers also induce a 90-degree rotation of the cyclobutyl linker. For *trans*-**3f**, the oxygen atom in the linker is directed toward Y115^3.33^, while for *cis*-**3f**, the oxygen atom is oriented toward W110^3.28^, contributing to distinct spatial arrangements of the isomers within the H_3_R binding site (Figure 2B,D). In all, the linear shape of *trans*-**3f** demonstrates an improved fit in the H_3_R binding pocket compared to *cis*-**3f**, potentially explaining the observed higher binding affinity of *trans*-**3f** compared to **3f** at PSS_*cis*_.

**Figure 2:**
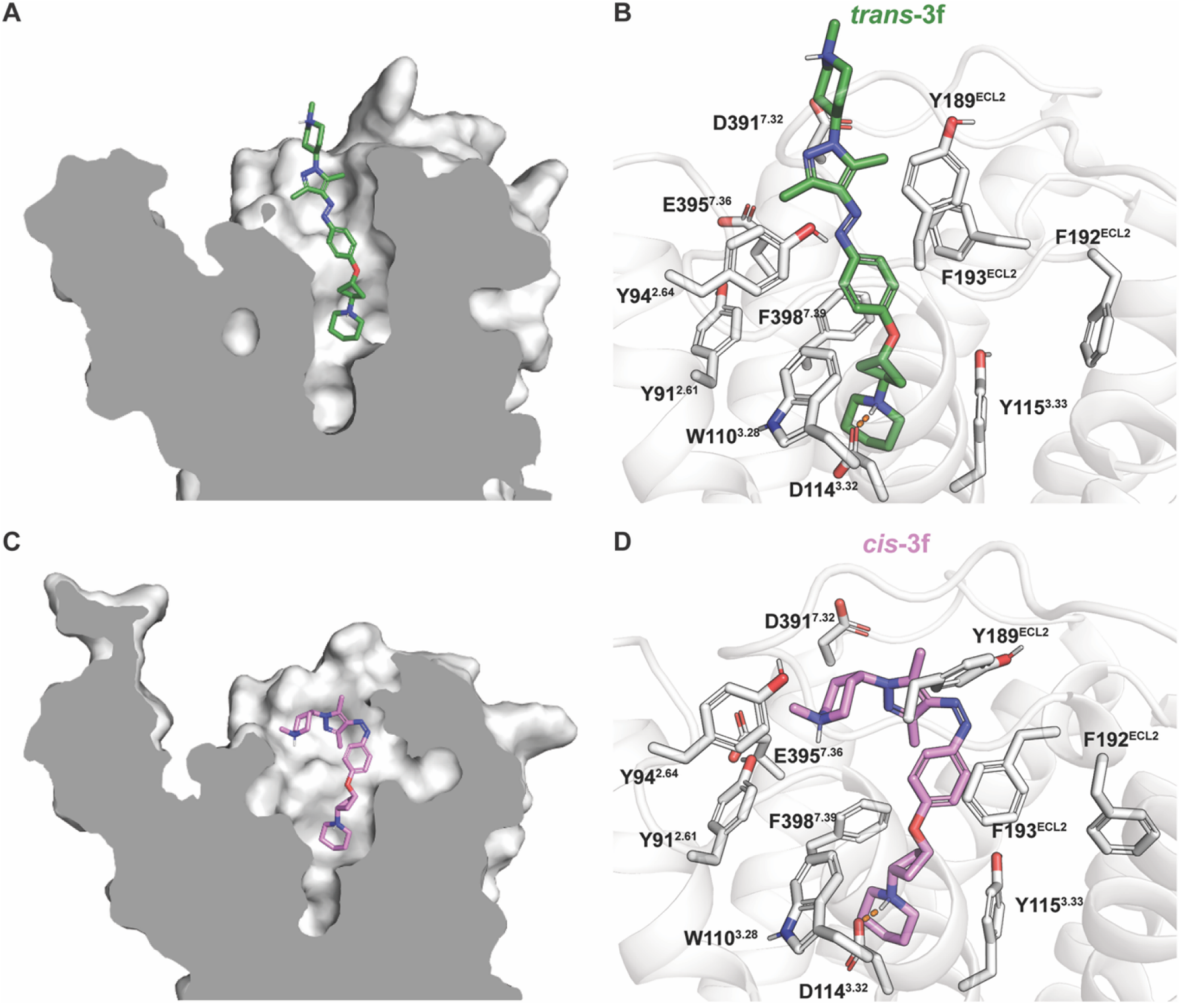
(A) Slice-through depiction of the H_3_R pocket formed by *trans*-**3f**. (B) Predicted binding pose of *trans*-**3f** (green carbon atoms). (C) Slice-through depiction of the H_3_R pocket formed by *cis*-**3f**. (D) Predicted binding pose of *cis*-**3f** (pink carbon atoms). Poses are based on representative MD snapshots. Ionic interactions are shown in orange dashed lines in panel B and D.

### Photopharmacological Evaluation of **3f**

Arylazopyrazole **3f** is a H_3_R ligand with subnanomolar affinity and excellent selectivity over the other three histamine receptor proteins (Table 1, Figures 3A and S15). Competition binding experiments reveal that *trans-* **3f** does not bind to any of the other three histamine receptor subtypes at concentrations lower than 1 μM (Figure S15). Moreover, *trans-* **3f** binds with high affinity to the mouse H_3_R (p*K*_i_ = 8.8 ± 0.2) and maintains a 42-fold shift in affinity upon photoisomerization to **3f**-PSS_*cis*_ (Figure S16).

**Figure 3:**
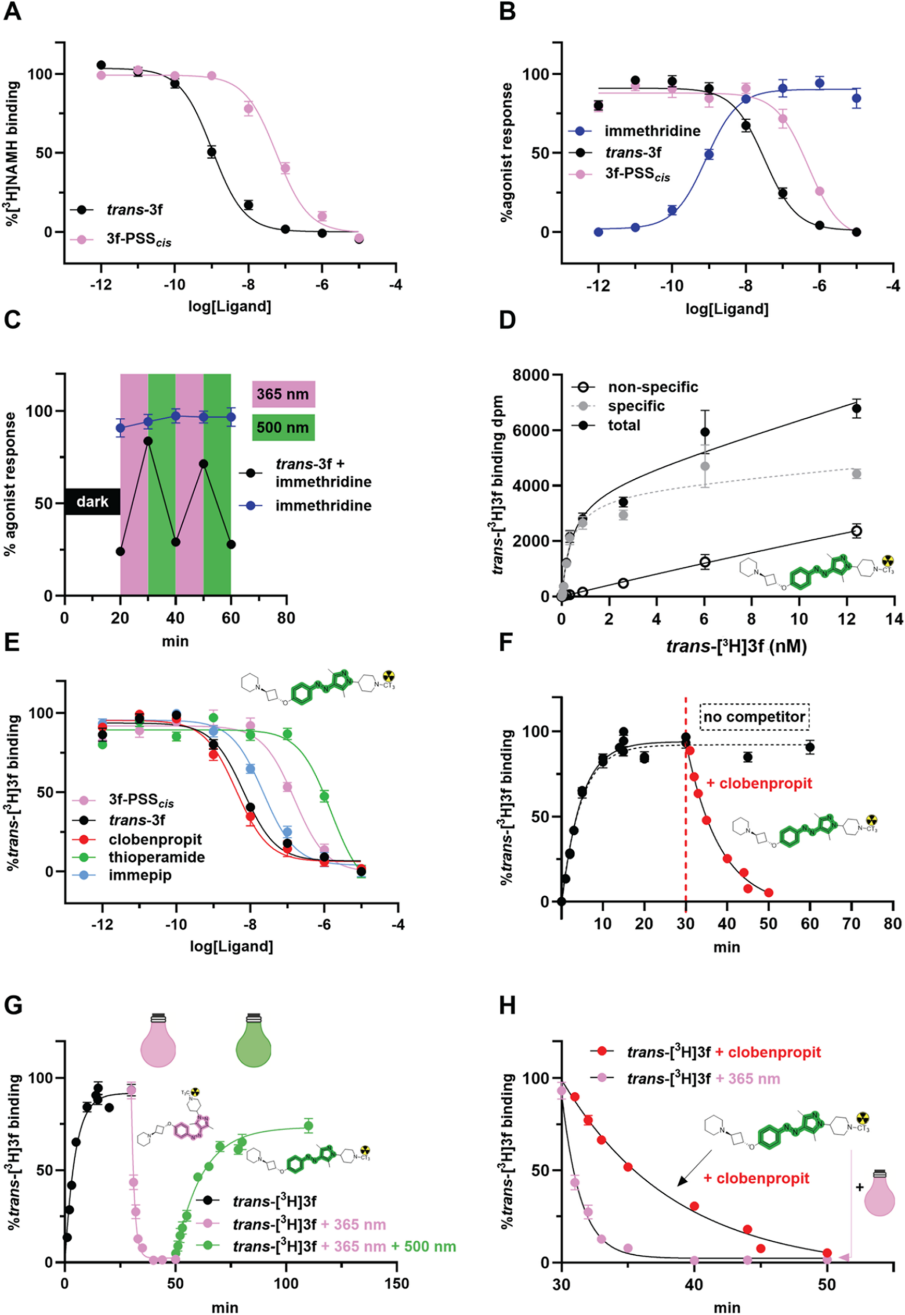
Photopharmacological characterization of **3f** and its radiolabeled analogue [^3^H]**3f**. (A) Concentration–response curves of **3f** obtained in radioligand H_3_R binding assays in competition with 1.6 nM [^3^H]­NAMH. (B) Concentration–response curves of immethridine alone and 10 nM immethridine in competition with *trans-* **3f** or **3f**-PPS_*cis*_ in a functional H_3_R-NanoBit-PKA assay after 15 min of incubation. (C) Dynamic alterations in functional H_3_R-dependent nBit-cAMP sensor activity, induced by competition of 10 nM immethridine with 100 nM *trans*-**3f** and subsequent switching thereof, in comparison with immethridine activity over switching cycles. Dark indicates the preincubation time of *trans*-**3f** before immethridine stimulation and subsequent switching, magenta stripes indicate irradiation with 365 nm light, and green stripes indicate irradiation with 500 nm light. (D) Saturation binding experiments with *trans*-[^3^H]**3f** indicative of total, specific and nonspecific binding on cell homogenates expressing H_3_R. (E) Displacement of *trans*-[^3^H]**3f** with H_3_R-specific unlabeled ligands in radioligand binding assays. (F) Association and dissociation binding curves of *trans*-[^3^H]**3f** (0.5 nM). A 60 min association was performed with no competitor present. Dissociation from H_3_R was initiated with 10 μM clobenpropit after 30 min of preincubation with *trans*-[^3^H]**3f**. (G) Light-dependent modulation of *trans*-[^3^H]**3f** (0.5 nM) H_3_R binding and kinetics without a competitor present. (H) Dissociation of *trans*-[^3^H]**3f** (0.5 nM) from H_3_R initiated either with 10 μM clobenpropit, or without competitor but using 365 nm light. Data represent mean ± SEM of at least *n* = 3 experiments, performed in triplicate. Curves are normalized to their own plateaus.

The ability of **3f** to modulate H_3_R signaling in living cells was evaluated. H_3_R is coupled to Gα_i_ proteins both in native tissues and transfected cells.^47,48^ The canonical inhibition of forskolin-stimulated adenylate cyclase activity following H_3_R activation with the high-affinity H_3_R-specific agonist immethridine^49^ (pEC_50_ = 9.1 ± 0.1, Figure 3B and Table S1) was measured in transiently transfected HEK293T cells coexpressing H_3_R and a NanoBiT-based cAMP sensor, measuring cAMP-dependent subunit disassembly of protein kinase A.^50^ Both *trans-* **3f** and 3f-PSS_*cis*_ (i.e., preirradiated *trans*-**3f**) show concentration-dependent antagonism of 10 nM immethridine-induced H_3_R activation (Figure 3B). Reminiscent of the pattern observed for **3f** in radioligand binding, *trans-* **3f** shows a significantly higher antagonistic potency (pIC_50_ = 7.5 ± 0.1) in this functional assay, compared to **3f**-PSS_*cis*_ (pIC_50_ = 6.4 ± 0.1). The affinity determined in radioligand binding experiments shows a larger shift between *trans-* **3f** and **3f**-PSS_*cis*_ (50-fold, Table 1) in comparison to the observed potency shift with the live cell cAMP sensor (12.6-fold, Table S1). This difference most likely reflects the difference in assay conditions (membranes versus live cells, different buffers). Also, bioluminescence stemming from light-based assays is known to potentially interfere with the isomeric ratio of photoswitchable ligands.^12,51,52^ With nanoluciferase emitting light with a peak around 460 nm^53^ and the possibility of some **3f**-PSS_*cis*_ back-switching to **3f**-PSS_*trans*_ between 430 and 500 nm (Figure S10A) during the time of data collection, the observed potency shift between *trans-* **3f** and **3f**-PSS_*cis*_ might be underestimated.

Ligand **3f** was investigated in an in situ dynamic photopharmacology assay using transiently transfected HEK293T cells, expressing H_3_R and the cAMP sensor (Figure S17A). Preincubation of 100 nM of *trans-* **3f** with 10 nM immethridine blocked the H_3_R agonist response for approximately 80% (Figure 3C). Subsequent illumination with 365 nm LED light (10 min, 2 mW, Figure S17B) results in the reversal of the response up to approximately 80% of the maximal agonist response, indicative of *in situ* switching of *trans*-**3f** to **3f**-PSS_*cis*_ and a concomitant loss of H_3_R blockade. In situ back-switching with 500 nm LED light (10 min, 1.5 mW, Figure S17B) restored H_3_R blockade, confirming successful **3f**-PSS_*cis*_ to **3f**-PSS_*trans*_ conversion. An additional 365–500 nm illumination cycle results in a similar modulation of the H_3_R activity (Figures 3C, S17B and Table S1).

### Synthesis and Application of [^3^H]**3f**

The kinetics of the molecular recognition events underlying photoswitchable ligands remain poorly understood.^6–8^ Therefore, we used the desmethyl analogue **3e** to prepare radiolabeled **3f** (i.e., [^3^H]**3f**) as a means to directly study H_3_R-ligand binding kinetics. Specifically, the availability of **3e** allowed the use of a previously described CT_3_ incorporation using CT_3_-nosylate^54,55^ (Scheme 1) to prepare [^3^H]**3f**. To our knowledge, *trans*-[^3^H]**3f** is the first radiolabeled photoswitchable ligand ever reported. Purity and light-dependent switching of the radiolabeled compound were confirmed by liquid chromatography and the recording of [^3^H]-chromatograms using a β-scintillation HPLC detector. *trans*-[^3^H]**3f** contained 2% *cis* isomer, while after 10 min of irradiation with 365 nm LED light (10 V, 2 mW), 96% *cis* isomer was obtained (Figures S18 and S19), yielding similarly high PSS_*cis*_ values as observed for nonlabeled **3f** (Table 1).

**Scheme 1:**
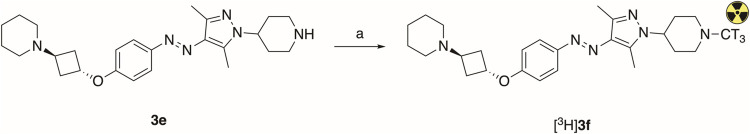
Synthesis of [^3^H]**3f** ^a^

a Reagents and conditions used: (a) [^3^H]­methyl nosylate, MeCN, 70 °C, 15 min.

In saturation binding studies, *trans*-[^3^H]**3f** binds H_3_R with subnanomolar affinity (p*K*_d_ = 9.2 ± 0.0, Figure 3D and Table 2) and exhibits low nonspecific binding. The binding of *trans*-[^3^H]**3f** to H_3_R is effectively abrogated by the reference H_3_R ligands clobenpropit, thioperamide and immepip (Figure 3E and Table S2). Both unlabeled **3f** isomers also fully abrogate *trans*-[^3^H]**3f** binding to H_3_R, with *trans*-**3f** (p*K*_i_ = 9.0 ± 0.1) being considerably more effective than **3f**-PSS_*cis*_ (p*K*_i_ = 7.7 ± 0.0, Figure 3E and Table S2). In kinetic experiments, the binding of 0.5 nM *trans*-[^3^H]**3f** to H_3_R reaches equilibrium at 15 min (Figure 3F). Bound *trans*-[^3^H]**3f** dissociates from H_3_R with a dissociation half-life (*t*_1/2_) of 4.9 ± 0.4 min upon addition of an excess of clobenpropit (10 μM). This results in *k*_off_ = 0.15 ± 0.02 min^–1^ and *k*_on_ = 129 ± 28 × 10^6^ M^–1^ min^–1^ for *trans*-[^3^H]**3f**. Compared to *trans*-[^3^H]**3f**, kinetic H_3_R binding experiments with [^3^H]**3f**-PSS_*cis*_ (obtained by preilluminating *trans*-[^3^H]**3f**) revealed a 10-fold lower *k*_on_ value (13 ± 6 × 10^6^ M^–1^ min^–1^) but a similar *k*_off_ (0.11 ± 0.00 min^–1^) value (Table 2 and Figure S20A). The calculated kinetic affinities for *trans*-[^3^H]**3f** and [^3^H]**3f**-PSS_*cis*_ (p*K*_d*k*_in__ = *k*_off_/*k*_on_ = 8.9 ± 0.2 and 7.9 ± 0.3, respectively) correspond well to the affinities obtained in equilibrium saturation and competition binding assays (p*K*_d_ = 9.2 ± 0.0 and p*K*_i_ = 7.6 ± 0.2, respectively; Table 2).

**Table 2:** Kinetic Parameters of [^3^H]**3f** Binding to H_3_R as Determined in Association and Dissociation Radioligand Binding Experiments, Calculated Kinetic Affinities and Affinities Obtained from Binding Experiments

**state**	***k*_on_** (10^6^ M^–1^ min^–1^)	***k*_off_** (min^–1^)	***t*_1/2_** (min)^a^	**p*K*_d(*k*_in_)_** ^b^	**p*K*_d_ or p*K*_i_**
*trans*	129 ± 28	0.15 ± 0.02 (clobenpropit)	4.9 ± 0.4	8.9 ± 0.2	9.2 ± 0.0^c^
		1.12 ± 0.28 (365 nm light)	0.8 ± 0.2		
PSS_*cis*_	13 ± 6	0.11 ± 0.00 (clobenpropit)	6.2 ± 0.2	7.9 ± 0.3	7.6 ± 0.2^d^

a Data are mean ± SEM of at least *n* = 3 independent experiments. Dissociation *t*_1/2_ = ln 2/*k*_off_.

b p*K*_d(*k*_in_)_ = *k*_off_/*k*_on_.

c p*K*_d_ obtained in saturation binding experiments.

d p*K*_i_ of unlabeled PSS_*cis.*_

Next, we probed if H_3_R binding of *trans*-[^3^H]**3f** could be modulated by *in situ* photoswitching induced by illumination (Scheme S2 and Figure 3G). Exposure of prebound *trans*-[^3^H]**3f** to 365 nm light resulted in a rapid dissociation from H_3_R to approximately 0% (Figure 3G, magenta), even though no competitor is present. This is consistent with fast quantitative photoswitching of *trans*-[^3^H]**3f** to [^3^H]**3f**-PSS_*cis*_, which will not bind H_3_R at this concentration due to its lower affinity. Subsequent exposure to 500 nm green light re-established H_3_R binding of *trans*-[^3^H]**3f** over time (Figure 3G, green), confirming successful back-switching of the low-affinity [^3^H]**3f**-PSS_*cis*_ state to high-affinity *trans*-[^3^H]**3f**. Comparing the kinetics of clobenpropit-induced dissociation of *trans*-[^3^H]**3f** and [^3^H]**3f**-PSS_*cis*_ (Figure S20A) as well as the 365 nm light-induced dissociation of *trans*-[^3^H]**3f** reveals a remarkable difference in dissociation kinetics (Figures 3H, S20B and Table 2). Photoswitching of prebound *trans*-[^3^H]**3f** by 365 nm illumination results in 6.2-fold faster dissociation kinetics (*t*_1/2_ = 0.8 ± 0.2 min) as compared to the dissociation of dark *trans*-[^3^H]**3f** (*t*_1/2_ = 4.9 ± 0.4 min**)** and [^3^H]**3f**-PSS_*cis*_ (*t*_1/2_ = 6.2 ± 0.4 min) (Table 2). Similar findings concerning light-dependent dissociation rates of photoswitchable negative allosteric modulators of the Class C GPCR mGlu_5_ have been reported using a mass spectrometry binding assay.^6^ Our kinetic data exclude the dissociation process from being governed by photoswitching of unbound *trans*-[^3^H]**3f** in solution and subsequent readjustment of the binding equilibrium.

This photochemically induced kinetic pattern is reminiscent of the known effects of retinal confinement within a photoresponsive protein.^56–59^ Retinal is a naturally occurring photoswitchable chromophore that is covalently linked to opsin proteins via a Schiff’s base to a lysine residue in TM7. Upon illumination retinal is switched from its 11-*cis*-retinal configuration to an all-*trans*-retinal form. This configurational change of the chromophore subsequently results in GPCR protein activation and triggers a cascade of biochemical events that convert light into electrical signals for vision.^60^ Retinal isomerization occurs more rapidly and with greater stereoselectivity within the binding pocket of the protein host, achieving a higher quantum yield compared to when it occurs in solution.^57,58^ However, for synthetic photoswitchable compounds, including azobenzene-based ones, confinement can affect their photochemical behavior less advantegously.^59,61^ As suggested for azobenzene-based ligands for an allosteric site of mGluR_5_,^6^ our kinetic data provide evidence that **3f** is switching when bound to H_3_R. Specifically, our modeling studies (Figure 2) suggest that the photoswitchable moiety of **3f** sits within the EBP surrounded by flexible extracellular loops, indicating sufficient conformational flexibility to allow for this switching to occur, although the constraints of the interactions of **3f** within the protein likely still reduce the efficiency of **3f** to undergo a *trans*-*cis* isomerization compared to in solution. For an azobenzene-based A_2A_ receptor ligand, it has been proposed based on detailed spectroscopy studies that the overall outcome (efficiency and rate) of switching in the protein pocket depends on the isomerization of the azo moiety, reorientation of the phenyl group, and the potential for longer-lived excited states due to protein-coupled motion.^7^ It was also recently shown, using time-resolved crystallography, that related photoswitchable A_2A_ receptor ligands can be switched in the protein binding pocket and that intermediate states of ligand dissociation can be resolved.^62^ In this system, confinement of photoswitchable ligands by protein binding reduces the quantum efficiency of photoisomerization.^62^ Although we focused on binding kinetics and not on photoswitching kinetics, it is likely that the factors uncovered in the A_2A_ studies will also be involved in our case and will collectively manifest themselves in different binding kinetics. Indeed, the same A_2A_ study^62^ highlighted that photoswitching of a bound ligand can lead to conformational changes in the protein and, in line with this finding, we hypothesize that photoswitching of **3f** in the protein binding pocket provides a metastable *cis*-**3f/**H_3_R complex from which *cis*-**3f** is expelled faster than preformed *cis*-**3f** is expelled in the dark. As such, our data contribute to the emerging conceptual framework on the effect of protein confinement on photoswitchable ligand behavior, that is needed for the future design of optimal photoswitchable ligands.

## Conclusions

We have designed and synthesized a second-generation photoswitchable antagonist series for an archetypical GPCR (H_3_R), incorporating the arylazopyrazole as a photoswitchable moiety. Classical limitations of azobenzene-based photoswitchable ligands were efficiently addressed with the new series enhancing aqueous solubility, enabling the use of green light to reach PSS_*trans*_, and boosting high PSS values both ways. Pharmacological parameters such as the overall affinity and the affinity shift were improved from template **1** to key compound VUF26063 (**3f**). Ligand **3f** exhibits a 50-fold affinity shift between its isomers and is a high-affinity, subnanomolar *trans*-on antagonist for H_3_R that can reversibly modulate H_3_R-dependent signaling in living cells in a light-dependent manner. Capitalizing on a readily accessible synthetic growth vector in **3f**, we prepared the first radiolabeled photoswitchable ligand [^3^H]­VUF26063 ([^3^H]**3f**). This compound was used to characterize the kinetic aspects of receptor–ligand binding events underlying the dynamic modulation of receptor signaling and these studies indicated that the *trans* isomer undergoes light-induced isomerization in the H_3_R EBP. Our study highlights the potential of detailed kinetic characterization of the binding process for photoswitchable ligands and could aid in a better prediction of the spatiotemporal effects of protein modulation by photoswitchable small molecules.

## Supplementary Material



## Notes

Synthesis of **3a-f**, detailed photochemical characterization of **3f**, additional modeling data on **3f**, detailed experimental procedures and methods, photopharmacological characterization and chemical characterization for the compounds (PDF)

## ∥

Department of Drug Design and Pharmacology, Faculty of Health and Medical Sciences, University of Copenhagen, Jagtvej 160, 2100 Copenhagen, Denmark

## Abbreviations

1D NOESY: one-dimensional nuclear Overhauser enhancement spectroscopy
AC: adenylyl cyclase
BRET: bioluminescence resonance energy transfer
cAMP: cyclic adenosine mono phosphate
DMF: dimethylformamide
EBP: extended binding pocket
FBS: fetal bovine serum
H_1_R: histamine H_1_ receptor
H_2_R: histamine H_2_ receptor
H_3_R: histamine H_3_ receptor
H_4_R: histamine H_4_ receptor
HA: hemagglutinin protein tag
HBSS: Hank’s balanced saline solution
LgBit: large Bit
MD: molecular dynamics
NAMH: N^α^-methylhistamine
nBit: nanoBit
nGlo: furimazine
nLuc: nanoluciferase enzyme
PBS: phosphate-buffered saline
PEI: polyethyleneimine
PSS: photostationary state
SAR: structure–activity relationship
SmBit: small Bit
