# Supplementary material for: A Radiolabeled Photoswitchable G Protein-Coupled Receptor Antagonist Enlightens Ligand Binding Kinetics Associated with Photoswitching
Source: J Am Chem Soc. 2025 Jun 26;147(27):23991–4000. doi: 10.1021/jacs.5c07349 (PMC12257542; doi:10.1021/jacs.5c07349)
Supplement: Supplementary file 1 [file ja5c07349_si_001.pdf]

Supporting Information

A radiolabeled photoswitchable G protein-coupled receptor antagonist enlightens ligand binding kinetics associated with photoswitching

Lars C.P. Binkhorst,[1,§] Ivana Josimovic,[1,§] Bas De Boer,[1] Icaro A. Simon,[1,#] Frank Van Der Aa,[2] Barbara A. Zarzycka,[1] Iwan J.P. De Esch,[1] Henry F. Vischer,[1] Albert D. Windhorst,[2] Maikel Wijtmans,[1,*] Rob Leurs[1,*]

*Corresponding authors: m.wijtmans@vu.nl, r.leurs@vu.nl
§L.C.P.B and I.J. contributed equally

[1]Division of Medicinal Chemistry, Amsterdam Institute of Molecular and Life Sciences, Faculty of Science, Vrije Universiteit Amsterdam, De Boelelaan 1108, 1081 HZ Amsterdam, The Netherlands

[2]Department of Radiology and Nuclear Medicine, Amsterdam UMC, location Vrije Universiteit, De Boelelaan 1117, 1081 HV Amsterdam, the Netherlands

[#]Present Address: Department of Drug Design and Pharmacology, Faculty of Health and Medical Sciences, University of Copenhagen, Jagtvej 160, 2100 Copenhagen, Denmark

Table of contents

Synthesis of arylazopyrazoles **3a-f** (Scheme S1)	S3
1D NOESY spectrum of **3f** (Fig. S1)	S4
Photochemical analysis of **2** and **3** (Fig. S2-S7)	S5-S10
Binding of **3a**,**b**,**c**,**e** to the human H_3 histamine receptor (Fig. S8)	S11
Nephelometry data of **3f** and VUF14862 (Fig. S9)	S11
Detailed photochemical characterization of **3f** (Fig. S10)	S12
LC chromatograms for switching of **3f** (Fig. S11)	S13
^{1}H-NMR switching of **3f** at different time points (Fig. S12)	S14
Overview of binding poses of **3f** and PF03654746 (Fig. S13)	S15
Ligand-protein interactions of **3f** (Fig. S14)	S16
Binding of **3f** to human H_1, H_2 and H_4 histamine receptors (Fig. S15)	S17
Binding of **3f** to mouse H_3R (Fig. S16)	S17
Overview of pharmacological parameters and associated switching conditions of **3f** in the functional NanoBit-PKA assay in HEK293T cells (Table S1)	S18
Functional dynamic light modulation of **3f**-mediated H_3R activity in HEK293T cells (Fig. S17)	S18
^{3}H chromatogram of [^{3}H]**3f** switching (Fig. S18)	S19
UV chromatogram of [^{3}H]**3f** switching (Fig. S19)	S20
Overview of affinity values of non-labeled H_3R ligands from competition binding experiments with *trans*-[^{3}H]**3f** or [^{3}H]-NAMH (Table S2)	S21
H_3R binding kinetics of [^{3}H]**3f**-PSS$_{cis}$ (Fig. S20)	S22
Scheme of dynamic binding experiments of [^{3}H]**3f** (Scheme S2)	S22
^{3}H and UV chromatogram of [^{3}H]**3f** (Fig. S21)	S23
Experimental procedures and data analysis – Photopharmacology	S24-S27
Experimental procedures – Computational chemistry	S28-S29
Experimental procedures – Photochemistry	S30
Experimental procedures – Synthesis	S31-S39
Chemical analyses	S40-S68
References	S69-S70

Scheme S1. General synthetic route for arylazopyrazoles **3a-f.**[a]

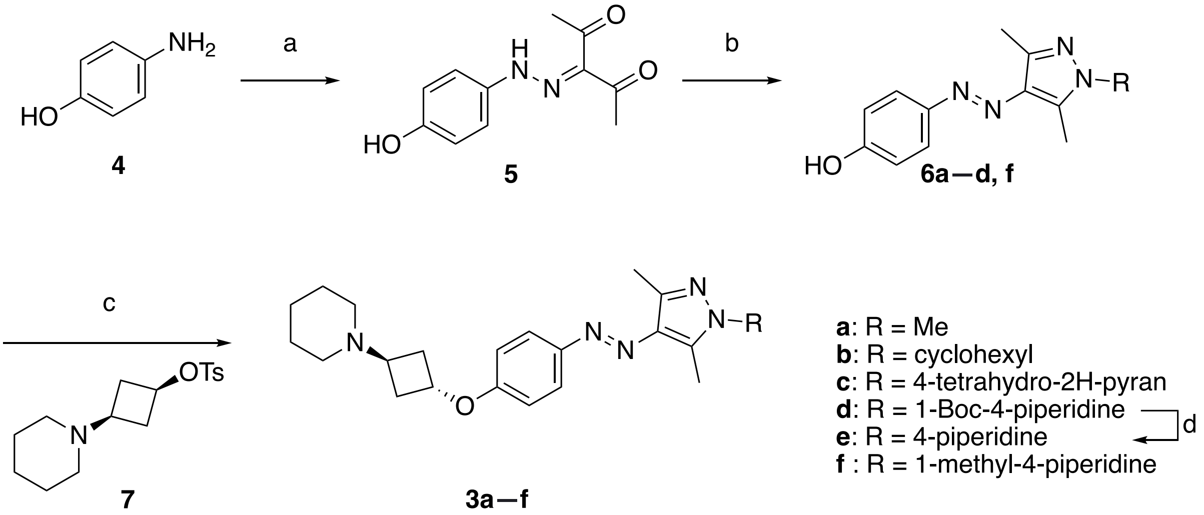

[a]Reagents and conditions used: (a) (i) $NaNO_2$, HCl, H_2O/AcOH, 0 °C, 30 min; (ii) acetylacetone, NaOAc, EtOH/H_2O, 0 °C, 30 min, 58%; (b) $RNHNH_2$, TEA, EtOH, reflux, 17 h, 57-96%; (c) (i) NaH, DMF, r.t., 30 min; (ii) **7**, 75 °C, DMF, 17 h, 24-52%; (d) HCl, dioxane, rt, 17 h, 70%;

For the synthesis of **3a-f**, 4-aminophenol (**4**) was converted by a diazonium coupling with acetylacetone to give **5** (Scheme 1).[1] This intermediate was heated under reflux with substituted hydrazines to give phenols **6a–d**,**f**. Subsequent coupling with *cis*-tosylate **7**[2] gave compounds **3a–d**,**f** with the expected *trans* configuration of the 3-piperidino-cyclobutoxy motif as confirmed with 1D NOESY (Figure S1). Compound **3d** was deprotected under acidic conditions to give **3e**.

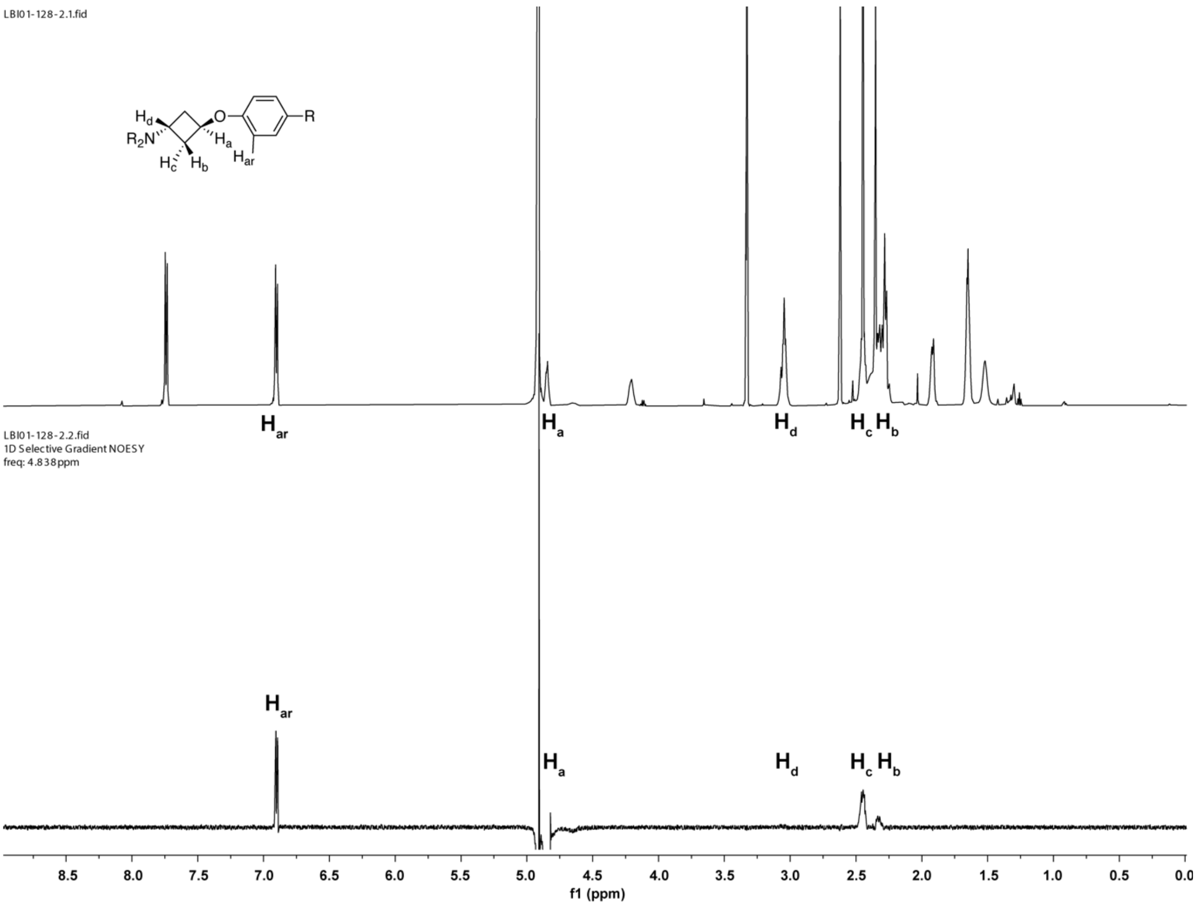

Figure S1. ^{1}H NMR spectrum (top panel) and 1D NOESY spectrum (bottom panel) of compound **3f** in CD_3OD measured on a Bruker Avance III HD 600 MHz spectrometer**.** Spectra are in line with the previously reported 2D NOE spectra of the *trans*-3-cyclobutoxy linker.[2,3] Proton H_a shows correlation with H_b and H_c, while no correlation between H_a and H_d was observed.

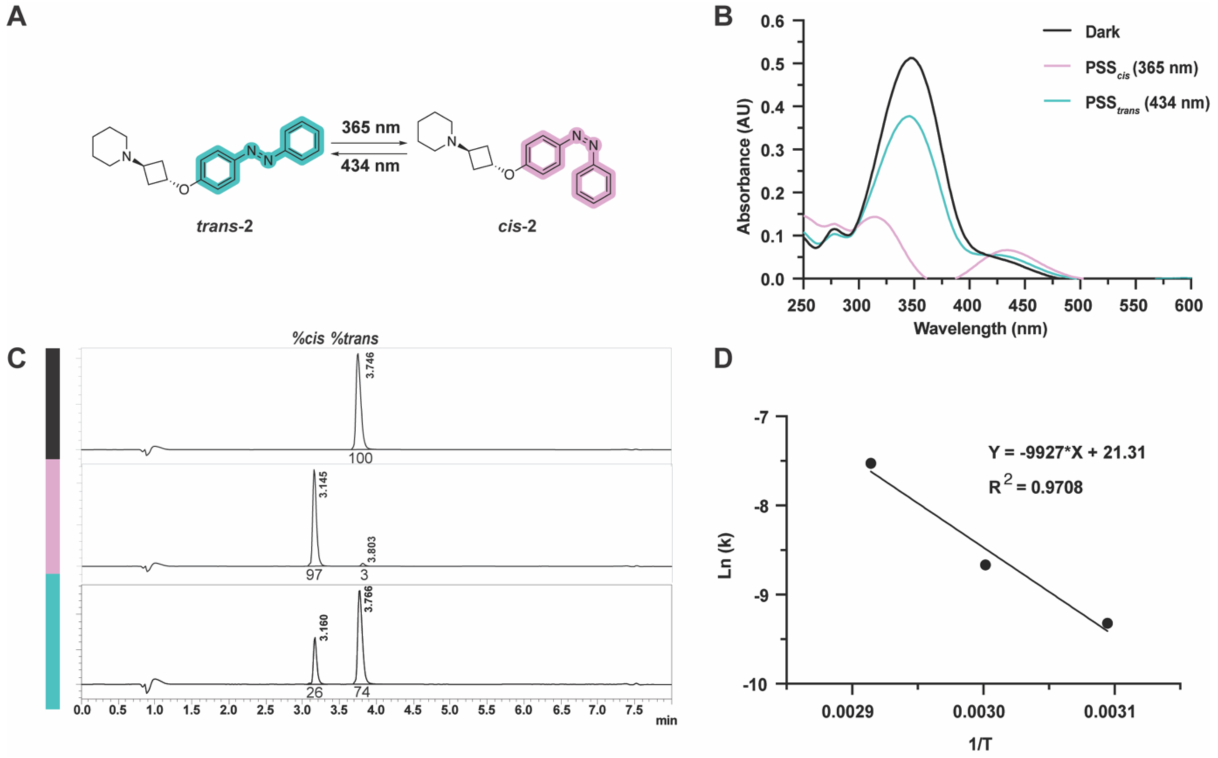

Figure S2. (A) Chemical structures of the *trans* and *cis* isomers of **2**. (B) UV-vis spectra of 25 µM of **2** in HBSS buffer containing 1% DMSO as the *trans* isomer (black), after illumination with 365 ± 11 nm for 2 min to PSS_{cis} (magenta) and after subsequent illumination with 434 ± 9 nm for 4 min to PSS_{trans} (cyan). (C) *Trans*-**2** (upper panel) and photostationary state (PSS) area percentages after illumination with 365 ± 11 nm for 10 min to reach PSS_{cis} (middle panel) and after subsequent illumination with 434 ± 9 nm for 10 min to reach PSS_{trans} (lower panel) at 10 mM in DMSO as determined by LC-MS analysis at the isosbestic point (295 nm). (D) Arrhenius fit for the thermal relaxation of **2** at three different temperatures (50 °C, 60 °C, 70 °C), as measured at 25 µM in HBSS buffer containing 1% DMSO.

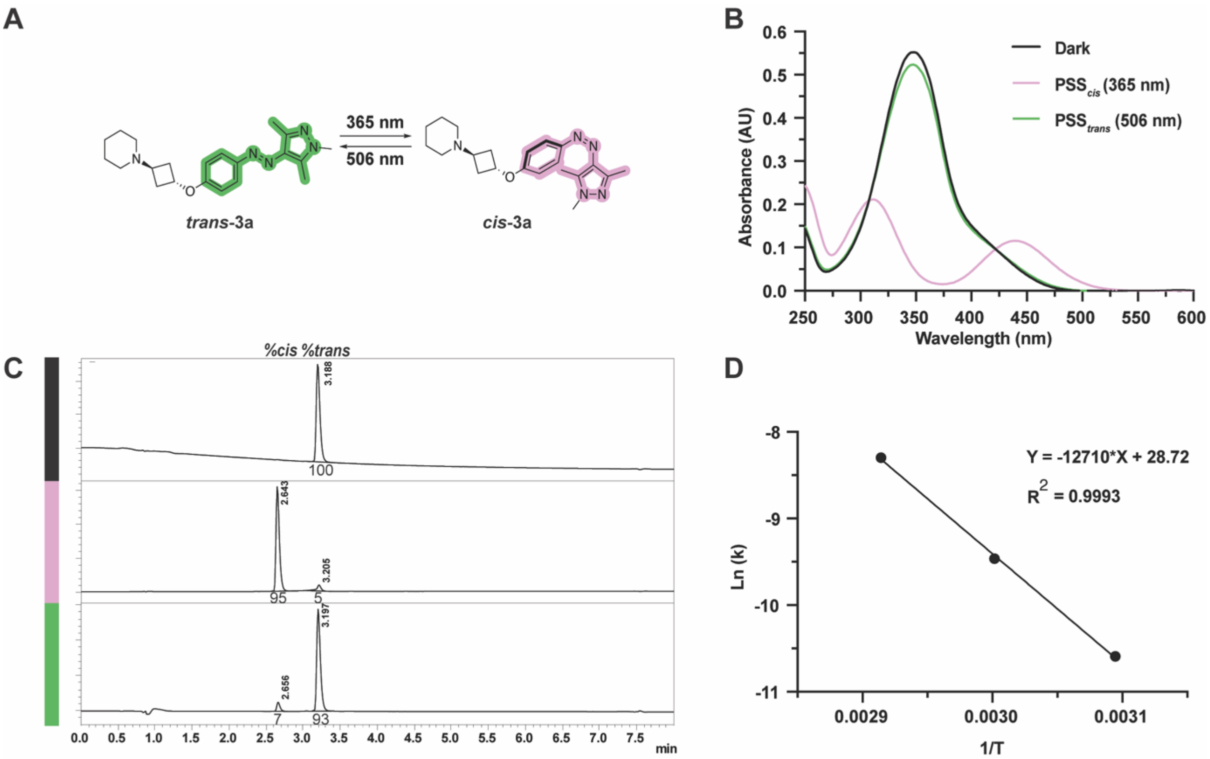

Figure S3. (A) Chemical structures of the *trans* and *cis* isomers of **3a**. (B) UV-vis spectra of 25 µM of **3a** in HBSS buffer containing 1% DMSO as the *trans* isomer (black), after illumination with 365 ± 11 nm for 2 min to PSS_{cis} (magenta) and after subsequent illumination with 506 ± 18 nm for 4 min to PSS_{trans} (green). (C) *Trans*-**3a** (upper panel) and photostationary state (PSS) area percentages after illumination with 365 ± 11 nm for 5 min to reach PSS_{cis} (middle panel) and after subsequent illumination with 506 ± 18 nm for 10 min to reach PSS_{trans} (lower panel) at 10 mM in DMSO as determined by LC-MS analysis at the isosbestic point (305 nm). (D) Arrhenius fit for the thermal relaxation of **3a** at three different temperatures (50 °C, 60 °C, 70 °C), as measured at 25 µM in HBSS buffer containing 1% DMSO.

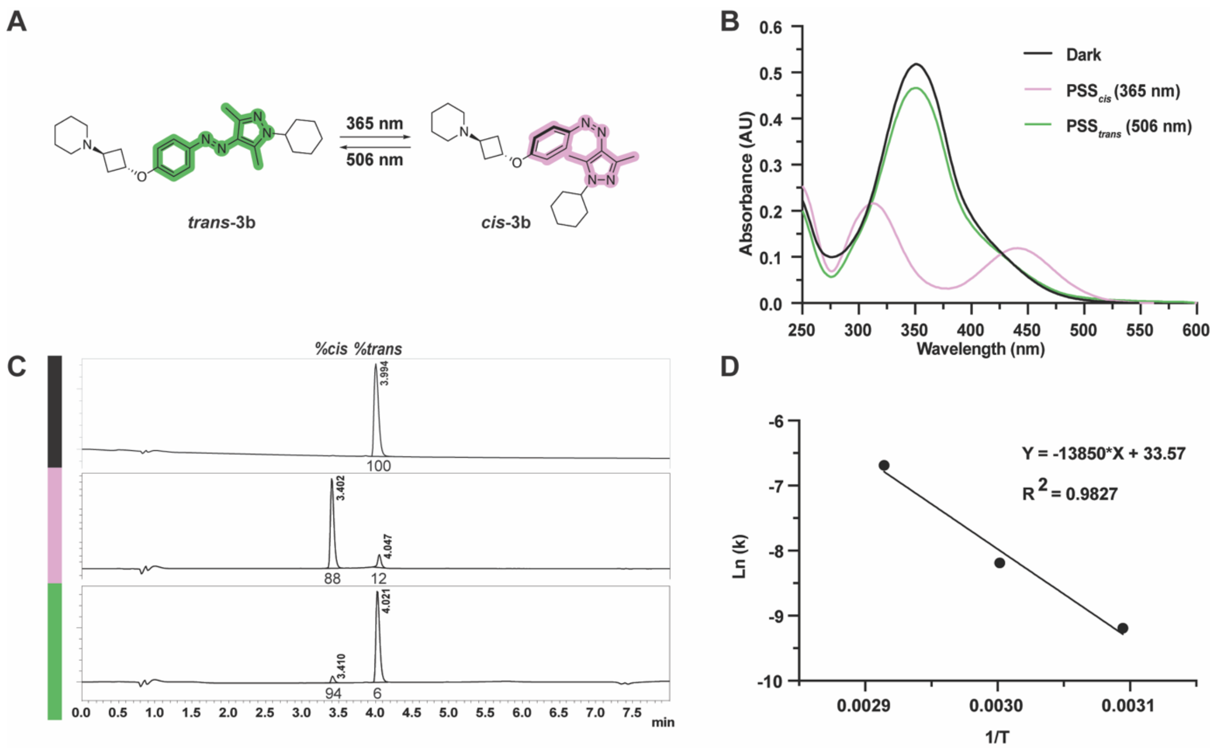

Figure S4. (A) Chemical structures of the *trans* and *cis* isomers of **3b**. (B) UV-vis spectra of 25 µM of **3b** in HBSS buffer containing 1% DMSO as the *trans* isomer (black), after illumination with 365 ± 11 nm for 2 min to PSS$_{cis}$ (magenta) and after subsequent illumination with 506 ± 18 nm for 4 min to PSS$_{trans}$ (green). (C) *Trans*-**3b** (upper panel) and photostationary state (PSS) area percentages after illumination with 365 ± 11 nm for 5 min to reach PSS$_{cis}$ (middle panel) and after subsequent illumination with 506 ± 18 nm for 10 min to reach PSS$_{trans}$ (lower panel) at 10 mM in DMSO as determined by LC-MS analysis at the isosbestic point (310 nm). (D) Arrhenius fit for the thermal relaxation of **3b** at three different temperatures (50 °C, 60 °C, 70 °C), as measured at 25 µM in HBSS buffer containing 1% DMSO.

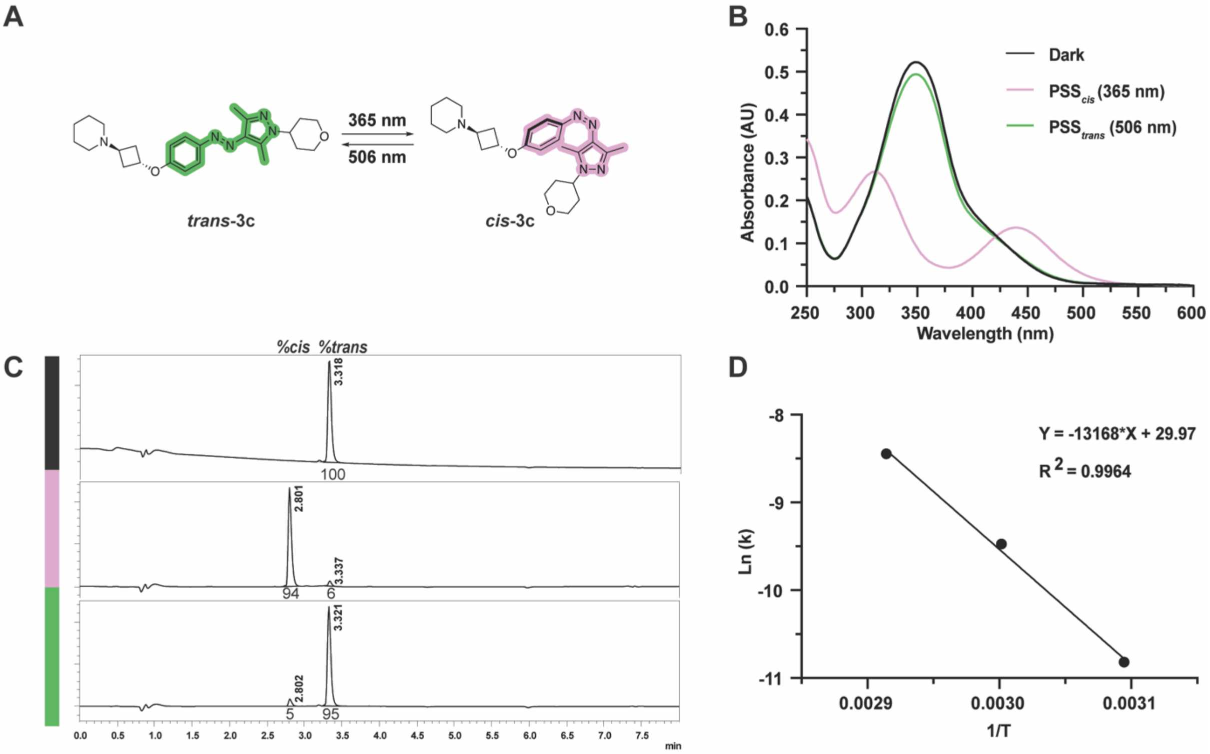

Figure S5. (A) Chemical structures of the *trans* and *cis* isomers of **3c**. (B) UV-vis spectra of 25 µM of **3c** in HBSS buffer containing 1% DMSO as the *trans* isomer (black), after illumination with 365 ± 11 nm for 2 min to PSS_{cis} (magenta) and after subsequent illumination with 506 ± 18 nm for 4 min to PSS_{trans} (green). (C) *Trans*-**3c** (upper panel) and photostationary state (PSS) area percentages after illumination with 365 ± 11 nm for 5 min to reach PSS_{cis} (middle panel) and after subsequent illumination with 506 ± 18 nm for 10 min to reach PSS_{trans} (lower panel) at 10 mM in DMSO as determined by LC-MS analysis at the isosbestic point (310 nm). (D) Arrhenius fit for the thermal relaxation of **3c** at three different temperatures (50 °C, 60 °C, 70 °C), as measured at 25 µM in HBSS buffer containing 1% DMSO.

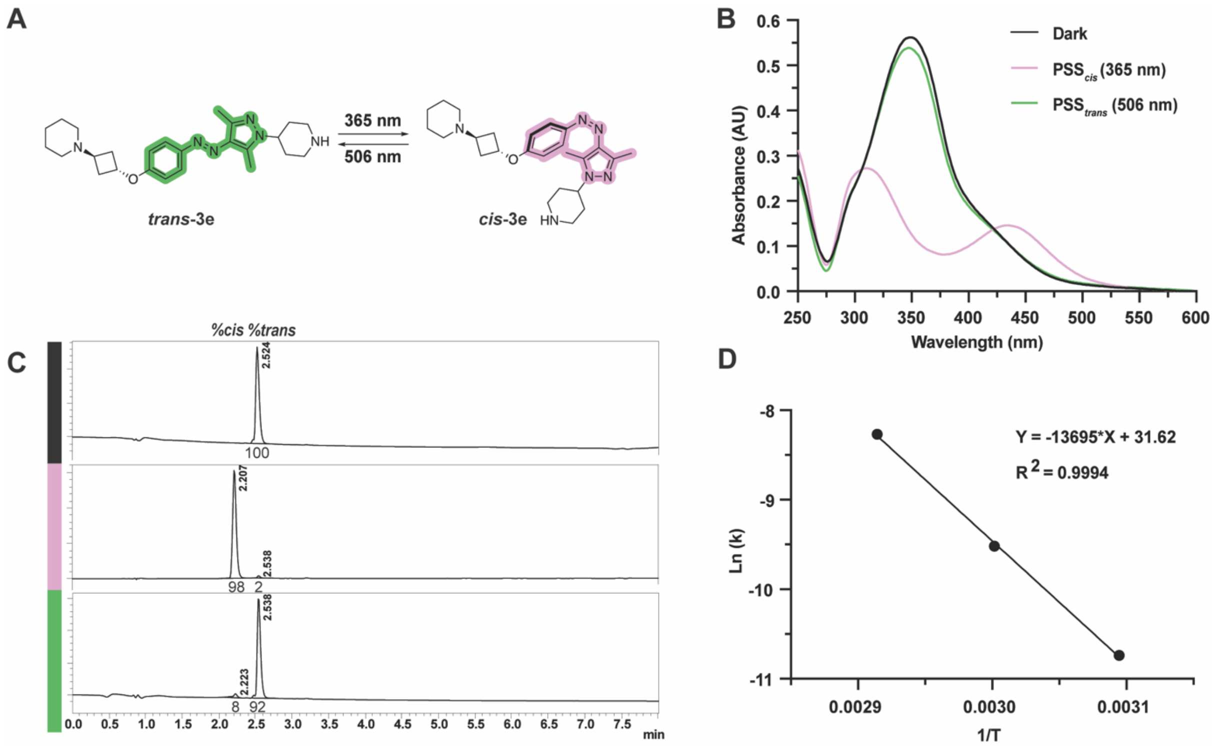

Figure S6. (A) Chemical structures of the *trans* and *cis* isomers of **3e**. (B) UV-vis spectra of 25 µM of **3e** in HBSS buffer containing 1% DMSO as the *trans* isomer (black), after illumination with 365 ± 11 nm for 2 min to PSS_{cis} (magenta) and after subsequent illumination with 506 ± 18 nm for 4 min to PSS_{trans} (green). (C) *Trans*-**3e** (upper panel) and photostationary state (PSS) area percentages after illumination with 365 ± 11 nm for 5 min to reach PSS_{cis} (middle panel) and after subsequent illumination with 506 ± 18 nm for 10 min to reach PSS_{trans} (lower panel) at 10 mM in DMSO as determined by LC-MS analysis at the isosbestic point (305 nm). (D) Arrhenius fit for the thermal relaxation of **3e** at three different temperatures (50 °C, 60 °C, 70 °C), as measured at 25 µM in HBSS buffer containing 1% DMSO.

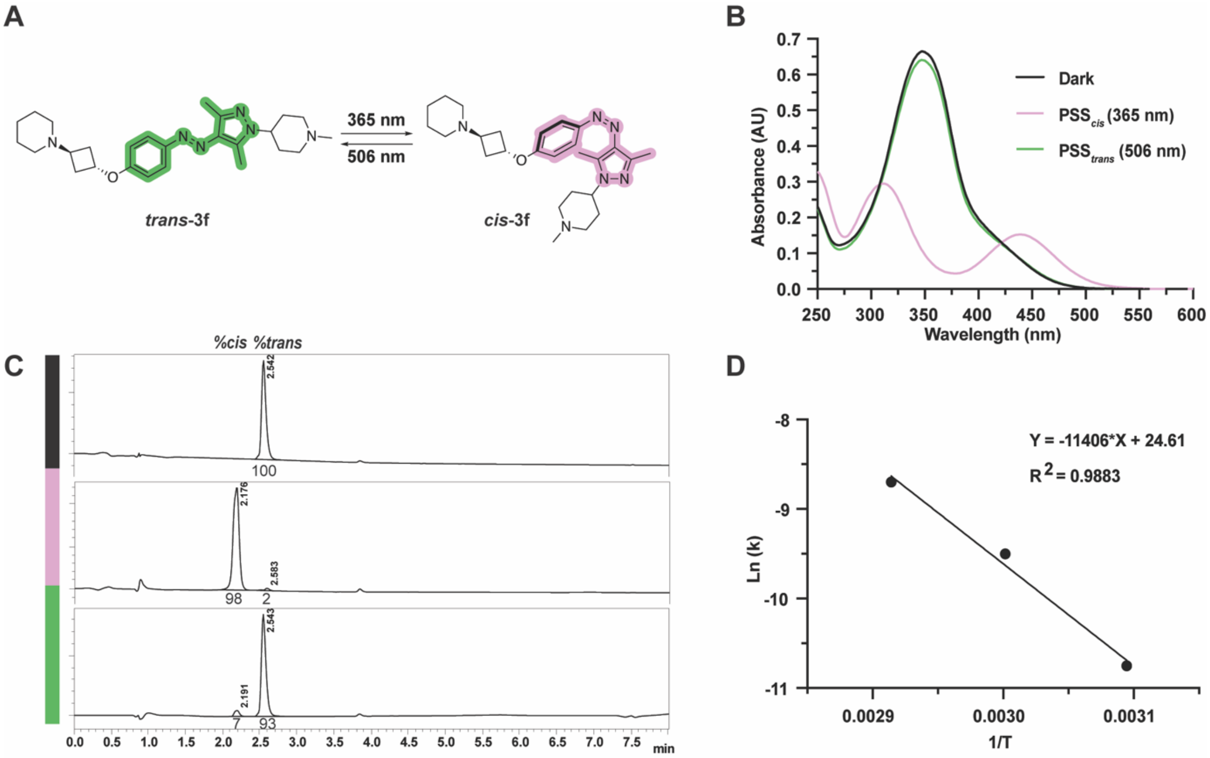

Figure S7. (A) Chemical structures of the *trans* and *cis* isomers of **3f**. (B) UV-vis spectra of 25 µM of **3f** in HBSS buffer containing 1% DMSO as the *trans* isomer (black), after illumination with 365 ± 11 nm for 2 min to PSS$_{cis}$ (magenta) and after subsequent illumination with 506 ± 18 nm for 4 min to PSS$_{trans}$ (green). (C) *Trans*-**3f** (upper panel) and photostationary state (PSS) area percentages after illumination with 365 ± 11 nm for 5 min to reach PSS$_{cis}$ (middle panel) and after subsequent illumination with 506 ± 18 nm for 10 min to reach PSS$_{trans}$ (lower panel) at 10 mM in DMSO as determined by LC-MS analysis at the isosbestic point (305 nm). (D) Arrhenius fit for the thermal relaxation of **3f** at three different temperatures (50 °C, 60 °C, 70 °C), as measured at 25 µM in HBSS buffer containing 1% DMSO.

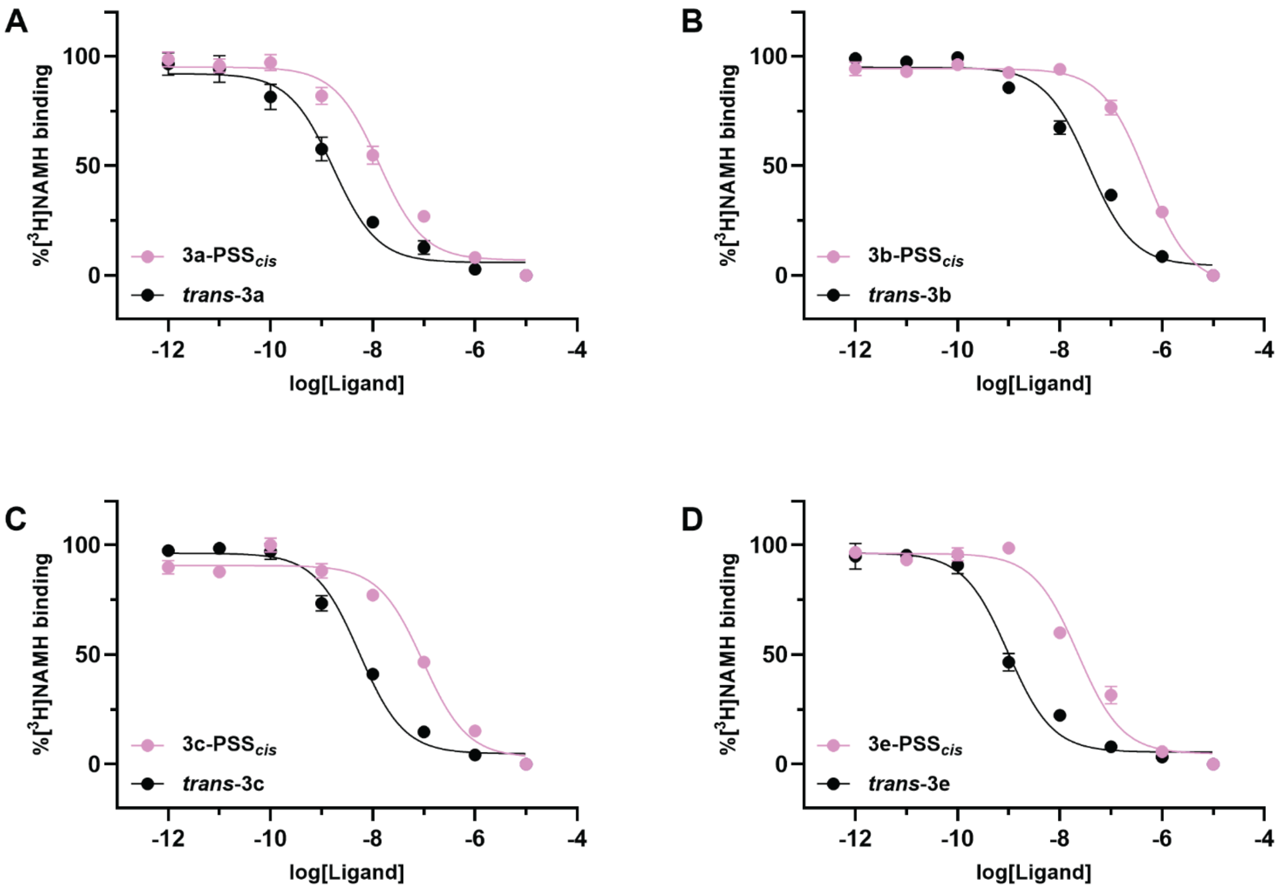

Figure S8. Radioligand binding experiments in competition with [^{3}H]NAMH. Binding curves of *trans* and PSS$_{cis}$ states of (A) **3a** (B) **3b** (C) **3c** and (D) **3e**. Pooled data of three independent experiments are shown as mean ± SEM.

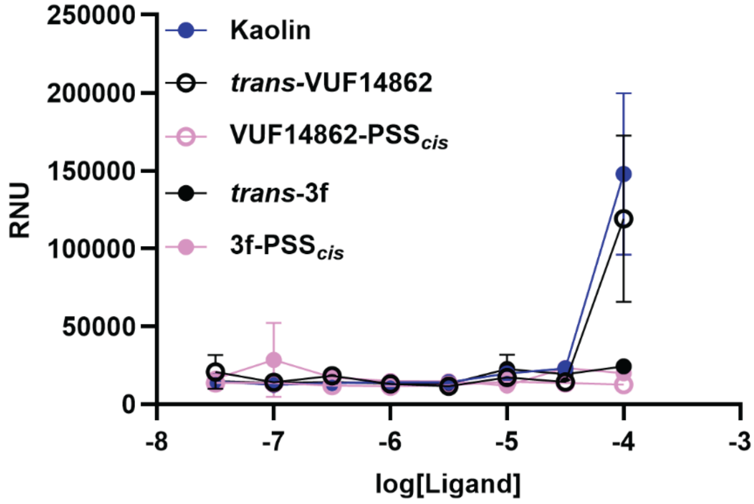

Figure S9. Nephelometry data for **3f**, VUF14862 (first-generation H_3R photoswitchable antagonist[3]) and the kaolin control. Mean of triplicates ± SD shown.

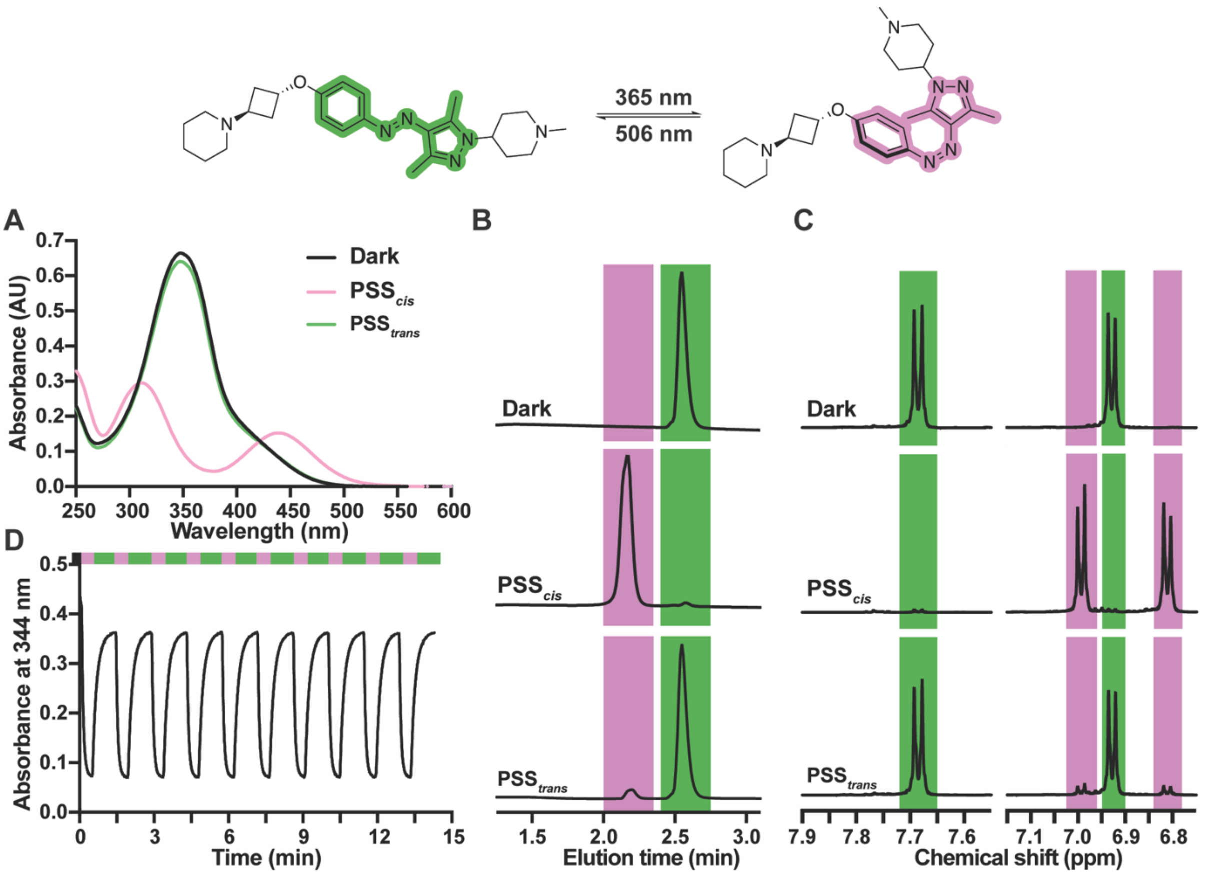

Figure S10. Photochemical properties of **3f**. (A) UV-vis spectra of 25 µM **3f** in HBSS buffer containing 1% DMSO in the dark, after illumination to PSS_{cis} with 365 ± 10 nm for 2 min and after subsequent illumination to PSS_{trans} with 506 ± 20 nm for 2 min. (B) Representative parts of LC−MS chromatograms of **3f** in the dark, after illumination with 365 ± 10 nm for 300 s to reach PSS_{cis} and subsequently with 506 ± 20 nm for 300 s to reach PSS_{trans} (full chromatograms are shown in Figure S11). (C) Part of ^{1}H NMR spectra of 10 mM **3f** in DMSO-d_6 (representing the two aromatic signals) in the dark, after illumination with 365 ± 10 nm for 600 s to reach PSS_{cis} and subsequently 506 ± 20 nm for 900 s to reach PSS_{trans} (full spectra are shown in Figure S12). (D) Reversible isomerization cycles of 25 µM **3f** in HBSS buffer containing 1% DMSO, repeatedly illuminated for 30 s with 365 ± 10 nm to reach PSS_{cis} and subsequently for 60 s with 506 ± 20 nm to reach PSS_{trans}. The y-axis shows the absorbance at 344 nm.

Ligand **3f** shows a good band separation of the n–π* transition bands of the *trans* and PSS_{cis} states in the 400-550 nm area (Figure S10A). This allows efficient *trans*-PSS_{cis} photoswitching in HBSS buffer using UV light (365 nm) and PSS_{cis}-PSS_{trans} back-switching with green light (506 nm). LC-MS analysis (Figure S10B, S11) and, more quantitively, ^{1}H NMR analysis (Figure S10C, S12) reveal high PSS_{cis} and PSS_{trans} values for **3f** (98% and >95% *cis* isomer for PSS_{cis} by LC-MS and ^{1}H NMR analysis, respectively; 93% and 93% *trans* isomer for PSS_{trans} by LC-MS and ^{1}H NMR analysis, respectively). These analyses also confirmed that no photodecomposition occurs upon illumination with either 365 or 506 nm. Reversible isomerization cycles could be conducted in HBSS buffer without any photobleaching (Figure S10D). After irradiation for 30 s with 365 nm, the PSS_{cis} state was reached. Subsequently, the PSS_{trans} state was obtained after irradiation for 60 s with 506 nm. This indicates that the switching processes from *trans* to PSS_{cis} states and from PSS_{cis} to PSS_{trans} states are fast, with half-lives of 3.1 and 7.3 s, respectively.

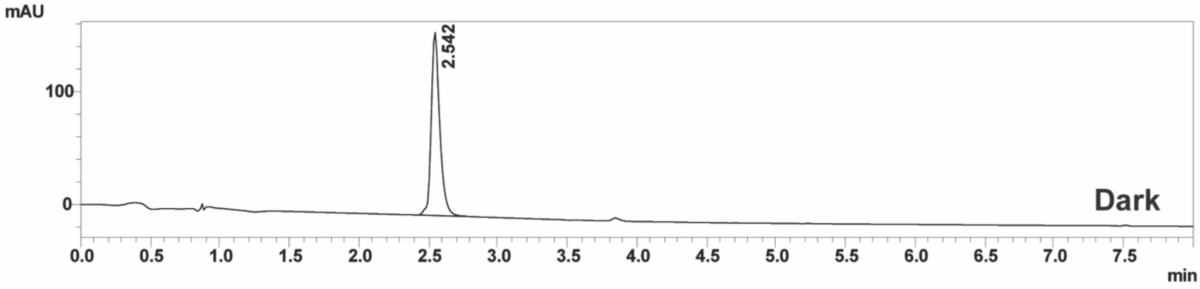

PeakTable

PDA Ch1 305nm 4nm

Peak#	Ret. Time	Area	Height	Name	Area %
1	2.542	696505	161767		100.000
Total		696505	161767		100.000

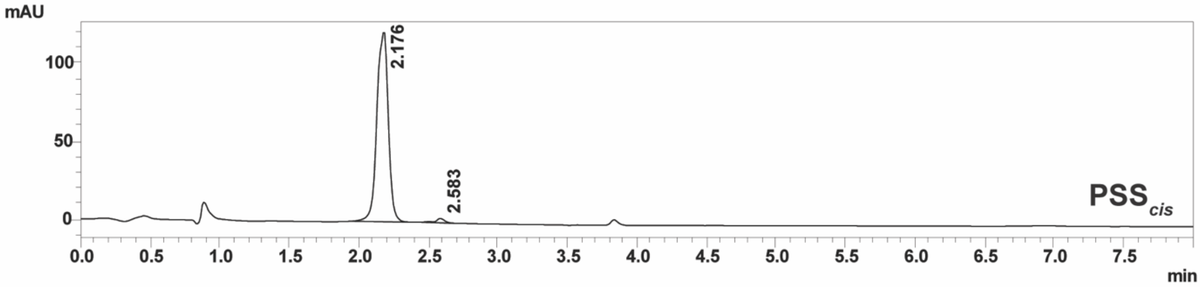

PeakTable

PDA Ch1 305nm 4nm

Peak#	Ret. Time	Area	Height	Name	Area %
1	2.176	689417	120995		98.211
2	2.583	12560	2733		1.789
Total		701977	123728		100.000

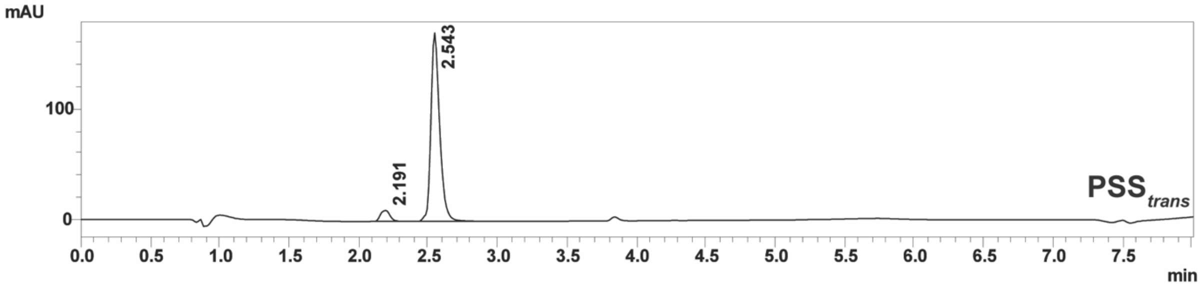

PeakTable

PDA Ch1 305nm 4nm

Peak#	Ret. Time	Area	Height	Name	Area %
1	2.191	47339	9857		6.036
2	2.543	736894	169833		93.964
Total		784233	179690		100.000

Figure S11. Full LC chromatograms of 10 mM **3f** in DMSO-d$_6$ in the dark, after irradiation with 365 ± 11 nm for 5 min to reach PSS$_{cis}$ and subsequently with 506 ± 18 nm for 10 min to reach PSS$_{trans}$.

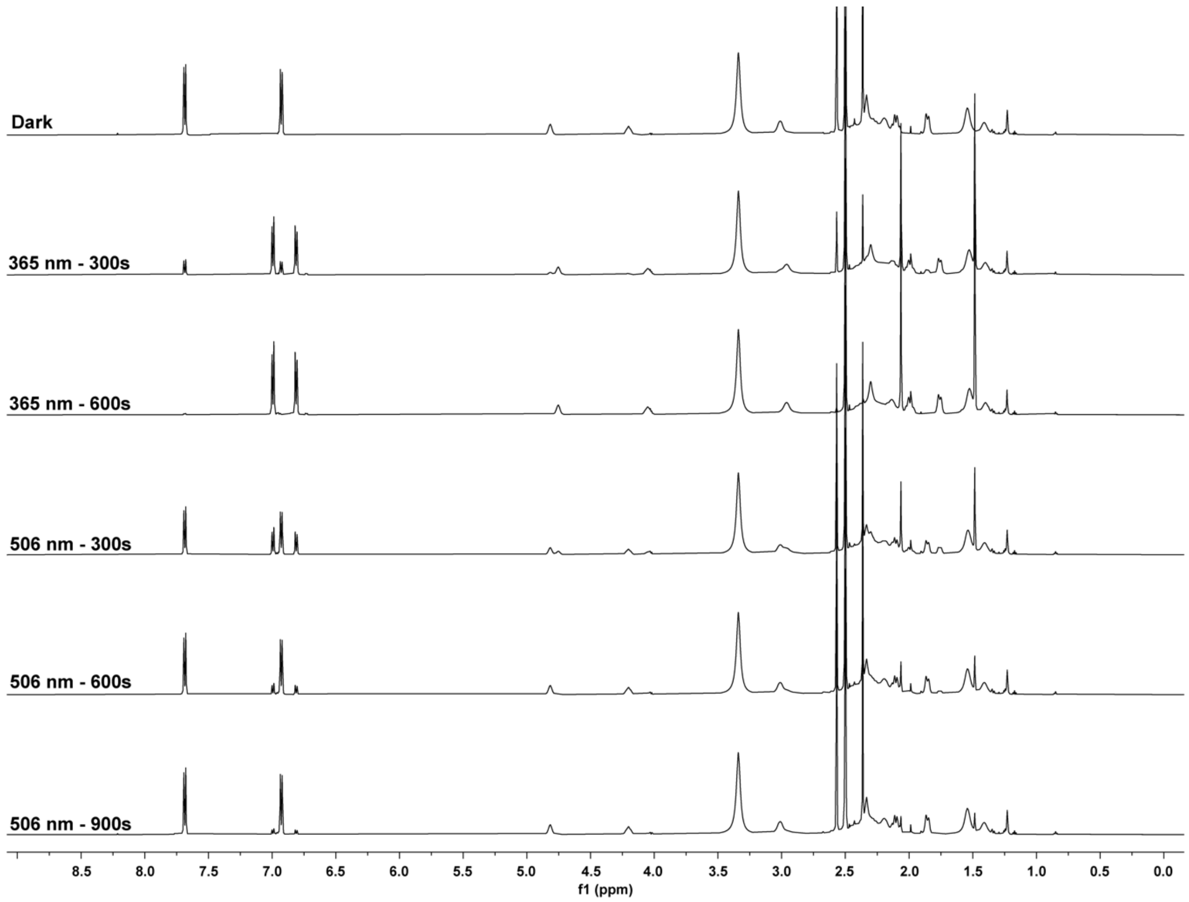

Figure S12. Full NMR spectra of 10 mM **3f** in DMSO-d$_6$ in the dark, after illumination with 365 ± 11 nm at 300 s and 600 s to reach PSS$_{cis}$, and subsequently with 506 ± 18 nm at 300 s, 600 s and 900 s to reach PSS$_{trans}$.

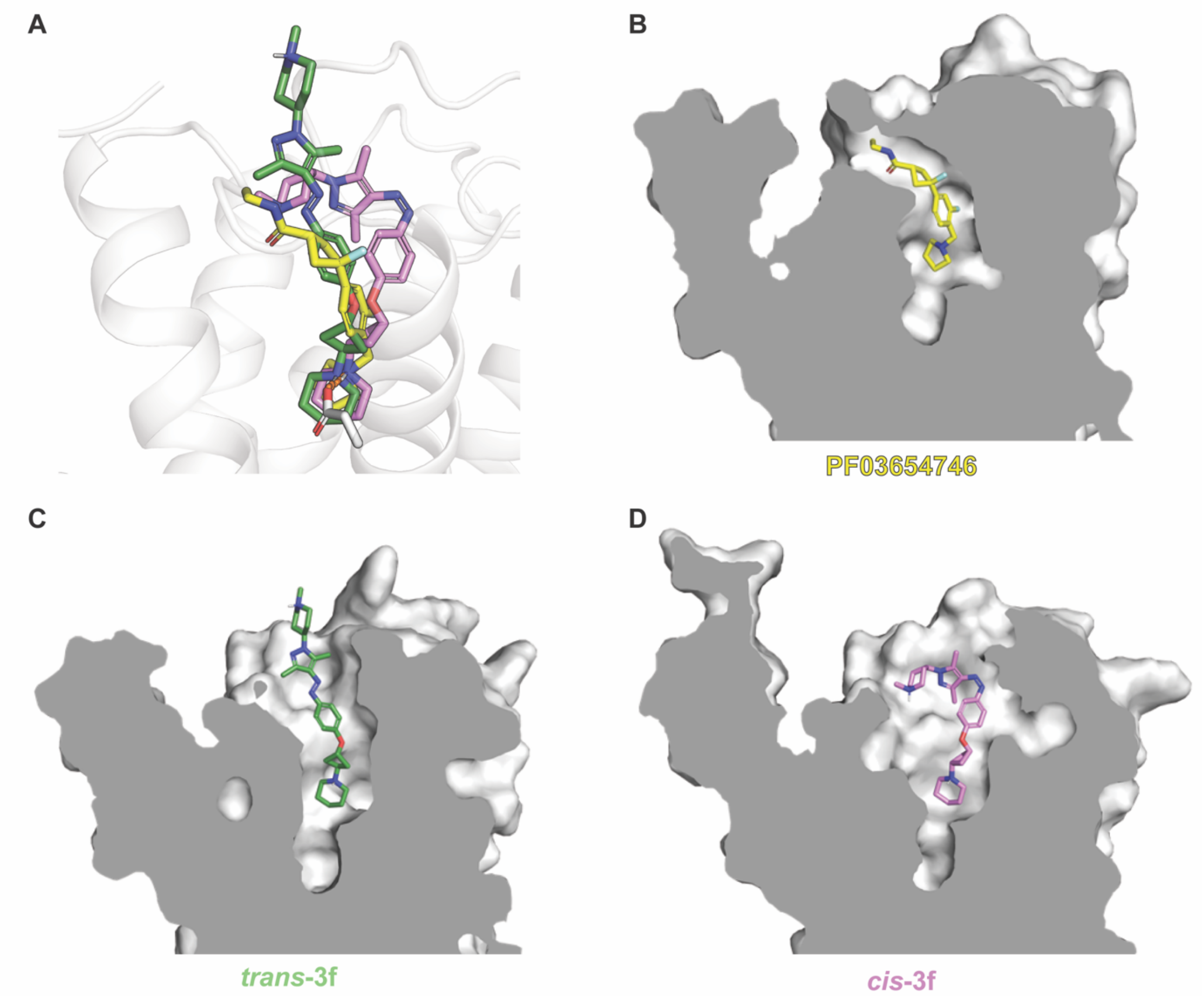

Figure S13. (A) Overlay of the binding pose of PF03654746 taken from its X-ray crystal structure (PDB: 7F61[4]) and representative snapshots of **3f** isomers from MD simulations. (B-D) Comparison of ligand binding pocket sizes for (B) PF03654746 (yellow carbon atoms, X-ray pose), (C) *trans*-**3f** (green carbon atoms, representative MD snapshot), and (D) *cis*-**3f** (magenta carbon atoms, representative MD snapshot).

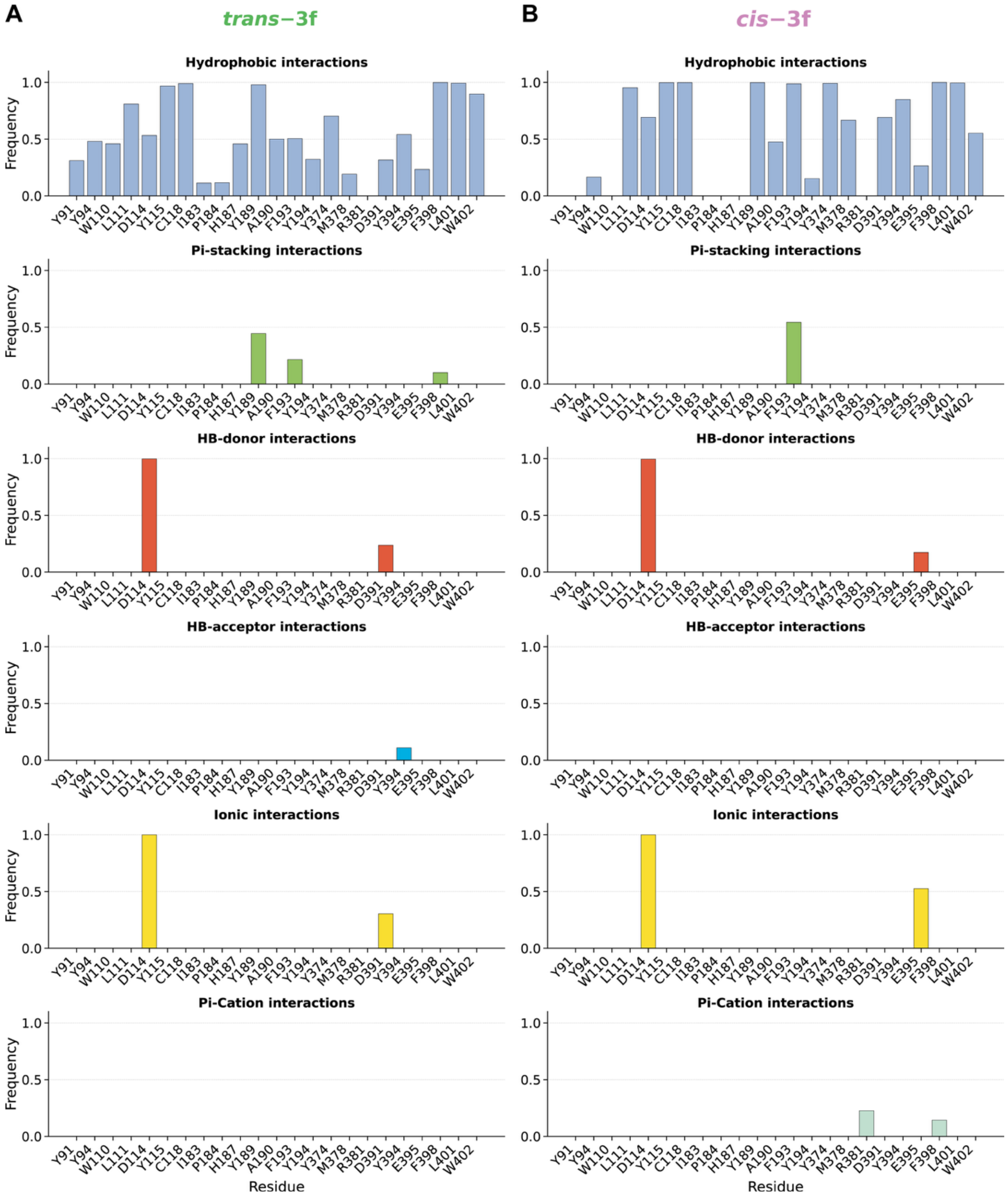

Figure S14. Average ligand-protein interactions from four replicas of MD simulations of (A) *trans*-**3f** and (B) *cis*-**3f**. Interactions are calculated by concatenation of the second half (500 ns) of each replica.

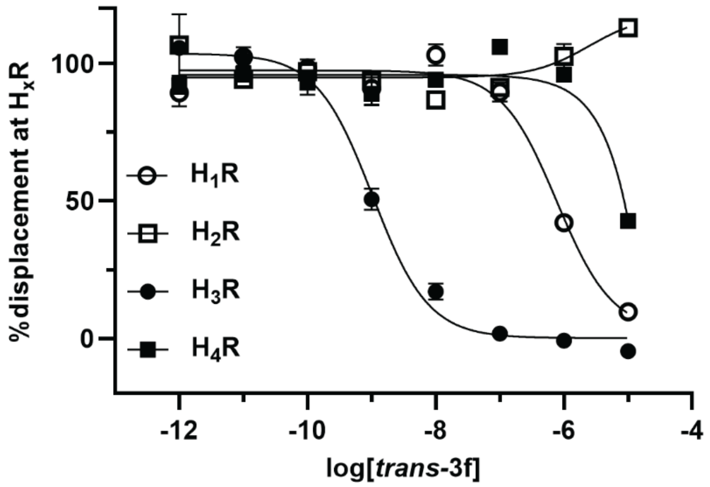

Figure S15. Histamine receptor selectivity binding experiments. Concentration binding curves of *trans*-**3f** on cell homogenates expressing H_1R (in competition with 4.0-4.8 nM [^{3}H]mepyramine) or H_4R (competition with 1.9-2.4 nM [^{3}H]histamine. H_2R binding was measured in live HEK293T cells stably expressing Nluc-H_2R (in competition with 50 nM UR-KAT478). Pooled data of three independent experiments are shown as mean ± SEM.

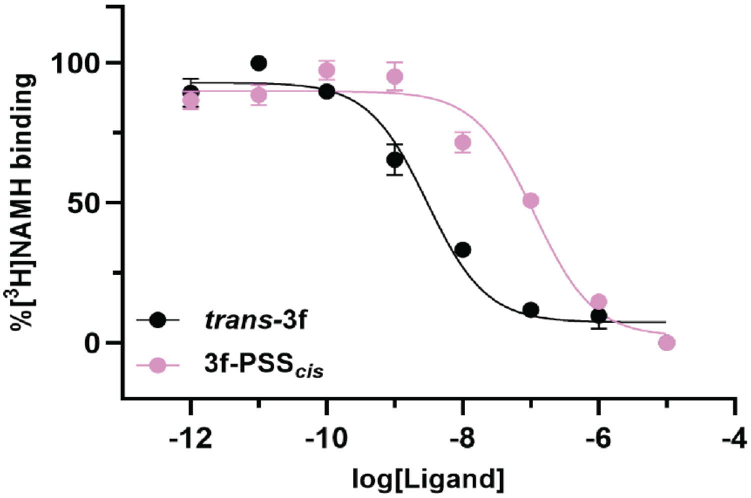

Figure S16. Radioligand binding experiments with **3f** on mH_3R-expressing cell homogenates in competition with [^{3}H]NAMH (1.4-1.7 nM). Pooled data of three independent experiments are shown as mean ± SEM.

Table S1. Pharmacological parameters and associated switching conditions of **3f** from the functional NanoBit-PKA assay in HEK293T cells

**3f** states			
Immethridine pEC$_{50}$	*trans*-**3f** pIC$_{50}$	**3f**-PSS$_{cis}$ pIC$_{50}$	Fold IC$_{50}$ shift
9.1 ± 0.1	7.5 ± 0.1	6.4 ± 0.1	12.6
Dynamic *trans*-**3f** switching			
Consecutive steps	$\lambda_{irradiation}$	pIC$_{50}$	
**1**	None	7.7 ± 0.2	
**2**	365 nm 10 min	6.1 ± 0.0	
**3**	500 nm 10 min	7.3 ± 0.1	
**4**	365 nm 10 min	6.4 ± 0.1	
**5**	500 nm 10 min	7.3 ± 0.1	

Data obtained either using *trans*-**3f** or **3f**-PSS$_{cis}$ (obtained by pre-illuminating *trans*-**3f**) in competition with 10 nM immethridine after a 15-minute incubation period, or dynamic setup, in which values are obtained either in the dark, or after 365 or 500 nm consecutive irradiation steps (1-5). Data are shown as mean ± SEM of three independent experiments.

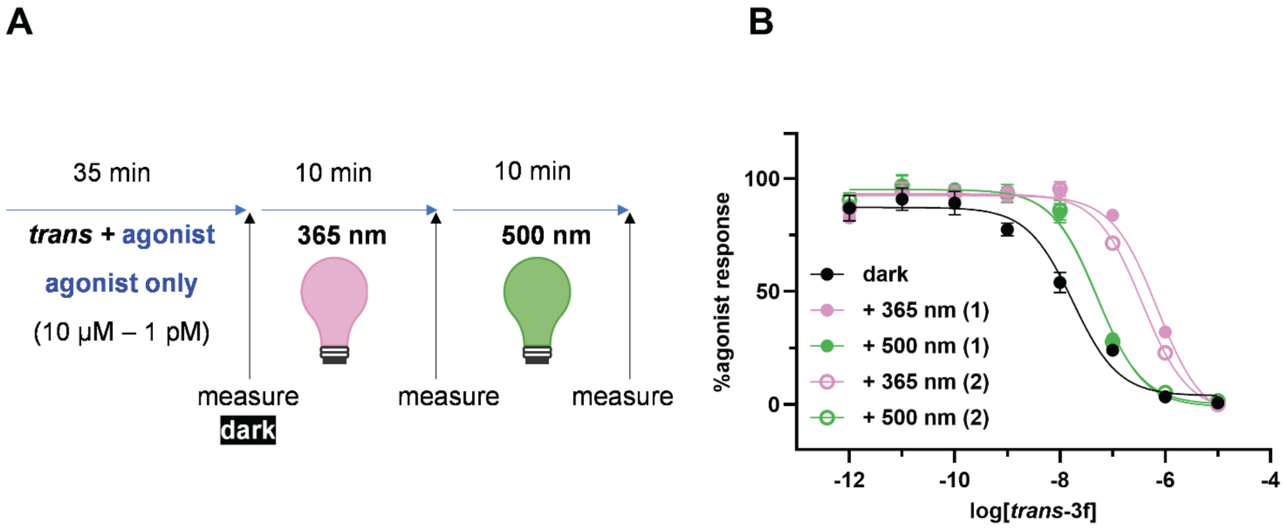

Figure S17. Functional dynamic light modulation of H_3R activity in HEK293T cells in a NanoBit-PKA assay (A) Schematic depiction of setup. (B) Concentration-response curves of **3f** in competition with immethridine (agonist, 10 nM) in the dark, after 365 or 500 nm irradiation cycles (1,2). Pooled data of three independent experiments are shown as mean ± SEM.

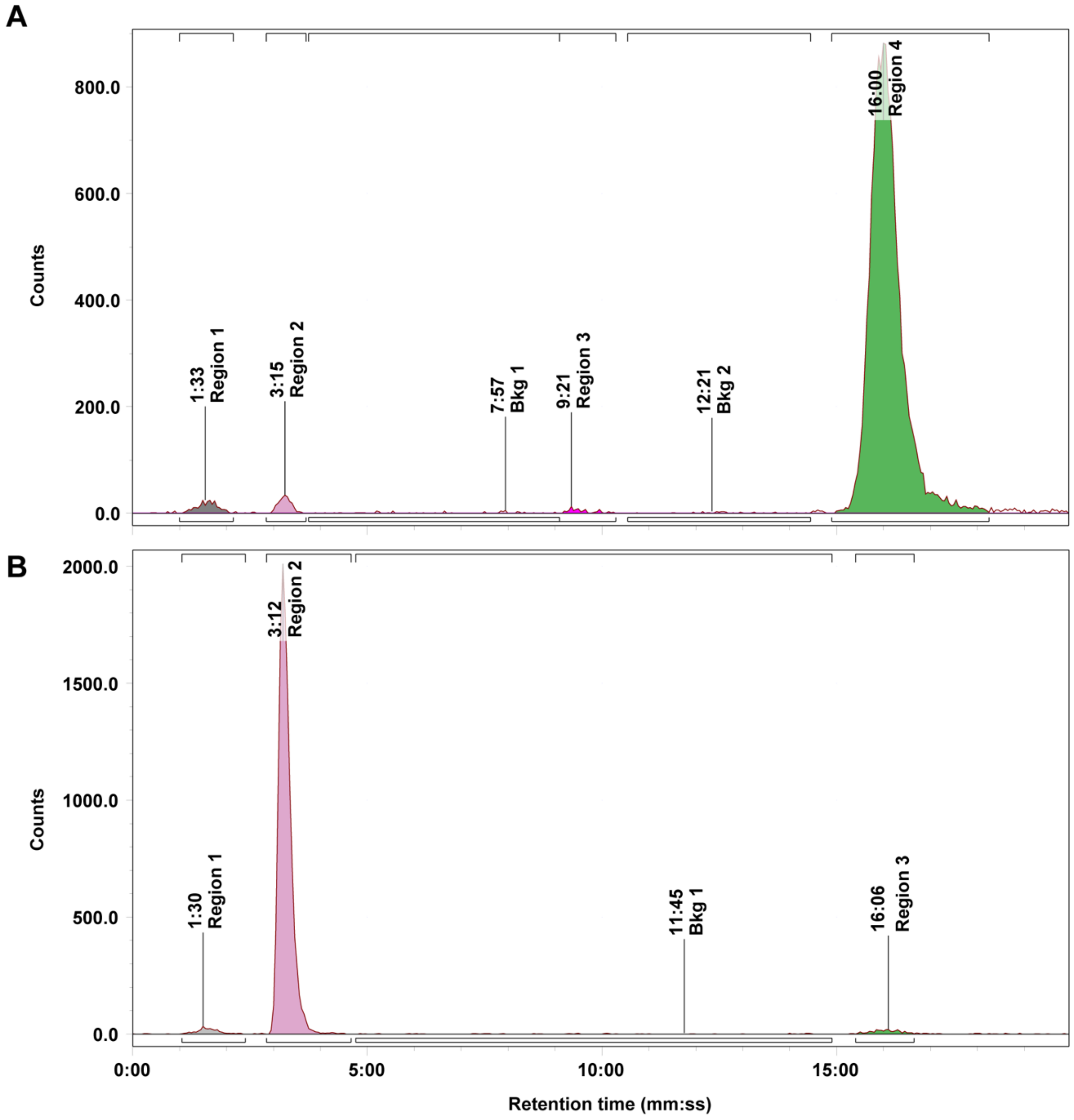

Figure S18. ^{3}H-chromatogram of (A) *trans*-[^{3}H]**3f** containing 96% of the *trans* isomer and 2% of the *cis* isomer and (B) [^{3}H]**3f** at PSS$_{cis}$ containing 2% of the *trans* isomer and 96% of the *cis* isomer. Photoswitching was performed in EtOH with a 100-fold dilution of the stock shown in Figure S21. The products were analyzed with HPLC (Jasco PU-2080 Pump, Jasco UV-2075 UV detector, Lablogic β-RAM Scintillation detector, XBridge C18, 4.6x100 mm, 100Å, 5µm, 45% acetonitrile, 55% water, 0.2% DIPEA, 1 ml/min, 254 nm). The UV trace is provided in Figure S19.

A

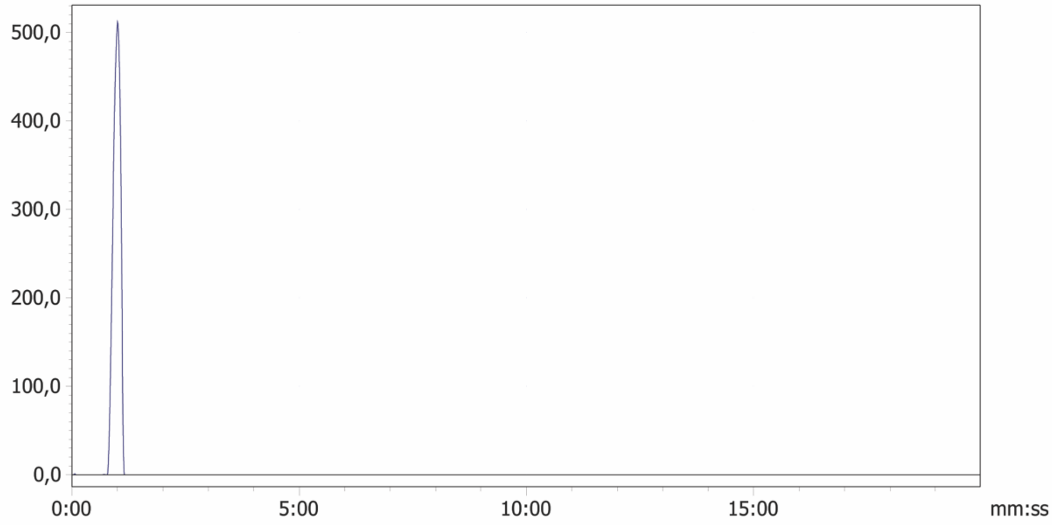

Regions: UV Detector:

Name	Start (mm:ss)	End (mm:ss)	Retention (mm:ss)	Height (mAU)	Area (mAU·s)	%ROI (%)
No ROIs allocated.						
0 Peaks					0,0	100,00

B

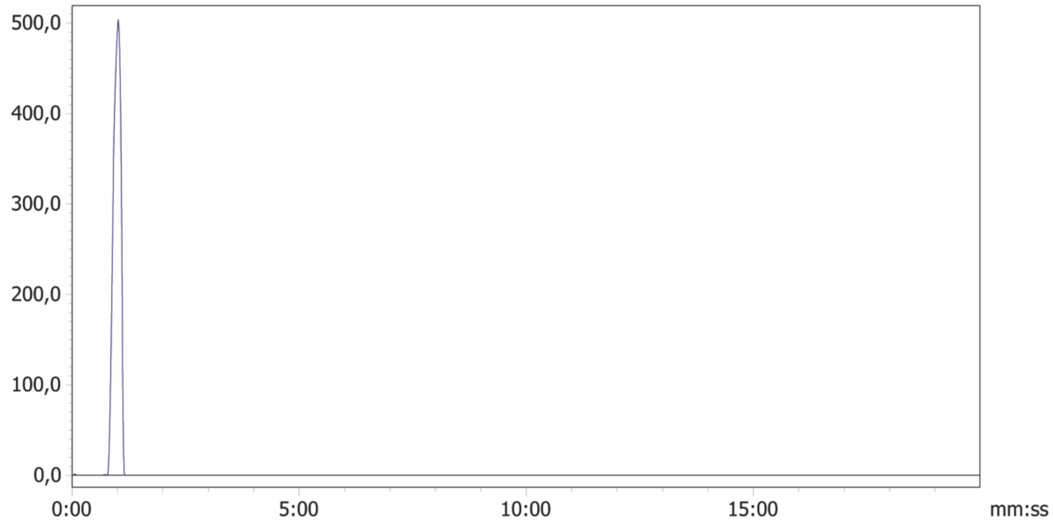

Regions: UV Detector:

Name	Start (mm:ss)	End (mm:ss)	Retention (mm:ss)	Height (mAU)	Area (mAU·s)	%ROI (%)
No ROIs allocated.						
0 Peaks					0,0	100,00

Figure S19. UV chromatogram with detection at 254 nm of (A) *trans*-[^{3}H]**3f** corresponding to the ^{3}H chromatogram shown in Figure S18A and (B) [^{3}H]**3f** at PSS$_{cis}$ corresponding to the ^{3}H chromatogram shown in Figure S18B. The peak at 1.0 min is the solvent front.

Table S2. Affinity values (pK_i) of non-labeled H_3R ligands in competition with *trans*-[^{3}H]**3f** or [^{3}H]NAMH.

trans-[^{3}H]**3f**				
**3f**-PSS_{cis}	*trans*-**3f**	clobenpropit	thioperamide	immepip
7.7 ± 0.0	9.0 ± 0.1	9.2 ± 0.2	6.7 ± 0.1	8.5 ± 0.1
[^{3}H]NAMH				
**3f**-PSS_{cis}	*trans*-**3f**	clobenpropit	thioperamide	immepip
7.6 ± 0.2	9.3 ± 0.1	9.3 ± 0.1	7.2 ± 0.2*	9.3 ± 0.1*

The pK_i values are shown as mean ± SEM of three independent radioligand binding assays on hH_3R-expressing cell homogenates. *Data obtained from *Mocking et al.*[5]

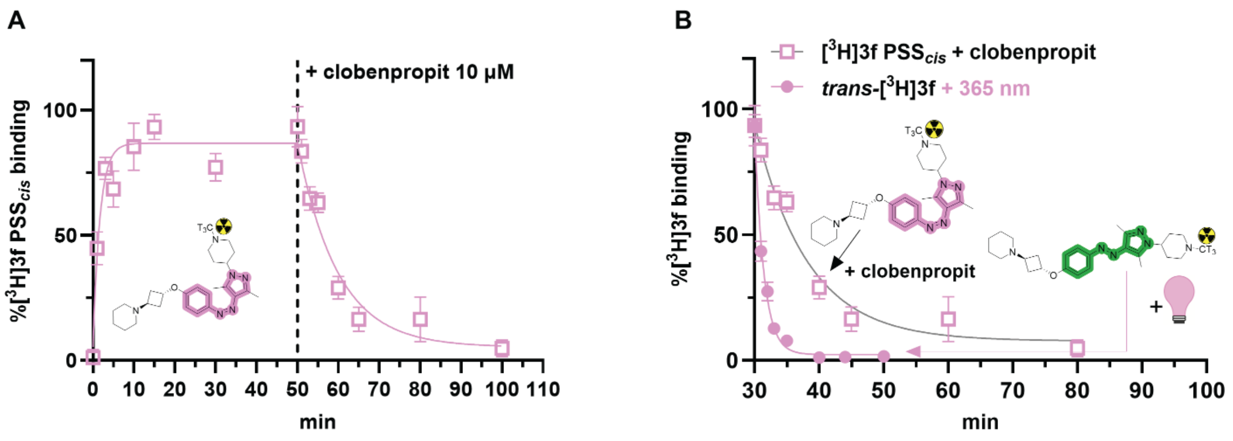

Figure S20. H_3R binding kinetics of [^{3}H]**3f** (A) Association and clobenpropit-dependent dissociation curves of PSS_{cis} (B) Comparison of clobenpropit-dependent dissociation of PSS_{cis} and 365 nm-dependent dissociation of the *trans* isomer. Pooled data of three independent experiments are shown as mean ± SEM.

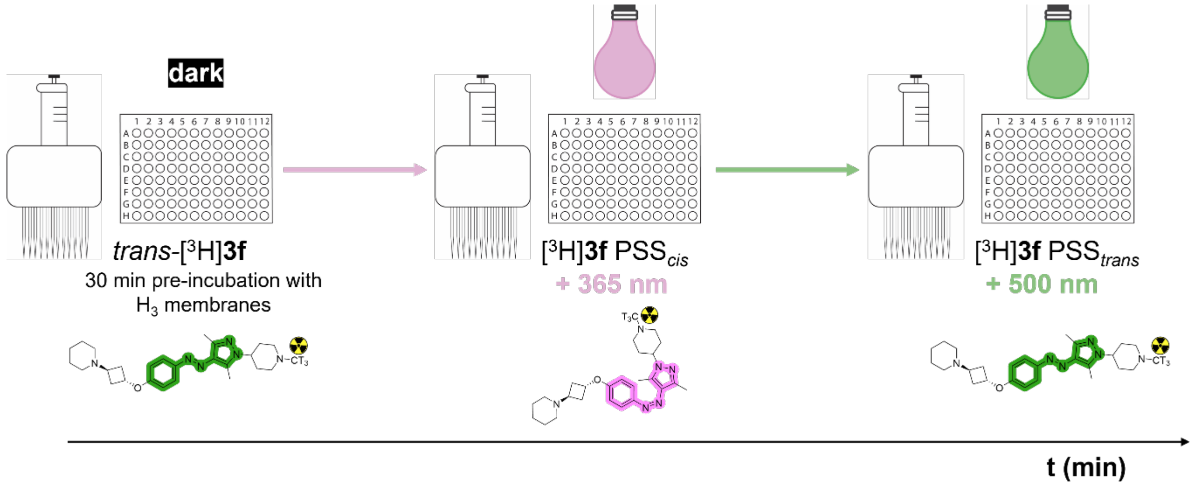

Scheme S2. Stepwise overview of the dynamic radioligand binding assay. Dark and light-dependent binding of *trans*-[^{3}H]**3f** was assessed in H_3R-expressing cell homogenates.

A Chromatogram: ^{3}H

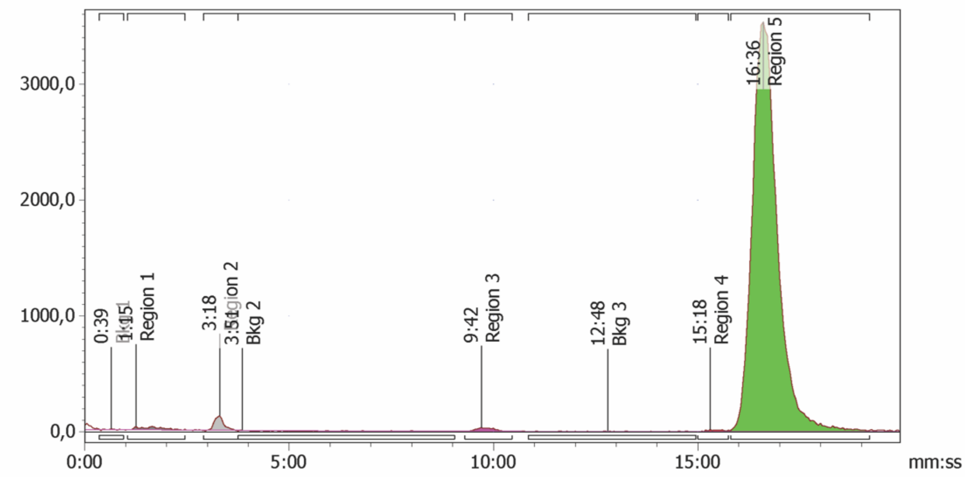

Regions: ^{3}H Detector: ß-RAM

Name	Start (mm:ss)	End (mm:ss)	Retention (mm:ss)	Height (Counts)	Area (Counts)	%ROI (%)
Bkg 1	0:21	0:57	0:39	26,0		
Region 1	1:03	2:27	1:15	48,0	339,4	0,66
Region 2	2:54	3:45	3:18	140,0	649,3	1,25
Bkg 2	3:45	9:03	3:51	15,0		
Region 3	9:18	10:27	9:42	37,0	316,2	0,61
Bkg 3	10:51	14:57	12:48	8,0		
Region 4	15:00	15:45	15:18	20,0	125,9	0,24
Region 5	15:48	19:12	16:36	3534,0	50336,8	97,24
5 Peaks					51767,6	100,00

B Chromatogram: UV

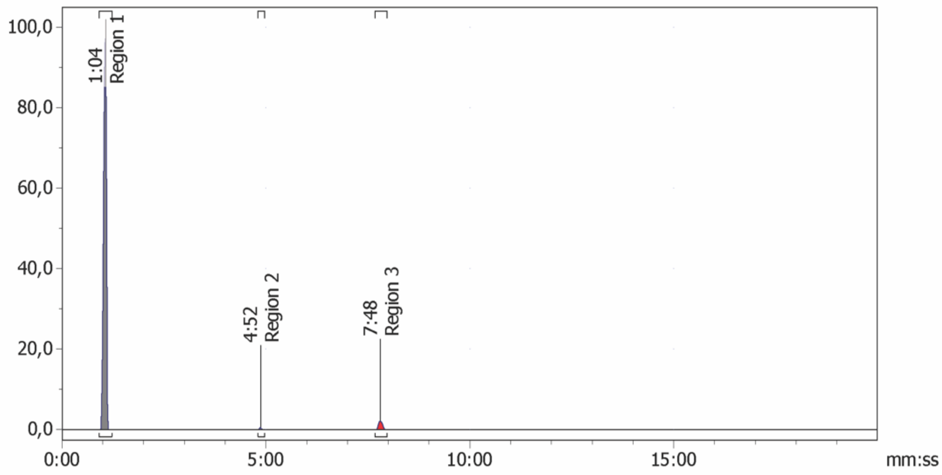

Regions: UV Detector:

Name	Start (mm:ss)	End (mm:ss)	Retention (mm:ss)	Height (mAU)	Area (mAU·s)	%ROI (%)
Region 1	0:54	1:13	1:04	101,8	503,1	96,99
Region 2	4:48	4:58	4:52	0,6	1,4	0,27
Region 3	7:40	7:58	7:48	2,1	14,2	2,74
3 Peaks					518,7	100,00

Figure S21. (A) ^{3}H-chromatogram and (B) UV chromatogram of the *trans*-[^{3}H]**3f** stock. The products were analyzed with HPLC (Jasco PU-2080 Pump, Jasco UV-2075 UV detector, Lablogic β-RAM Scintillation detector, XBridge C18, 4.6x100 mm, 100Å, 5µm, 45% acetonitrile, 55% water, 0.2% DIPEA, 1 ml/min, 254 nm). The peak at 1.0 min is the solvent front. A minor UV impurity was observed at 7.48 min.

Experimental procedures - Pharmacology

Materials

All radioligands mentioned in this paper (except [^{3}H]**3f**) were purchased from Perkin Elmer. UR-KAT478[6] was a kind gift from the Pockes group, University of Regenburg, Germany. All other chemicals were of analytical grade and obtained from commercial sources. HA-tagged wild-type human H_1R, and H_4R, and non-tagged H_3R (full-length isoform 445), mouse H_3R, all expressed in $pcDEF_3$ were made in-house and described previously[7] $pcDEF_3$, SmBiT-PRKACA, LgBiT-PRKAR2A[8] in plasmid DNA, encoding for a NanoBit cAMP sensor were purchased from Promega.

Cell culture, transfection and preparation of cell homogenates

HEK293T cells were cultured in DMEM supplemented with 10% FBS and Penicillin (100 IU/ml) and Streptomycin (100 µg/ml) at 37° C, 5% CO_2. HEK293T cells stably expressing N-terminally tagged Nluc-hH_2R were a kind gift from the Pockes lab, University of Regensburg, Germany, and were cultured as described previously. [6]

To transiently overexpress the H_3R, 2 million HEK293T cells were seeded in a 10 cm petri dish. On the following day, cells were transfected with 2.5 µg hH_3R in $pcDEF_3$, 2.5 µg $pcDEF_3$ and 20 µg PEI per dish and cultured as described above. 48 hours post transfection, the medium was aspirated and transfected cells were collected in a 50 ml falcon, using 5 ml ice cold PBS per dish. The cell homogenate suspension was spun down at 3000 rpm, for 10 minutes at 4° C. Supernatant was aspirated, the pellet resuspended in 1 ml ice-cold PBS per dish and centrifuged again. The pellets were stored at -20° C until use. Mouse H_3R, human H_1R and H_4R were similarly overexpressed in HEK293T cells.

[^{3}H]3f binding experiments

For use in *trans*-[^{3}H]**3f** saturation binding experiments H_3R-expressing cell homogenates were resuspended in 50 mM Tris-HCl buffer (pH 7.4 at 4° C) and homogenized by sonication (Sonifier Branson). *Trans*-[^{3}H]**3f** was dissolved in 50 mM Tris-HCl (pH 7.4 at RT) containing 0.8% ethanol. A concentration range from 0.2 – 15.9 nM of *trans*-[^{3}H]**3f** was incubated in the dark with H_3R-expressing cell homogenates (7.5 µg protein in 50 µl) in a total volume of 100 µl for 2 hours at RT on a shaker at 300 rpm. The incubation was terminated by filtration of all samples on a GF/C plate (Perkin Elmer) using the FilterMate 96 harvester (Perkin Elmer). GF/C plates were dried (45 minutes at 60° C), whereafter Microscint-O (25

µl/well) was added. Radioactivity was measured after a 2-hour incubation period with Microscint-O, using the MicroBeta plate reader (Perkin Elmer). To determine non-specific binding, the binding of each *trans*-[^{3}H]**3f** concentration to H_3R was also determined after co-incubation with 10 µM clobenpropit. Specific binding was determined by subtracting the non-specific binding from the total binding.

For association experiments, *trans*-[^{3}H]**3f** (0.5 nM) was incubated with H_3R-expressing cell homogenates (7.5 µg protein in 50 µl) at RT for different time intervals, ranging from 0-60 minutes. For dissociation experiments, 0.5 nM *trans*-[^{3}H]**3f** was incubated with H_3R-expressing cell homogenates at RT for at least 20 minutes to reach equilibrium, after which dissociation was started with addition of 10 µM clobenpropit and remaining *trans*-[^{3}H]**3f** binding was determined after different time intervals (0-20 min) as described above.

To investigate the binding kinetics of [^{3}H]**3f** PSS_{cis}, *trans*-[^{3}H]**3f** (7.93 µM) was irradiated with 365 nm (10 V, 2 mW, constant mode, consistently used for this wavelength) light (96 LED array plates, LAD-1, LEDA-x, Bio Research Center Co., Ltd. Izumi, Japan) for 10 min in a black 96 well plate (Greiner), after which it was diluted to an end concentration of 25 nM. The association-dissociation experiments were performed as for *trans*-[^{3}H]**3f** but the Tris-HCl binding buffer was supplemented with 0.1 % BSA to improve solubility.

For competition binding studies ligands (10 µM to 1 pM) were incubated with [^{3}H]NAMH (2 nM), or *trans*-[^{3}H]**3f** (3.7-4.5 nM), and H_3R-expressing cell homogenates (7.5 µg protein in 50 ul) for 2 hours at 25° C.

Dynamic kinetic radioligand binding experiments with *trans*-[^{3}H]3f

After equilibration of *trans*-[^{3}H]**3f** (0.5 nM) with H_3R-expressing cell homogenates in a black 96 well plate, the mixture was irradiated with 365 nm at RT during different time intervals ranging from 0-20 minutes. The incubations were terminated by filtration and binding was measured as described above.

In another set of experiments *trans*-[^{3}H]**3f (**0.5 nM) was equilibrated with hH_3R-expressing cell homogenates and subsequently switched to PSS_{cis} by 365 nm light illumination at RT for 20 minutes. Thereafter, the mixture was irradiated with 500 nm LED (7.5 V, 1.5 mW, constant mode, consistently used for this wavelength) at RT for different time intervals ranging from 0-60 minutes. The incubations were terminated by filtration and binding was measured as described above.

Binding studies to determine receptor selectivity of 3f

Radioligand competition binding experiments to investigate receptor selectively of **3f** were performed as described before[2,3] with slightly altered incubation times (2 hours), using cell homogenates expressing either human H_1R, H_3R or H_4R and the subtype specific radioligands [^{3}H]mepyramine (1.9 – 2.4 nM), [^{3}H]N-alpha-methyl-histamine ([^{3}H]NAMH) (1.4 – 1.7 nM) or [^{3}H]histamine (4.0 – 4.8 nM), respectively. H_2R selectivity was assessed using a NanoBRET binding assay as published[4] with minor modifications. The assay was performed with HEK293T cells stably expressing Nluc-H_2R. Cells were incubated with ligands and the fluorescent H_2R tracer UR-KAT478 for 2 hours at 25 °C. Thereafter, Nano-Glo® (Promega, catalog number N2011) (3.2 µL/ml), the substrate of Nluc, was added and BRET signal generated due to the close proximity of Nluc and Nano-Glo® and UR-KAT478 was measured using CLARIOstar (BMG, Germany), separating luminescence bands at 470 nm and 640 nm (80 nm bandwitdh each).

NanoBiT– protein kinase A (PKA) assays

HEK293T cells were transfected in suspension with 0.3 µg of SmBiT-PRKACA, 0.3 µg LgBiT-PRKAR2A[6] and 1.4 µg hH_3R per million cells in a 1:6 DNA to linear PEI ratio and immediately seeded in white 96-well plates (Greiner) in a density of 60 000 cells per well. Post transfection (24 hours), DMEM was exchanged for 60 µL HBSS, after which 10 µL antagonists (10 µM – 1 pM) were added, and subsequently 10 µL of 10 nM immethridine and 10 µL of 10 µM forskolin were added. Thereafter, 10 µL Nano-Glo® in an end dilution of 3.2 µL/mL was added and luminescence was measured over time in the CLARIOstar (BMG, Germany) at 470 nm (80 nm bandwith).

To dynamically photoswitch **3f** during the assay, the plate was taken out of CLARIOstar following a kinetic cycle of 20 min. The plate was covered with a transparent lid and irradiated from the top with 365 nm light for 10 min at RT followed by an endpoint measurement in the CLARIOstar. Next, the plate was irradiated as described above with 500 nm light for 10 min at RT followed by measurement in the CLARIOstar. This process was repeated as described for an additional irradiation-measurement cycle.

Data analysis

GraphPad Prism 10 was used for all data analysis and visualization. Functional data were fitted using the non-linear regression “three parameter dose-response curve” to obtain pEC_{50} and pIC_{50} values. Saturation binding data were analyzed using the “one-site binding”

model to obtain K_d values. Competition binding data was fitted using a "one-site binding" model to obtain pIC_{50} values, which were converted into pK_i values using the Cheng-Prusoff equation.[9]

Association and dissociation binding data were analyzed using a "one-site association model for one radioligand concentration" and "one-phase exponential decay" model, respectively.

Experimental procedures – Computational chemistry

Preparation of receptor structure

The crystal structure of inactive histamine H_3R bound to PF-03654746 (PDB: 7F61)[4] was used as the starting point of our molecular modeling studies. Using MODELER[10], ICL3 was remodeled and the S121$^{3.39}$K mutation in the sodium binding pocket was reverted to the wild-type. To allow docking of the photoswitchable compounds, the rotamer of Y189^{ECL2} within the EBP was adjusted to enlarge the pocket. Y189^{ECL2} thus adopts a rotamer similar to that observed in the cryo-EM structures of H_3R in active state (PDB: 8YUU and 8YUV).[11] Additionally, a sodium ion was placed in the sodium binding site based on the sodium-bound β_1-adrenoreceptor crystal structure (PDB: 4BVN).[12]

Molecular docking

Molecular docking was performed using ICM-Pro (v3.9-3a) molecular modeling and drug discovery suite (Molsoft).[13] During docking, ligands were treated as flexible, with binding poses and internal torsions sampled using the biased probability Monte Carlo (BPMC) procedure.[13] This process included local energy minimization after each random move. Comprehensive sampling of the ligand conformational space was performed with effort parameter was set to 30 with 30 conformations scored with the ICM VLS score. To not interconvert isomers during the docking process, the option “relax covalent geometry” was not selected. Binding poses with the lowest score and interaction with D114$^{3.32}$ of each compound and isomer were selected for further analysis. The selected binding poses were further optimized in a postprocessing step using BPMC sampling, which optimized the ligand conformation and side-chain residues located within 5 Å of the ligand.

Molecular dynamics simulation

The H_3R complexes obtained from docking were prepared for molecular dynamics simulations with the CHARMM-GUI web server.[14] As terminal group patching, the N-terminus was acetylated, the C-terminus was methylamidated. The disulfide bonds between residues C107$^{3.25}$-188^{ECL2} and C384$^{6.61}$-C388^{ECL3} were checked for proper linkage. The complexes were embedded in a box of approximately 80x80x120 Å containing a POPC membrane bilayer, TIP3 water molecules, and a 150 mM concentration of NaCl. Ligands were parameterized using CGenFF.[15] Parameters of the azobond dihedral with a minimum of 180° for *cis* isomers were adjusted from 63.60 to 75.73 kJ/mol and azobond dihedrals with a minimum of 0° for *cis*

isomers were adjusted from 2.09 to 1.26 kJ/mol in line with parameters reported by Klaja et al.[16] The CHARMM36m force field[17] was applied for the rest of the system.

All simulations were performed using GROMACS 2023.1.[18] The system was first minimized by a 5000-step steepest descent minimization using periodic boundary conditions. Subsequently, the system was equilibrated in multiple steps, following the default CHARMM equilibration protocol with only the simulation time doubled. After equilibration, a 1000 ns production run in the NPT ensemble was performed using a time step integration of 4 fs with hydrogen mass repartitioning. LINCS constraints were applied to the hydrogen covalent bonds. The electrostatic interactions were calculated with the particle-mesh Ewald (PME) algorithm with a cutoff of 1.2 nm. The v-rescale algorithm was used to maintain a temperature of 310.15 K and the pressure was kept at 1 bar by semi-isotropic coupling to c-rescale barostat (τp = 5.0 ps, compressibility = $4.5*10^{-5}$ bar). Atomic positions were saved every 100 ps.

Frequencies of ligand-protein and water-mediated interactions were calculated with ProLIF[19] and the use of in-house Python scripts. To ensure proper equilibration, the initial 500 ns of each simulation were excluded from the analysis. Representative binding modes from MD simulation of the *cis* and *trans* isomers of **3f** were obtained by using the Gromacs clustering command (*gmx cluster*). The first 500 ns of the 1000 ns simulations was discarded and the remaining 500 ns of each of the four independent simulations were concatenated. Gromacs clustering of the H_3R pocket residues (residue IDs: 27, 91, 94, 95, 98, 110, 111, 114, 115, 118, 187, 188, 189, 190, 193, 374, 378, 391, 394, 395, 398, 401, 402) was performed and the highest populated cluster was deemed as representative for the average binding mode of the ligand.

Experimental procedures – Photochemistry

UV−Vis spectra were recorded using a Thermo-scientific Evolution 201 PC spectrophotometer equipped with a thermostated cell holder set at 20 °C. Fits of UV−vis spectroscopy data were generated using Prism 10.1.0. Illumination for almost all photochemical experiments was executed using a Sutter instruments Lambda LS with a 300 W full-spectrum lamp connected to a Sutter instruments Lambda 10-3 optical filter changer equipped with 360 ± 10 nm, 434 ± 9 nm 494 ± 9 nm, or a Lambda 721 optical beam combiner system equipped with 365 ± 11 nm and 506 ± 14 nm filter. The light intensity used for the lambda 10-3 optical filter is 0.93 mW/mm^2 using the 360 ± 20 nm filter, 0.79 mW/mm^2 for the 434 ± 9 nm filter, 0.77 mW/mm^2 for the 494 ± 9 nm filter as measured using a Thorlabs PM16−401 power meter. The light intensity used for the lambda 721 optical beam combiner is 200 mW using the 365 ± 11 nm filter and 60 mW for the 506 ± 12 nm filter. For photochemical analyses, illuminations were performed in Hellma Suprasil quartz 114QS cuvettes. For the dynamic illuminations, a 4-way cuvette holder (Avantes) was used. The left outlet of the holder is connected to an Avantes AvaLight-DH-S-BAL Deuterium lamp. The right outlet is connected through a fiber to an AvaSpec UV/Vis spectrometer. The lower outlet is connected to a Lambda 721 optical beam combiner system equipped with 365 ± 11 nm and 506 ± 14 nm filter illumination means. The upper outlet is covered with a black cap. The temperature of the solution in the cuvette (20 °C) is controlled by Q-blue Wireless Temperature Control software. Thermal relaxation experiments and Arrhenius extrapolations were performed according to Priimagi *et al*[20], using a compound concentration of 25 µM in HBSS +1% DMSO and temperatures of 50, 60, and 70 °C. Illuminations for pharmacological experiments were performed in cylindrical clear glass vials with a volume of 4.5 mL. The typical distance between light source and vial or cuvette was 2 cm.

Experimental procedures - Synthesis

All chemicals and solvents were obtained from commercial suppliers (primarily Sigma-Aldrich, BLD pharma and Combi-Blocks) and used without purification. DCM, DMF, THF and Et_2O were dried by passing through a PureSolv solvent purification system. All reactions with photoresponsive compounds were carried out in the dark or under red light. IUPAC names were adapted from ChemDraw Professional 21.0 (PerkinElmer). Reactions were monitored by thin layer chromatography (Merck Silicagel 60 F254) by visualization under 254 nm lamp. Flash column chromatography was performed with Screening Devices 40-63 µm (Biotage) or GraceResolv (Büchi) cartridges on Isolera One with UV-Vis detection (Biotage). Nuclear magnetic resonance (NMR) spectra were determined with a Bruker Avance II 500 MHz or a Bruker Avance III HD 600 MHz spectrometer. Chemical shifts are reported in parts per million (ppm) against the reference compound using the signal of the residual non-deuterated solvent ($CDCl_3$ δ = 7.26 ppm (^1H), δ = 77.16 ppm (^{13}C); DMSO-d_6 δ = 2.50 ppm (^1H), δ = 39.52 ppm (^{13}C)). NMR spectra were processed using MestReNova 14.0 software. The peak multiplicities are defined as follows: s, singlet; d, doublet; t, triplet; q, quartet; dd, doublet of doublets; ddd, doublet of doublets of doublets; dt, doublet of triplets; dq, doublet of quartets; td, triplet of doublets; tt, triplet of triplets; qd, quartet of doublets; p, pentet; dp, doublet of pentets; br, broad signal; m, multiplet. For NMR listings, in addition to specific instructions that are given by the journal in the guidelines for authors the following additional procedures were used: 1) Multiplicity is not solely reported based on peak shapes, but also distinguishes the coupling to all non-equivalent protons that have similar J values; 2) If additional smaller couplings are observed but are too small for accurate quantitation because the precision is smaller than the digital resolution, a symbol $^{\Delta}$ will be used; 3) The notation 'm' is used in case of obscured accurate interpretation as a result of (i) overlapping signals for different protons, or (ii) a result of overlapping signal lines within the same proton signal; 4) For any rotamers or diastereomers, signals will be listed separately; 5) NMR signals that could only be detected with HSQC analysis are denoted with a $^{\#}$ symbol; 6) NMR signals that could only be detected with HMBC analysis are denoted with a * symbol; 7) If one or more signals remain undetected after extensive 1D and 2D NMR analyses, this will be mentioned. 8) Signals for exchangeable proton atoms (such as NH and OH groups) are only listed if clearly visible (excluding e.g. the use of D_2O or CD_3OD) and if confirmed by a D_2O shake and/or HSQC. Purity determination was performed with Liquid Chromatography using a Shimadzu LC-20AD liquid chromatography pump system with a Shimadzu SPDM20A photodiode array detector and MS

detection with a Shimadzu LCMS-2010EV mass spectrometer operating in both positive and negative ionization mode. A Waters XBridge C18 column 5 µm 4.6 x 50 mm was used at 40 °C. The mobile phase used was a mixture of A = Water + 0.1% HCO_2H and B = acetonitrile (MeCN) + 0.1% HCO_2H. The eluent program 'acidic mode' used is as follows: flow rate: 1.0 mL/min, start 95% A in a linear gradient to 10% A over 4.5 min, hold 1.5 min at 10% A, in 0.5 min in a linear gradient to 95% A, hold 1.5 min at 95% A, total runtime: 8.0 min. Compound purities were calculated as the percentage peak area of the analysed compound by UV detection at 254 nm. Unless mentioned otherwise, all compounds have a LC purity of >95 %. High-resolution mass spectra (HRMS) were recorded on a Bruker micrOTOF mass spectrometer using ESI in positive ion mode (HRMS).

General procedure A

To a solution of 3-(2-(4-hydroxyphenyl)hydrazineylidene)pentane-2,4-dione (**5**) in EtOH (0.12 M) was added the respective hydrazine (1.0 eq) and Et_3N (2.0 eq). The reaction mixture was heated at reflux for 17 h. The reaction mixture was cooled to rt and concentrated under reduced pressure. Where indicated, the obtained crude product was purified by flash column chromatography to give phenols **6a–d,f**.

General procedure B

To a solution of phenol **6** (1.5 eq) in DMF (0.25 M) was added NaH (60% dispersion in mineral oil, 2.0 eq). The reaction mixture was stirred for 30 min at rt. Subsequently, tosylate **7**[2] (1.0 eq) was added. The reaction mixture was heated at 75 °C for 17 h. The reaction mixture was cooled to rt, poured in ice-water and extracted thrice with EtOAc. The combined organic layers were washed with brine, dried over Na_2SO_4 and concentrated under reduced pressure. The crude product was purified using reverse phase (MPLC). The desired fractions were combined, made basic using satd. aq. $NaHCO_3$ and extracted using EtOAc. The combined organic layers were washed with brine, dried over Na_2SO_4 and concentrated *in vacuo*. The products were lyophilised to give compounds **3a–d,f.**

3-(2-(4-hydroxyphenyl)hydrazineylidene)pentane-2,4-dione (5)

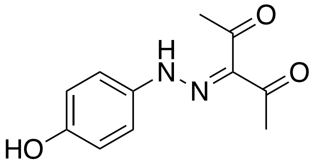

A solution of 4-aminophenol (**4**, 5.0 g, 46 mmol, 1.0 eq) in AcOH (70 mL) and conc. aq. HCl (11 mL) was cooled to 0 °C. A solution of $NaNO_2$ (3.8 g, 55 mmol, 1.2 eq) in water (10 mL) was added

drop-wise. After stirring for 45 min at 0 °C, the resulting solution containing the diazonium salt was added drop-wise to another flask cooled to 0 °C containing a suspension of pentane-2,4-dione (6.1 mL, 60 mmol, 1.3 eq) and NaOAc (11.3 g, 137 mmol, 3.0 eq) in EtOH (46 mL) and water (28 mL). The mixture was stirred for 1 h at 0 °C. The resulting brown precipitate was collected via vacuum filtration. After washing with water, water/EtOH (1:1) and *c*Hex the obtained solid was dried under vacuum affording the product as a dark yellow solid (5.8 g, 58% yield). **^{1}H NMR** (600 MHz, DMSO-d$_6$) δ 14.49 (s, 1H), 9.63 (s, 1H), 7.44 (d, J = 8.9 Hz, 2H), 6.83 (d, J = 8.9 Hz, 2H), 2.46 (s, 3H), 2.38 (s, 3H). **^{13}C NMR** (151 MHz, DMSO-d$_6$) δ 196.0, 195.9, 155.9, 133.7, 132.1, 118.1, 116.1, 31.0, 26.4. **LCMS** (acidic)**:** t$_r$: 3.49 min, purity: 96% (254 nm).

(*E*)-4-((1,3,5-trimethyl-1*H*-pyrazol-4-yl)diazenyl)phenol (**6a**)

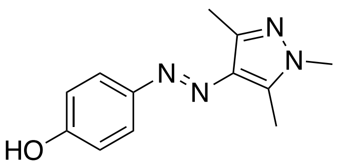

General procedure A was followed, using dione **5** (400 mg, 1.8 mmol), MeHNNH$_2$ (143 µL, 2.7 mmol) and EtOH (15 mL). Concentration *in vacuo* gave compound **3a** (411 mg, 98% yield). **^{1}H NMR** (600 MHz, DMSO-d$_6$) δ 9.93 (s, 1H), 7.61 (d, *J* = 8.7 Hz, 2H), 6.87 (d, *J* = 8.9 Hz, 2H), 3.71 (s, 3H), 2.51 (s, 3H), 2.34 (s, 3H). **^{13}C NMR** (151 MHz, DMSO-d$_6$) δ 159.1, 146.1, 139.9, 138.1, 134.0, 123.1, 115.6, 35.9, 13.7, 9.4. **LCMS** (acidic)**:** t$_r$: 3.67 min, purity: 99% (254 nm), λ_{max}: 345 nm, $[M+H]^+$ m/z calc. 231.12, found 231.10.

(*E*)-4-((1-cyclohexyl-3,5-dimethyl-1*H*-pyrazol-4-yl)diazenyl)phenol (**6b**)

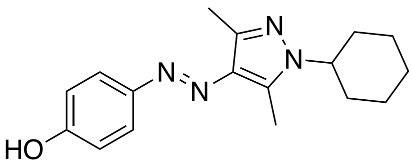

General procedure A was followed, using dione **5** (500 mg, 2.3 mmol), cyclohexylhydrazine hydrochloride (342 mg, 2.3 mmol), Et$_3$N (0.63 mL, 4.5 mmol) and EtOH (19 mL). Purification by column chromatography (0 -> 50% 90:5:5 EtOAc:MeOH:TEA in cHex) gave the title compound as a yellow solid (497 mg, 73% yield). **^{1}H NMR** (500 MHz, CDCl$_3$) δ 7.71 (d, J = 9.0 Hz, 2H), 6.90 (d, J = 8.7 Hz, 2H), 6.76 (s, 1H), 4.00 (dddd, J = 11.7, 11.7, 3.8, 3.8 Hz, 1H), 2.58 (s, 3H), 2.50 (s, 3H), 2.05 – 1.95 (m, 2H), 1.95 – 1.85 (m, 4H), 1.74 – 1.71 (m, 1H), 1.44 – 1.35 (m, 2H), 1.29 (dddd, J = 11.9, 11.9, 2.5, 2.5 Hz, 1H). **^{13}C NMR** (126 MHz, CDCl$_3$) δ 157.6, 148.0, 142.0, 137.3, 134.7, 123.6, 115.8,

57.9, 32.6, 25.9, 25.3, 14.0, 9.8. **LCMS** (acidic)**:** t_r: 4.95 min, purity: 99% (254 nm), λ_{max}: 349 nm, $[M+H]^+$ m/z calc. 299.19, found 299.15.

(*E*)-4-((3,5-dimethyl-1-(tetrahydro-2*H*-pyran-4-yl)-1*H*-pyrazol-4-yl)diazenyl)phenol (6c)

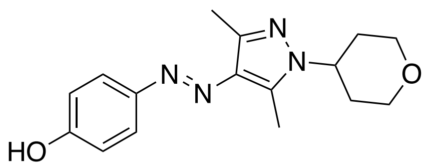

General procedure A was followed, using dione **5** (300 mg, 1.4 mmol), (tetrahydro-2H-pyran-4-yl)hydrazine hydrochloride (311 mg, 1.4 mmol), Et_3N (0.38 mL, 2.8 mmol) and EtOH (11 mL). Purification by column chromatography (0 -> 50% 90:5:5 EtOAc:MeOH:TEA in cHex) gave the title compound as a yellow solid (170 mg, 42% yield). **^{1}H NMR** (600 MHz, $CDCl_3$) δ 7.72 (d, 2H), 6.90 (d, 2H), 5.69 (s, 1H), 4.24 (dddd, *J* = 11.6, 11.6, 4.1, 4.1 Hz, 1H), 4.15 (dd$^{\Delta}$, *J* = 11.4, 4.5 Hz, 2H), 3.55 (ddd, *J* = 12.2, 12.1, 1.9 Hz, 2H), 2.61 (s, 3H), 2.50 (s, 3H), 2.37 (dddd, *J* = 12.3, 12.0, 12.0, 4.6 Hz, 2H), 1.84 (ddd$^{\Delta}$, *J* = 13.0, 4.3, 2.0 Hz, 2H). **^{13}C NMR** (151 MHz, $CDCl_3$) δ 157.2, 148.2, 142.2, 137.4, 135.0, 123.7, 115.7, 67.3, 54.9, 32.5, 14.2, 9.8. **LCMS** (acidic)**:** t_r: 3.84 min, purity: 96% (254 nm), λ_{max}: 345 nm, $[M+H]^+$ m/z calc. 301.17, found 301.15.

***tert*-butyl (*E*)-4-(4-((4-hydroxyphenyl)diazenyl)-3,5-dimethyl-1H-pyrazol-1-yl)piperidine-1-carboxylate (6d)**

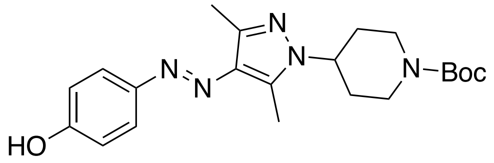

General procedure A was followed, using dione **5** (350 mg, 1.6 mmol), *tert-butyl* 4-hydrazineylpiperidine-1-carboxylate oxalate (485 mg, 1.6 mmol), Et_3N (0.44 mL, 3.2 mmol) and EtOH (11 mL). Purification by column chromatography (30 -> 70% EtOAc in cHex) and additional co-evaporation of the concentrated desired fractions with Et_2O, gave the title compound as a fluffy yellow solid (363 mg, 57% yield). **^{1}H NMR** (500 MHz, $CDCl_3$) δ 7.72 (d, *J* = 8.8 Hz, 2H), 6.90 (d, *J* = 8.8 Hz, 2H), 4.44 – 4.20 (m, 2H), 4.15 (dddd, *J* = 11.6, 11.6, 4.1, 4.1 Hz, 1H), 2.96 – 2.77 (m, 2H), 2.59 (s, 3H), 2.48 (s, 3H), 2.25 – 2.11 (m, 2H), 1.91 – 1.83 (m, 2H), 1.48 (s, 9H). **^{13}C NMR** (126 MHz, $CDCl_3$) δ 157.3, 154.6, 148.2, 142.2, 137.4, 135.0, 123.7, 115.7, 80.1, 77.4, 77.2, 76.9, 55.9, 42.9$^{\#}$, 31.6, 28.6, 14.2, 9.8. **LCMS** (acidic)**:** t_r: 4.83, purity: 91%, λ_{max}: 345 nm, $[M+H]^+$ m/z calc. 400.23, found 400.25.

(*E*)-4-((3,5-dimethyl-1-(1-methylpiperidin-4-yl)-1*H*-pyrazol-4-yl)diazenyl)phenol (6f)

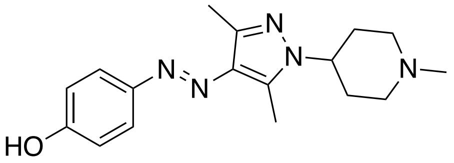

General procedure A was followed, using dione **5** (500 mg, 2.3 mmol), 4-hydrazineyl-1-methylpiperidine dihydrochloride (459 mg, 2.3 mmol), Et_3N (0.63 mL, 4.6 mmol) and EtOH (19 mL). Purification by column chromatography (50 -> 100% 90:5:5 EtOAc:MeOH:TEA in cHex) gave the title compound as a yellow solid (551 mg, 77% yield). **^{1}H NMR** (500 MHz, DMSO-d_6) δ 9.91 (s, 1H), 7.61 (d, *J* = 8.8 Hz, 2H), 6.86 (d, *J* = 8.9 Hz, 2H), 4.12 (dddd, *J* = 10.9, 5.1 Hz, 1H), 2.92 – 2.82 (m, 2H), 2.55 (s, 3H), 2.35 (s, 3H), 2.21 (s, 3H), 2.12 – 2.00 (m, 4H), 1.84 – 1.75 (m, 2H). **^{13}C NMR** (126 MHz, DMSO-d_6) δ 159.0, 146.1, 139.7, 137.8, 133.9, 123.1, 115.6, 54.5, 54.3, 45.9, 31.1, 14.2, 9.0. **LCMS** (acidic)**:** t_r: 2.69 min, purity: >99% (254 nm), λ_{max}: 345 nm, $[M+H]^+$ m/z calc. 314.20, found 314.20.

1-((1*r*,3*r*)-3-(4-((*E*)-(1,3,5-trimethyl-1*H*-pyrazol-4-yl)diazenyl)phenoxy)cyclobutyl)piperidine fumarate (3a)

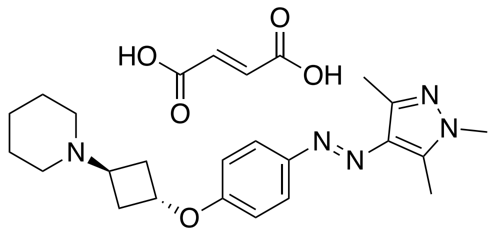

General procedure B was followed, using phenol **6a** (179 mg, 0.78 mmol), NaH (31 mg, 0.78 mmol), and tosylate **7** (105 mg, 0.39 mmol). The crude product was purified by reverse phase column chromatography (5 -> 95% MeCN + 0.1% HCO_2H in H_2O + 0.1% HCO_2H). Desired fractions were combined, made basic using aq. satd. $NaHCO_3$ and extracted with EtOAc thrice. The combined organic layers were dried over Na_2SO_4, filtered and concentrated to give the free base as a yellow oil. The free base was converted to the fumarate salt by recrystallization from IPA to give the title compound as yellow crystals (34 mg, 24%).

^{1}H NMR (600 MHz, CD_3OD) δ 7.79 – 7.70 (m, 2H), 6.96 – 6.88 (m, 2H), 6.71 (s, 2H), 4.93 (ddd, *J* = 7.0, 4.7, 2.1 Hz, 1H), 3.92 – 3.84 (m, 1H), 3.77 (s, 3H), 2.85 – 2.73 (m, 2H), 2.62 – 2.52 (m, 5H), 2.43 (s, 3H), 1.99 – 1.55 (m, 6H). **^{13}C NMR** (151 MHz, CD_3OD) δ 171.4, 159.7, 149.5, 142.8, 140.4, 136.3, 135.7, 124.4, 116.3, 68.9, 58.6, 51.7, 36.0, 33.3, 24.2, 22.9, 13.8, 9.7. **LCMS** (acidic)**:** t_r: 3.17 min, purity: 99% (254 nm), λ_{max}: 344 nm, $[M+H]^+$ m/z calc. 368.24, found 368.25. **HRMS**: $[M+H]^+$ calc. for $C_{21}H_{30}N_5O$: 368.2445, found 368.2441.

1-((1*r*,3*r*)-3-(4-((*E*)-(1-cyclohexyl-3,5-dimethyl-1*H*-pyrazol-4-yl)diazenyl)phenoxy)cyclobutyl) piperidine fumarate (**3b**)

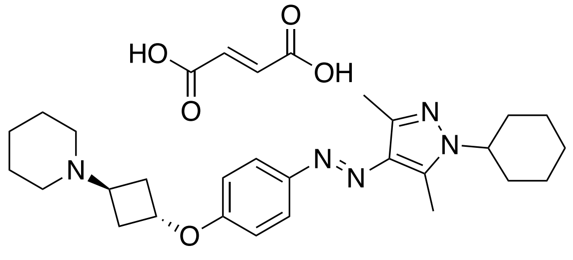

General procedure B was followed, using phenol **6b** (159 mg, 0.53 mmol), NaH (28 mg, 0.71 mmol), and tosylate **7** (110 mg, 0.39 mmol). The crude product was purified by reverse phase column chromatography (5 -> 95% MeCN + 0.1% HCO_2H in H_2O + 0.1% HCO_2H). Desired fractions were combined, made basic using aq. satd. $NaHCO_3$ and extracted with EtOAc thrice. The combined organic layers were dried over Na_2SO_4, filtered and concentrated to give the free base as a yellow oil. The free base was converted to a fumarate salt by recrystallization from IPA to give the title compound as yellow crystals (80 mg, 52%).

^{1}H NMR (500 MHz, CD_3OD) δ 7.74 (d, *J* = 9.5 Hz, 2H), 6.93 (d, *J* = 9.5 Hz, 2H), 6.71 (s, 2H), 4.94 (tt, *J* = 6.9, 2.2 Hz, 1H), 4.21 – 4.10 (m, 1H), 3.92 (tt, *J* = 8.0, 8.0 Hz, 1H), 3.18 – 3.02 (m, 2H), 2.80 (ddd, *J* = 11.2, 9.1, 5.2 Hz, 2H), 2.63 – 2.55 (m, 5H), 2.44 (s, 3H), 1.97 – 1.79 (m, 10H), 1.79 – 1.60 (m, 3H), 1.55 – 1.45 (m, 2H), 1.32 (dddd, *J* = 16.6, 13.1, 8.1, 4.7 Hz, 2H). **^{13}C NMR** (126 MHz, CD_3OD) δ 169.6, 158.2, 148.2, 141.2, 138.2, 134.7, 134.1, 123.0, 114.9, 67.5, 57.3, 57.3, 50.3, 48.1, 47.9, 47.8, 47.6, 47.4, 47.3, 47.1, 32.1, 31.9, 25.2, 25.0, 22.8, 21.4, 12.7, 8.1. **LCMS** (acidic)**:** t_r: 3.99 min, purity: 98% (254 nm), λ_{max}: 349 nm, $[M+H]^+$ m/z calc. 436.31, found 436.35. **HRMS**: $[M+H]^+$ calc. for $C_{26}H_{38}N_5O$: 436.3071, found 436.3064.

1-((1*r*,3*r*)-3-(4-((*E*)-(3,5-dimethyl-1-(tetrahydro-2*H*-pyran-4-yl)-1*H*-pyrazol-4-yl)diazenyl) phenoxy)cyclobutyl)piperidine fumarate (**3c**)

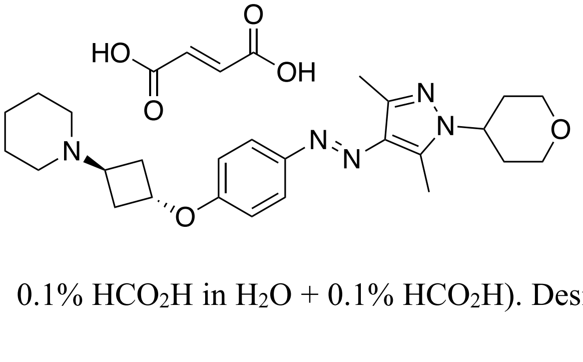

General procedure B was followed, using phenol **6c** (152 mg, 0.51 mmol), NaH (27 mg, 0.68 mmol), and tosylate **7** (105 mg, 0.34 mmol). The crude product was purified by reverse phase column chromatography (5 -> 95% MeCN + 0.1% HCO_2H in H_2O + 0.1% HCO_2H). Desired fractions were combined, basified using satd.

aq. $NaHCO_3$ and extracted with EtOAc thrice. The combined organic layers were dried over Na_2SO_4, filtered and concentrated to give the free base as a yellow oil. The free base was converted to a fumarate salt by recrystallization from IPA to give the title compound as yellow crystals (43 mg, 29%).

^{1}H NMR (500 MHz, CD_3OD) δ 7.65 (d, *J* = 9.0 Hz, 2H), 6.83 (d, *J* = 9.0 Hz, 2H), 6.61 (s, 2H), 4.84 (tt, *J* = 7.0, 2.2 Hz, 1H), 4.35 (tt, *J* = 11.6, 4.1 Hz, 1H), 3.98 (dd, *J* = 11.4, 4.7 Hz, 2H), 3.88 – 3.79 (m, 1H), 3.51 (ddd, *J* = 12.1, 2.0, 2.0 Hz, 2H), 3.18 – 2.79 (m, 4H), 2.76 – 2.68 (m, 2H), 2.57 – 2.45 (m, 5H), 2.35 (s, 3H), 2.20 – 2.08 (m, 2H), 1.85 – 1.70 (m, 6H), 1.65 – 1.48 (m, 2H). **^{13}C NMR** (126 MHz, CD_3OD) δ 169.3, 158.3, 148.2, 141.5, 138.5, 134.7, 134.3, 123.0, 114.9, 67.5, 66.6, 57.3, 54.2, 50.3, 32.0, 31.9, 22.7, 21.4, 12.7, 8.0. **LCMS** (acidic)**:** t_r: 3.29 min, purity: 96% (254 nm), λ_{max}: 349 nm, $[M+H]^+$ m/z calc. 438.29, found 438.35. **HRMS**: $[M+H]^+$ calc. for $C_{25}H_{36}N_5O_2$: 438.2864, found 436.3064.

tert-butyl 4-(3,5-dimethyl-4-((_E_)-(4-((1_r_,3_r_)-3-(piperidin-1-yl)cyclobutoxy)phenyl)diazenyl)-1_H_-pyrazol-1-yl)piperidine-1-carboxylate (3d)

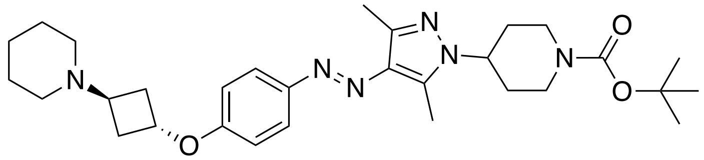

General procedure B was followed, using phenol **6d** (152 mg, 0.49 mmol), NaH (26 mg, 0.65 mmol), and tosylate **7** (100 mg, 0.32 mmol). The crude compound was purified by column chromatography (20 -> 70% 90:5:5 EtOAc:MeOH:TEA in cHex) to give the Boc-protected intermediate **3d** as a yellow solid (72 mg, 42%). **^{1}H NMR** (600 MHz, CD_3OD) δ 7.72 (d, *J* = 8.9 Hz, 2H), 6.88 (d, *J* = 8.9 Hz, 2H), 4.84 – 4.81 (m, 1H), 4.38 (tt, *J* = 11.6, 4.1 Hz, 1H), 4.23 (d, *J* = 13.5 Hz, 2H), 3.20 – 3.09 (m, 1H), 3.04 – 2.82 (m, 2H), 2.61 (s, 3H), 2.58 – 2.19 (m, 11H), 2.04 (ddd, *J* = 12.6, 12.4, 4.3 Hz, 2H), 1.95 – 1.83 (m, 2H), 1.72 – 1.61 (m, 4H), 1.60 – 1.42 (m, 11H). **^{13}C NMR** (151 MHz, CD_3OD) δ 160.3, 156.3, 149.2, 142.9, 139.8, 135.6, 135.6, 124.4, 116.2, 81.3, 70.3, 58.7, 56.4, 51.9, 34.2, 32.5, 28.7, 25.9, 24.8, 14.1, 9.4. **LCMS** (acidic)**:** t_r: 4.01 min, purity: 95% (254 nm), λ_{max}: 345 nm, $[M+H]^+$ m/z calc. 537.35, found 537.45.

1-((1_r_,3_r_)-3-(4-((_E_)-(3,5-dimethyl-1-(piperidin-4-yl)-1_H_-pyrazol-4-yl)diazenyl)phenoxy) cyclobutyl)piperidine (3e)

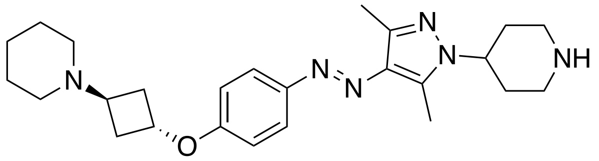

Compound **3d** (72 mg, 0.13 mmol) was dissolved in dioxane (1 mL) and 4M HCl in dioxane (1 mL) was added. The reaction mixture was stirred overnight at rt. The reaction mixture was concentrated to give a yellow solid, which was mixed with satd. aq. $NaHCO_3$. Extraction was performed with EtOAc thrice. The combined organic layers were washed with water and brine, dried over Na_2SO_4, filtered and concentrated. The crude product was purified by reverse phase column chromatography (5 -> 95% MeCN + 0.1% HCO_2H in H_2O + 0.1% HCO_2H). Desired fractions were combined, made basic using aq. sat. $NaHCO_3$ and extracted with EtOAc thrice. The combined organic layers were dried over Na_2SO_4, filtered and concentrated to give the free base as a yellow solid (41 mg, 70%). **^{1}H NMR** (600 MHz, CD_3OD) δ 7.73 (d, *J* = 9.2 Hz, 2H), 6.89 (d, *J* = 8.9 Hz, 2H), 4.83 (tt, *J* = 4.1, 2.7 Hz, 2H), 4.34 (tt, *J* = 11.7, 4.0 Hz, 1H), 3.26 – 3.17 (m, 2H), 3.04 (tt, *J* = 7.5, 7.5 Hz, 1H), 2.80 (dd$^\Delta$, *J* = 13.0, 2.6 Hz, 2H), 2.62 (s, 3H), 2.48 – 2.40 (m, 6H), 2.53 – 2.22 (m, 11H), 2.27 (d, *J* = 0.8 Hz, 3H), 2.10 (ddd, *J* = 12.7, 12.6, 4.2 Hz, 2H), 1.95 – 1.88 (m, 2H), 1.64 (tt, *J* = 5.7, 5.7 Hz, 4H), 1.56 – 1.44 (m, 2H). **^{13}C NMR** (151 MHz, CD_3OD) δ 160.3, 149.2, 142.7, 139.7, 135.6, 124.3, 116.2, 70.5, 58.7, 56.5, 52.0, 46.0, 34.4, 33.2, 26.1, 25.1, 14.2, 9.4. **LCMS** (acidic)**:** t_r: 2.45 min, purity: 97% (254 nm), λ_{max}: 338 nm, $[M+H]^+$ m/z calc. 437.30, found 437.30. **HRMS**: $[M+H]^+$ calc. for $C_{25}H_{37}N_6O_2$: 437.3023, found 437.3022.

4-(3,5-dimethyl-4-((*E*)-(4-((1*r*,3*r*)-3-(piperidin-1-yl)cyclobutoxy)phenyl)diazenyl)-1*H*-pyrazol-1-yl)-1-methylpiperidine (3f)

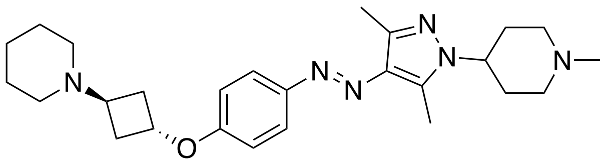

General procedure B was followed, using phenol **6f** (152 mg, 0.49 mmol), NaH (26 mg, 0.65 mmol), and tosylate **7** (100 mg, 0.32 mmol). The crude product was purified by reverse phase column chromatography (5 -> 95% MeCN + 0.1% HCO_2H in H_2O + 0.1% HCO_2H). Desired fractions were combined, made basic using aq. satd. $NaHCO_3$ and extracted with EtOAc thrice. The combined organic layers were dried over Na_2SO_4, filtered and concentrated to give the free base as a yellow solid (31 mg, 21%). **^{1}H NMR** (600 MHz, CD_3OD) δ 7.72 (d, *J* = 8.9 Hz, 2H), 6.89 (d, *J* = 8.9 Hz, 2H), 4.83 (tt, *J* = 6.8, 3.4 Hz, 1H), 4.20 (ddd$^\Delta$, *J* = 11.1, 7.8, 4.2 Hz, 1H), 3.09 – 2.99 (m, 3H), 2.61 (s, 3H), 2.55 – 2.14 (m, 18H), 1.97 – 1.82 (m, 2H), 1.63 (tt, *J* = 5.7, 5.7 Hz, 4H), 1.57 – 1.40 (m,

2H). **^{13}C NMR** (151 MHz, CD_3OD) δ 160.3, 149.2, 142.6, 139.8, 135.8, 124.3, 116.2, 70.5, 58.7, 56.3, 55.7, 52.0, 46.1, 34.4, 32.2, 26.1, 25.1, 14.2, 9.4. **LCMS** (acidic)**:** t_r: 2.47 min, purity: 99% (254 nm), λ_{max}: 343 nm, $[M+H]^+$ m/z calc. 451.32, found 451.30. **HRMS**: $[M+H]^+$ calc. for $C_{26}H_{39}N_6O_2$: 451.3180, found 451.3184.

Radiolabeling of 3f with [^{3}H]methyl nosylate

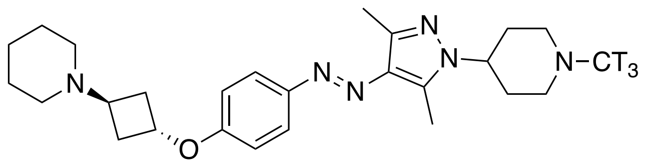

Compound **3e** (0.22 mg, 0.55 µmol) was dissolved in 117 µL of [^{3}H]methyl nosylate (Perkin Elmer, 827 MBq/mL in MeCN). The reaction mixture was heated for 15 min at 70 °C. Next, the reaction mixture was allowed to cool to rt, diluted with 2 mL eluent and injected onto a HPLC system (Jasco PU-2080 Pump, Jasco UV-2075 UV detector, Luna C18, 10x250 mm, 100 Å, 10µm, 60% acetonitrile, 40% water, 0.2% DIPEA, 5 ml/min, 254 nm) for purification. The product eluted between 38 and 44 min and was collected in a solution of 80 mL water. The total mixture was purged over a Sep-Pak tC18 (Waters, Milford, USA) which was pre-washed with 10 mL of EtOH and 10 mL of water, successively. After trapping of [^{3}H]**3f**, the Sep-Pak was washed with 20 mL of water and [^{3}H]**3f** was obtained with elution of the Sep-Pak with 2 mL of EtOH. The concentration of [^{3}H]**3f** in EtOH was determined using beta counting (Hidex 300 SL, Turku, Finland) and found to be 22.3 MBq/mL. The product was analyzed with HPLC (Jasco PU-2080 Pump, Jasco UV-2075 UV detector, Lablogic β-RAM Scintilation detector, XBridge C18, 4.6x100 mm, 100Å, 5µm, 45% MeCN, 55% water, 0.2% DIPEA, 1 ml/min, 254 nm) and found to be of 97.2% radiochemical purity. A small UV trace impurity was observed at 7.48 min (Figure S21), which was not the precursor. The molar activity was 2.8 MBq/nmol and the molar concentration was 7.93 nmol/mL, based on the applied [^{3}H]methyl nosylate.

^{1}H NMR spectrum of **5**

^{1}H NMR (600 MHz, DMSO) δ 14.49 (s, 1H), 9.63 (s, 1H), 7.44 (d, *J* = 8.9 Hz, 2H), 6.83 (d, *J* = 8.9 Hz, 2H), 2.46 (s, 3H), 2.38 (s, 3H).

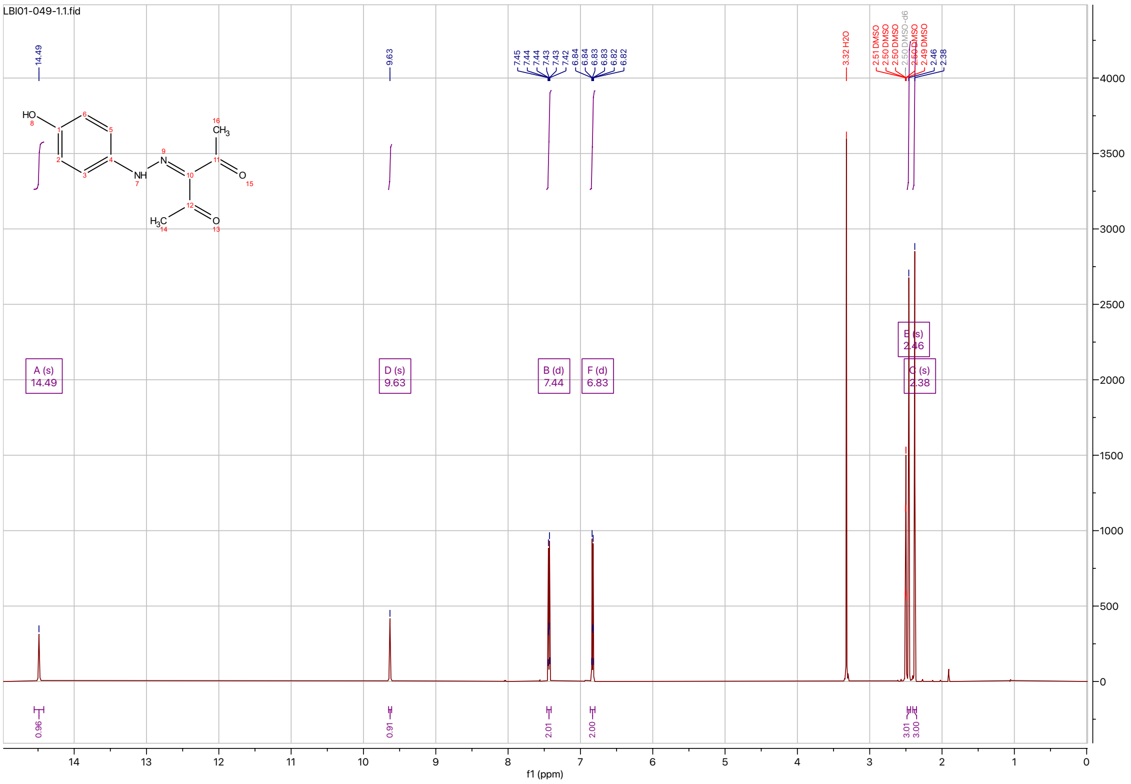

^{13}C NMR spectrum of **5**

^{13}C NMR (151 MHz, DMSO) δ 196.0, 195.9, 155.9, 133.7, 132.1, 118.1, 116.1, 31.0, 26.4.

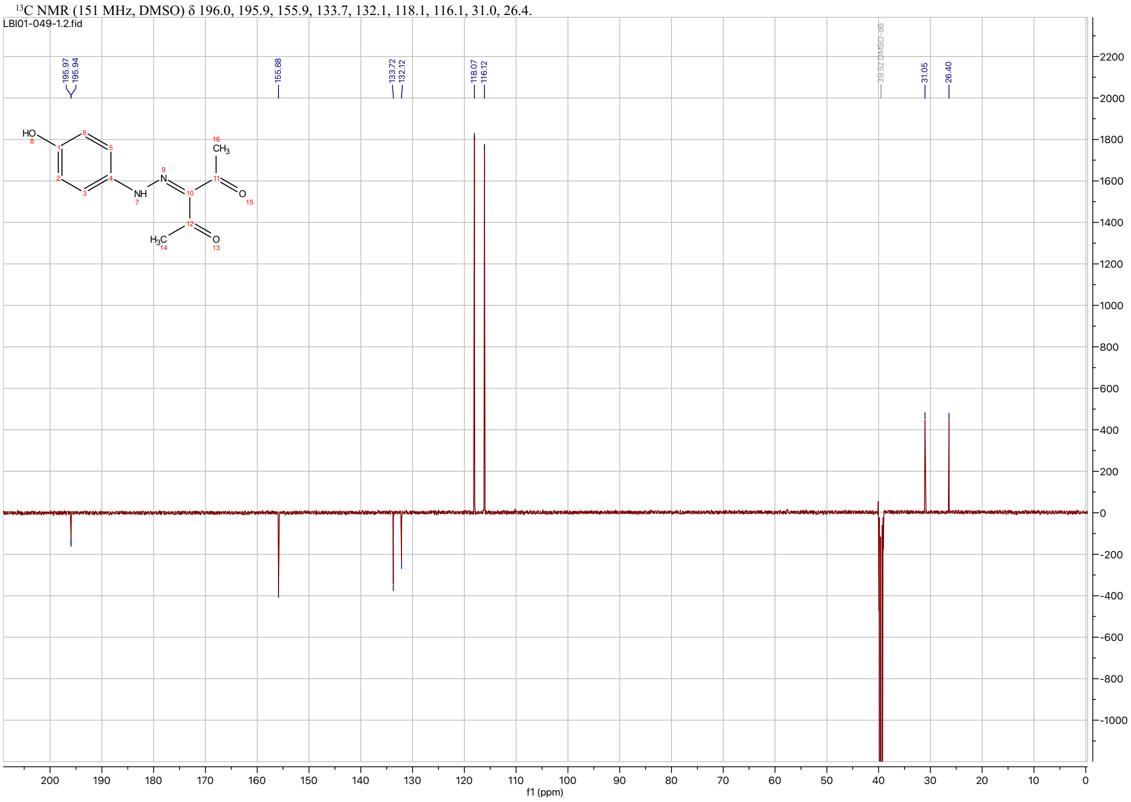

LCMS chromatogram of **5**

Acquired by : Admin
Date Acquired : 1/18/2022 4:51:41 PM
Sample Name : LBI01-049-1
Sample ID :
Tray# : 1
Vial# : 17
Injection Volume : 1
Data File : C:\LabSolutions\Data\2022\2022-wk03\LBI01-049-1.lcd
Background File : Blanco18012022.lcd
Method File : Method SCAN ACID standard azo.lcm
Report Format : DefaultLCMS.lcr
Tuning File : C:\LabSolutions\Tuning File\Tuning-ESI-pos-neg01072015.lct
Processed by : Admin
Modified Date : 1/18/2022 5:23:17 PM

Chromatogram
LBI01-049-1 C:\LabSolutions\Data\2022\2022 wk01-10\2022-wk03\LBI01-049-1.lcd

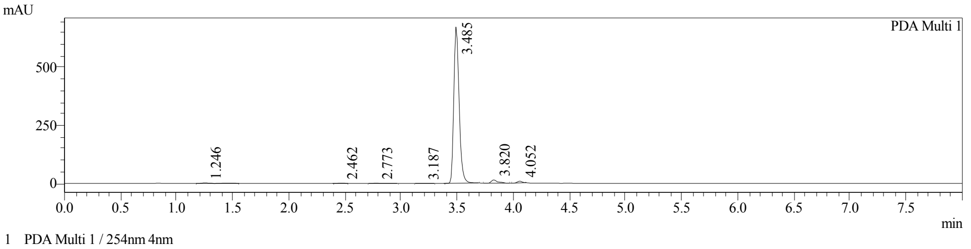

1 PDA Multi 1 / 254nm 4nm

PeakTable

PDA Ch1 254nm 4nm

Peak#	Ret. Time	Area	Height	Name	Area %
1	1.246	6205	2238		0.268
2	2.462	2196	761		0.095
3	2.773	1791	282		0.077
4	3.187	1219	213		0.053
5	3.485	2224933	675446		96.159
6	3.820	54602	13580		2.360
7	4.052	22869	7433		0.988
Total		2313814	699952		100.000

PeakTable

PDA Ch2 400nm 4nm

MS Chromatogram
LBI01-049-1 C:\LabSolutions\Data\2022\2022 wk01-10\2022-wk03\LBI01-049-1.lcd

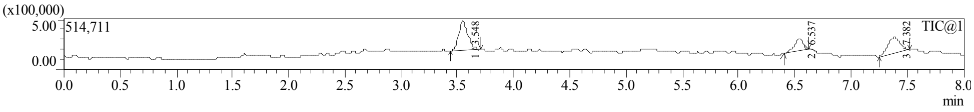

MS Spectrum Graph

#:1 Ret.Time:Averaged 3.540-3.560(Scan#:355-357)
BG Mode:Calc 3.440<->3.710(345<->372)
Mass Peaks:25 Base Peak:221.00(162547) Polarity:Pos Segment1 - Event1

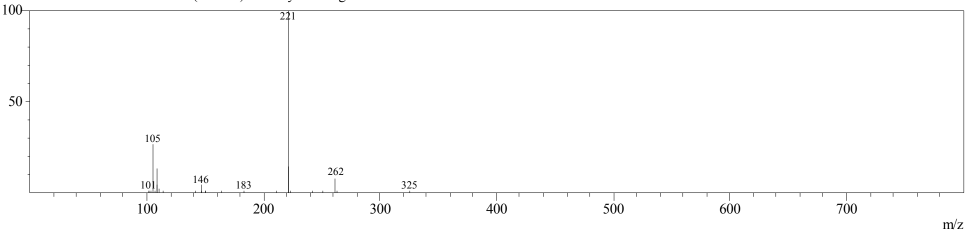

MS Spectrum Table

#:1 Ret.Time:
BG Mode:Calc 3.440<->3.710(345<->372)
Mass Peaks:25 Base Peak:221.00(162547) Polarity:Pos Segment1 - Event1

#	m/z	Abs.Inten.	Rel.Inten.	Charge	Polarity	Monoisotopic
1	101.05	2008	1.24			
2	102.05	2323	1.43			
3	103.25	2160	1.33			
4	104.95	43262	26.62			
5	106.05	1884	1.16			
6	107.95	6754	4.16			

^{1}H NMR spectrum of **6a**

^{1}H NMR (600 MHz, DMSO) δ 9.93 (s, 1H), 7.61 (d, J = 8.7 Hz, 2H), 6.87 (d, J = 8.9 Hz, 2H), 3.71 (s, 3H), 2.51 (s, 3H), 2.34 (s, 3H).

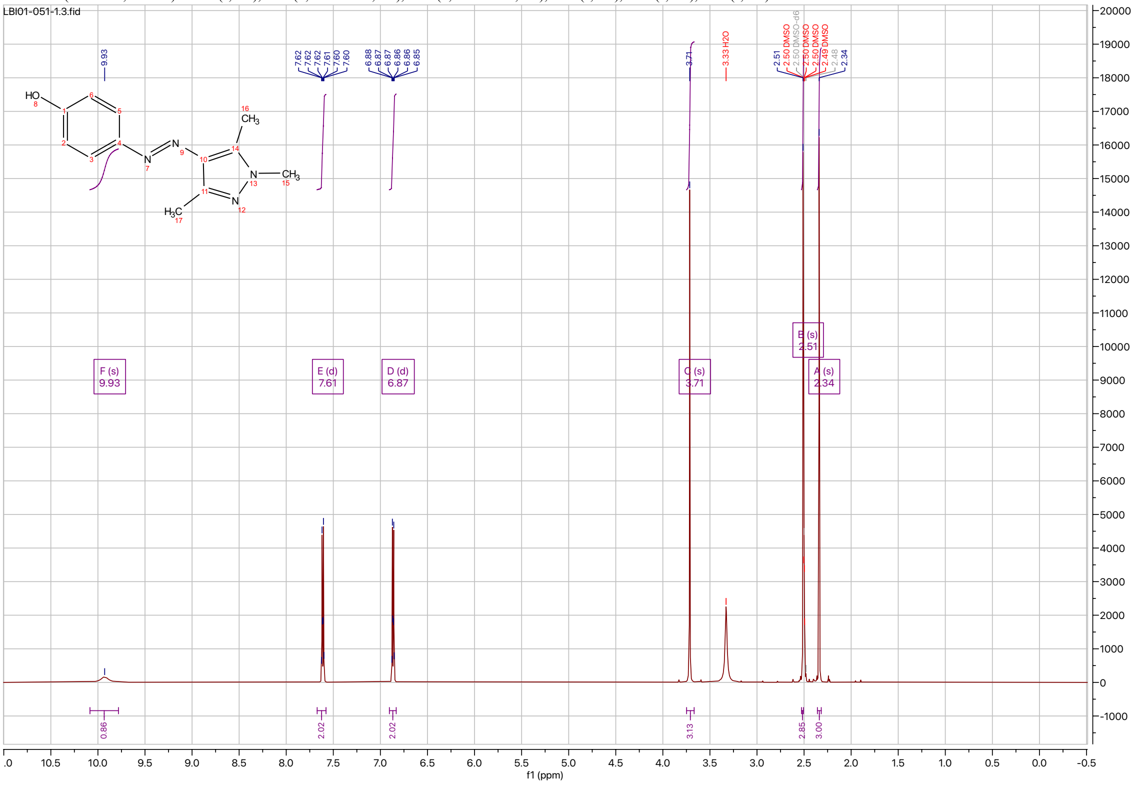

^{13}C NMR spectrum of **6a**

^{13}C NMR (151 MHz, DMSO) δ 159.1, 146.1, 139.9, 138.1, 134.0, 123.1, 115.6, 35.9, 13.7, 9.4.

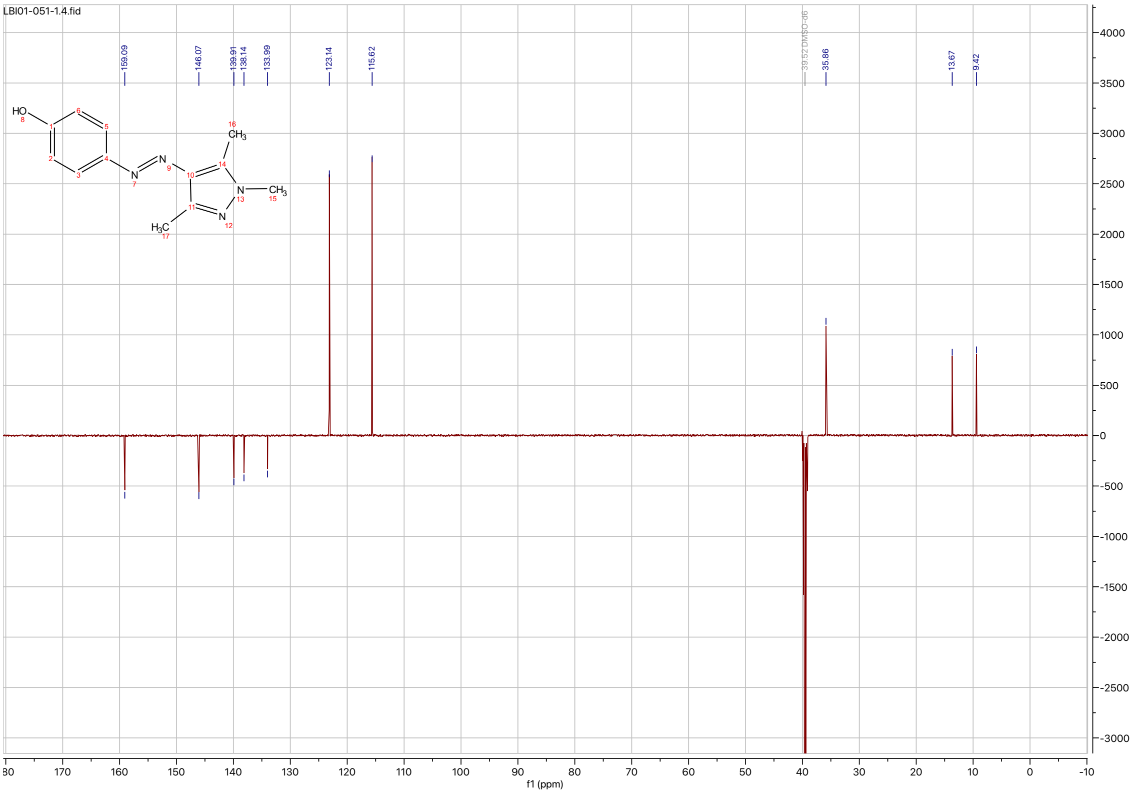

LCMS chromatogram of **6a**

Acquired by	: Admin
Date Acquired	: 7/24/2023 12:02:32 PM
Sample Name	: LBI01-051-1
Sample ID	:
Tray#	: 1
Vial#	: 5
Injection Volume	: 1
Data File	: C:\LabSolutions\Data\2023\2023-wk30\LBI01-051-1.lcd
Background File	: blanco 24072023.lcd
Method File	: Method SCAN ACID standard azo.lcm
Report Format	: DefaultLCMS.lcr
Tuning File	: C:\LabSolutions\Tuning File\Tuning-ESI-pos-neg01072015.lct
Processed by	: Admin
Modified Date	: 7/24/2023 12:11:57 PM

Chromatogram
LBI01-051-1 C:\LabSolutions\Data\2023\2023-wk30\LBI01-051-1.lcd

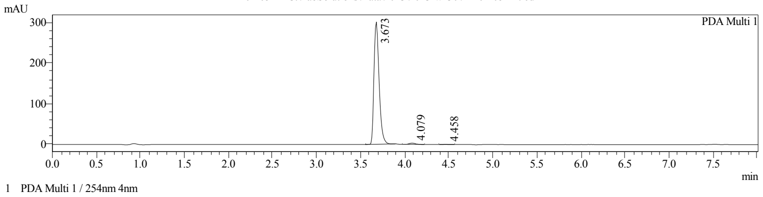

1 PDA Multi 1 / 254nm 4nm

PeakTable

PDA Ch1 254nm 4nm

Peak#	Ret. Time	Area	Height	Name	Area %
1	3.673	1210573	300102		98.785
2	4.079	13797	3052		1.126
3	4.458	1088	331		0.089
Total		1225458	303485		100.000

PeakTable

PDA Ch2

MS Chromatogram
LBI01-051-1 C:\LabSolutions\Data\2023\2023-wk30\LBI01-051-1.lcd

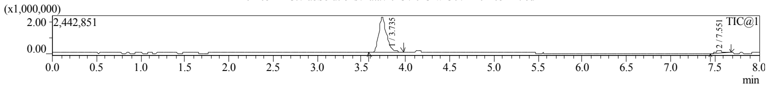

MS Spectrum Graph

#:1 Ret.Time:Averaged 3.720-3.740(Scan#:373-375)
BG Mode:Calc 3.590<->3.970(360<->398)
Mass Peaks:6 Base Peak:231.10(1743706) Polarity:Pos Segment1 - Event1

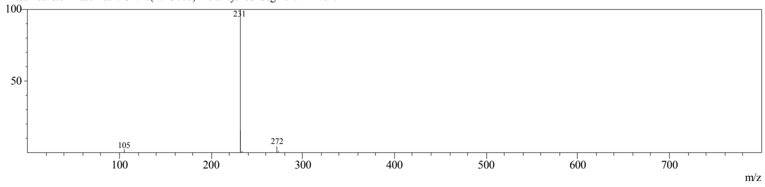

MS Spectrum Table

#:1 Ret.Time:
BG Mode:Calc 3.590<->3.970(360<->398)
Mass Peaks:6 Base Peak:231.10(1743706) Polarity:Pos Segment1 - Event1

#	m/z	Abs.Inten.	Rel.Inten.	Charge	Polarity	Monoisotopic	#	m/z	Abs.Inten.	Rel.Inten.	Charge	Polarity	Monoisotopic
1	105.00	37306	2.14				4	233.15	18381	1.05			
2	231.10	1743706	100.00				5	272.10	84894	4.87			
3	232.10	277244	15.90				6	273.15	21363	1.23			

^{1}H NMR spectrum of **6b**

^{1}H NMR (500 MHz, $CDCl_3$) δ 7.71 (d, J = 9.0 Hz, 2H), 6.90 (d, J = 8.7 Hz, 2H), 6.76 (s, 1H), 4.00 (dddd, J = 11.7, 11.7, 3.8, 3.8 Hz, 1H), 2.58 (s, 3H), 2.50 (s, 3H), 2.05 – 1.95 (m, 2H), 1.95 – 1.85 (m, 4H), 1.74 – 1.71 (m, 1H), 1.44 – 1.35 (m, 2H), 1.29 (dddd, J = 11.9, 11.9, 2.5, 2.5 Hz, 1H).

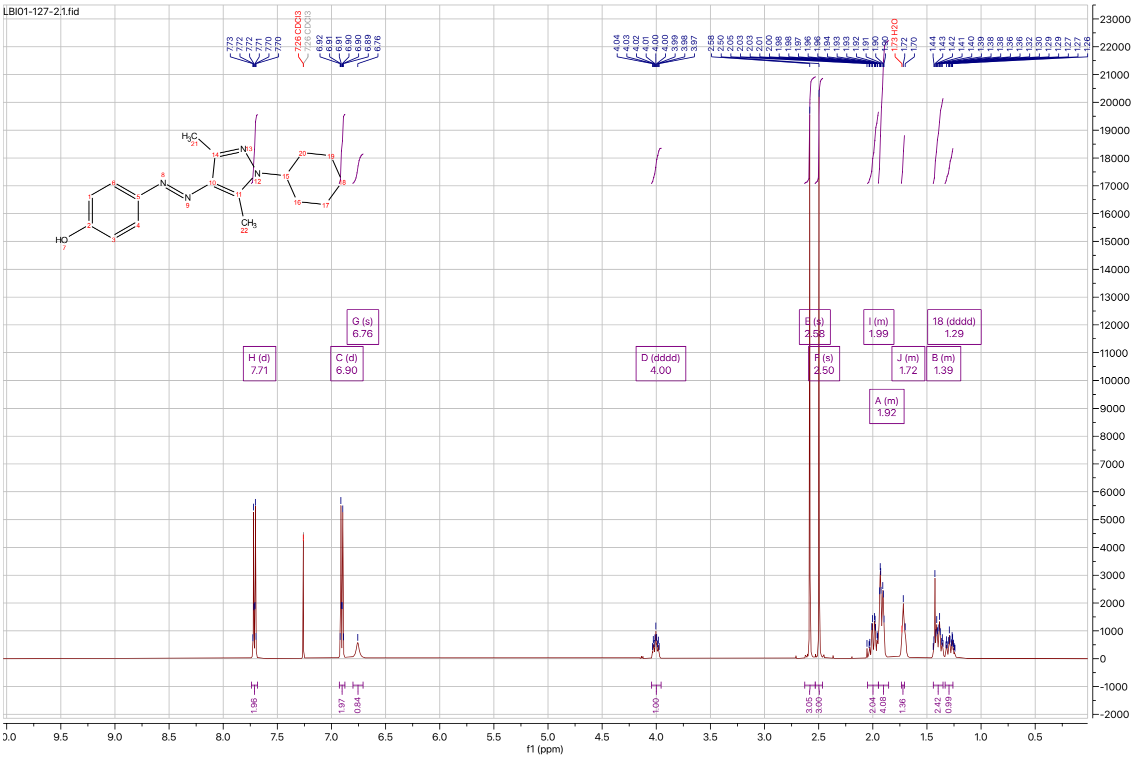

^{13}C NMR spectrum of **6b**

^{13}C NMR (126 MHz, $CDCl_3$) δ 157.6, 148.0, 142.0, 137.3, 134.7, 123.6, 115.8, 57.9, 32.6, 25.9, 25.3, 14.0, 9.8.

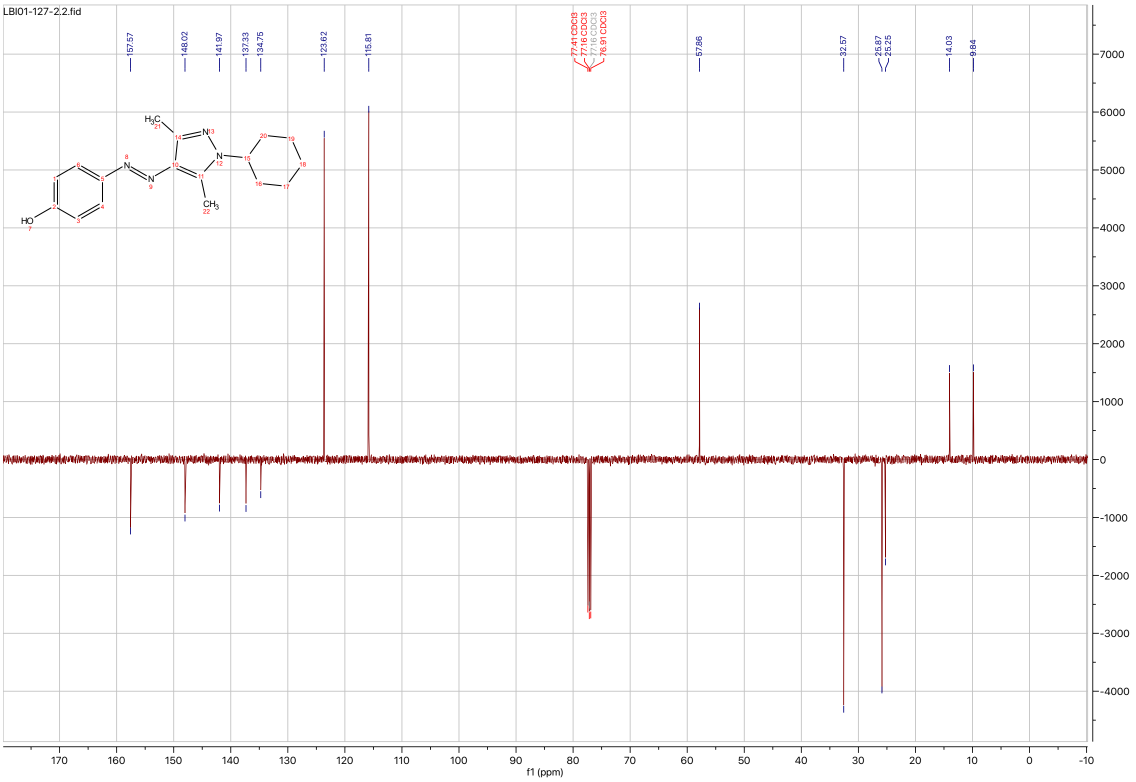

LCMS chromatogram of **6b**

Acquired by : Admin
Date Acquired : 7/21/2023 3:36:56 PM
Sample Name : LBI01-127-2
Sample ID :
Tray# : 1
Vial# : 12
Injection Volume : 1
Data File : C:\LabSolutions\Data\2023\2023-wk29\LBI01-127-2.lcd
Background File : azoblanco 21072023.lcd
Method File : Method SCAN ACID standard azo.lcm
Report Format : DefaultLCMS.lcr
Tuning File : C:\LabSolutions\Tuning File\Tuning-ESI-pos-neg01072015.lct
Processed by : Admin
Modified Date : 7/21/2023 3:52:19 PM

Chromatogram
LBI01-127-2 C:\LabSolutions\Data\2023\2023-wk29\LBI01-127-2.lcd

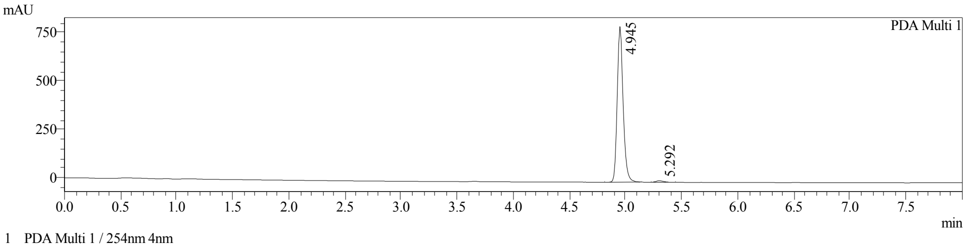

1 PDA Multi 1 / 254nm 4nm

PeakTable

PDA Ch1 254nm 4nm

Peak#	Ret. Time	Area	Height	Name	Area %
1	4.945	2983691	802198		98.785
2	5.292	36689	7511		1.215
Total		3020380	809709		100.000

PeakTable

PDA Ch2

MS Chromatogram
LBI01-127-2 C:\LabSolutions\Data\2023\2023-wk29\LBI01-127-2.lcd

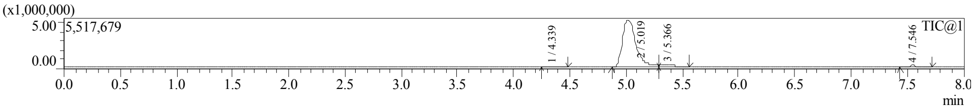

MS Spectrum Graph

#:1 Ret.Time:Averaged 5.010-5.030(Scan#:502-504)
BG Mode:Calc 4.870<->5.290(488<->530)
Mass Peaks:7 Base Peak:299.15(3688844) Polarity:Pos Segment1 - Event1

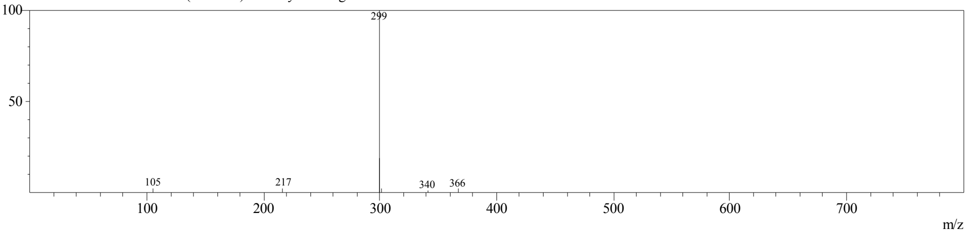

^{1}H NMR spectrum of **6c**

^{1}H NMR (600 MHz, $CDCl_3$) δ 7.72 (d, 2H), 6.90 (d, 2H), 5.69 (s, 1H), 4.24 (dddd, J = 11.6, 11.6, 4.1, 4.1 Hz, 1H), 4.15 (dd△, J = 11.4, 4.5 Hz, 2H), 3.55 (ddd, J = 12.2, 12.1, 1.9 Hz, 2H), 2.61 (s, 3H), 2.50 (s, 3H), 2.37 (dddd, J = 12.3, 12.0, 12.0, 4.6 Hz, 2H), 1.84 (ddd△, J = 13.0, 4.3, 2.0 Hz, 2H).

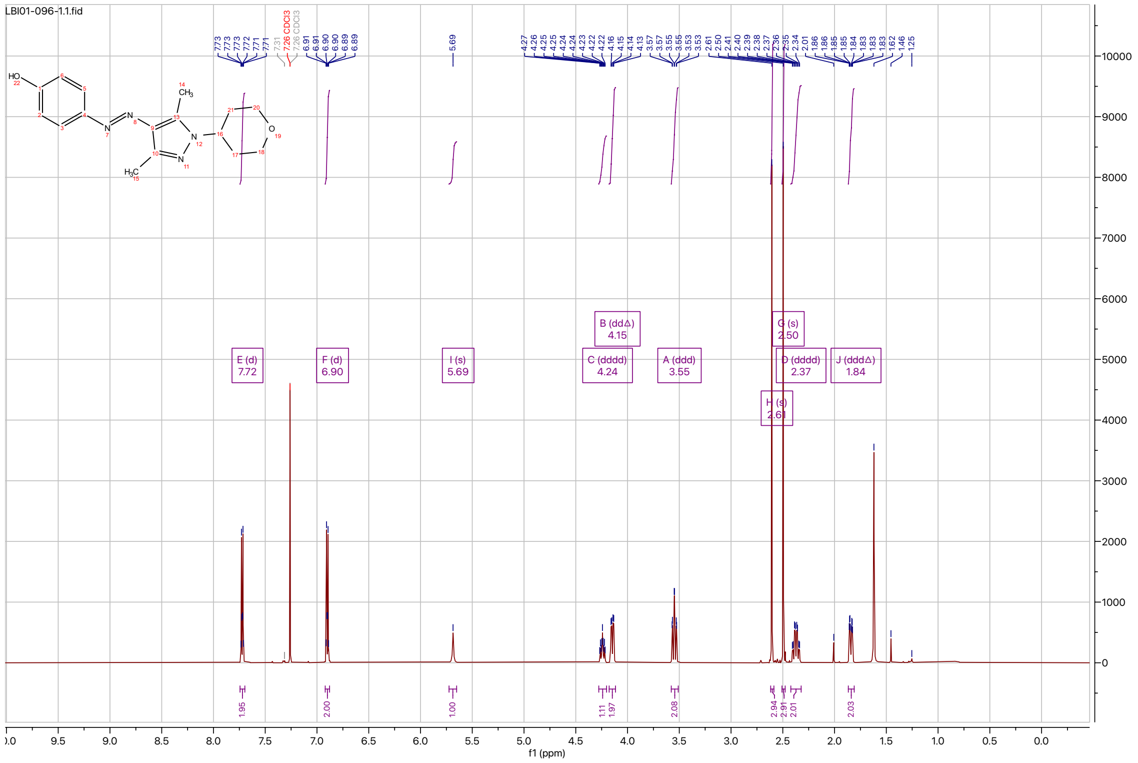

^{13}C NMR spectrum of **6c**

^{13}C NMR (151 MHz, $CDCl_3$) δ 157.2, 148.2, 142.2, 137.4, 135.0, 123.7, 115.7, 67.3, 54.9, 32.5, 14.2, 9.8.

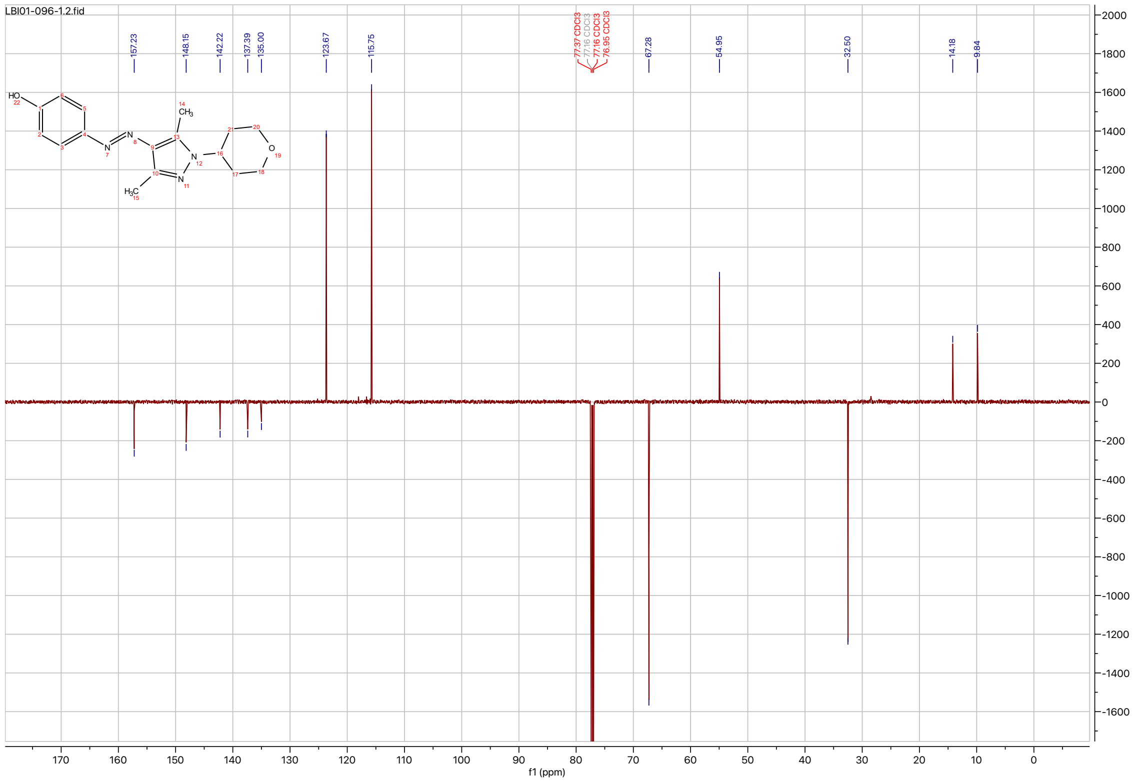

LCMS chromatogram of **6c**

Acquired by : Admin
Date Acquired : 9/15/2022 2:30:22 PM
Sample Name : LBI01-096-2
Sample ID :
Tray# : 1
Vial# : 31
Injection Volume : 1
Data File : C:\LabSolutions\Data\2022\2022-wk37\LBI01-096-2.lcd
Background File : azoblanco 15092022.lcd
Method File : Method SCAN ACID standard azo.lcm
Report Format : DefaultLCMS.lcr
Tuning File : C:\LabSolutions\Tuning File\Tuning-ESI-pos-neg01072015.lct
Processed by : Admin
Modified Date : 9/15/2022 2:39:32 PM

Chromatogram
LBI01-096-2 C:\LabSolutions\Data\2022\2022-wk37\LBI01-096-2.lcd

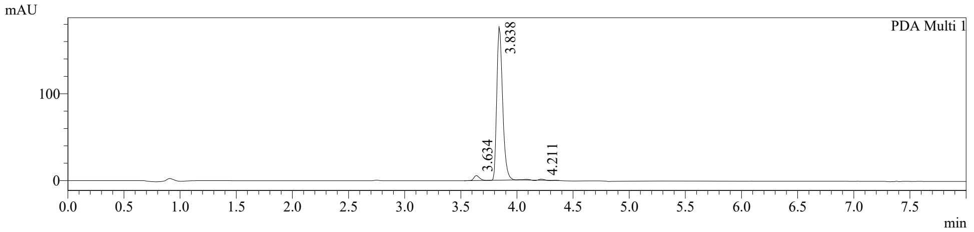

1 PDA Multi 1 / 254nm 4nm

PeakTable

PDA Ch1 254nm 4nm

Peak#	Ret. Time	Area	Height	Area %
1	3.634	20805	5863	3.134
2	3.838	637223	177089	95.979
3	4.211	5891	1239	0.887
Total		663919	184191	100.000

MS Chromatogram
LBI01-096-2 C:\LabSolutions\Data\2022\2022-wk37\LBI01-096-2.lcd

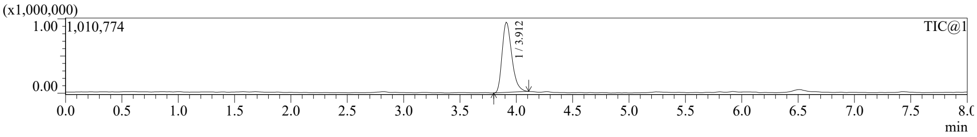

MS Spectrum Graph

#:1 Ret.Time:Averaged 3.900-3.920(Scan#:391-393)
BG Mode:Calc 3.800<->4.110(381<->412)
Mass Peaks:4 Base Peak:301.15(790442) Polarity:Pos Segment1 - Event1

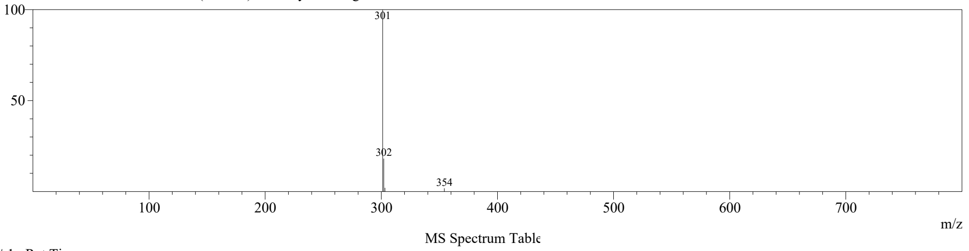

MS Spectrum Table

#:1 Ret.Time:
BG Mode:Calc 3.800<->4.110(381<->412)
Mass Peaks:4 Base Peak:301.15(790442) Polarity:Pos Segment1 - Event

#	m/z	Abs.Inten.	Rel.Inten.	Charge	Polarity	Monoisotopic	#	m/z	Abs.Inten.	Rel.Inten.	Charge	Polarity	Monoisotopic
1	301.15	790442	100.00				3	303.15	16463	2.08			
2	302.15	142942	18.08				4	354.25	14162	1.79			

^{1}H NMR spectrum of **6d**

^{1}H NMR (500 MHz, $CDCl_3$) δ 7.72 (d, J = 8.8 Hz, 2H), 6.90 (d, J = 8.8 Hz, 2H), 4.44 – 4.20 (m, 2H), 4.15 (dddd, J = 11.6, 11.6, 4.1, 4.1 Hz, 1H), 2.96 – 2.77 (m, 2H), 2.59 (s, 3H), 2.48 (s, 3H), 2.25 – 2.11 (m, 2H), 1.91 – 1.83 (m, 2H), 1.48 (s, 9H).

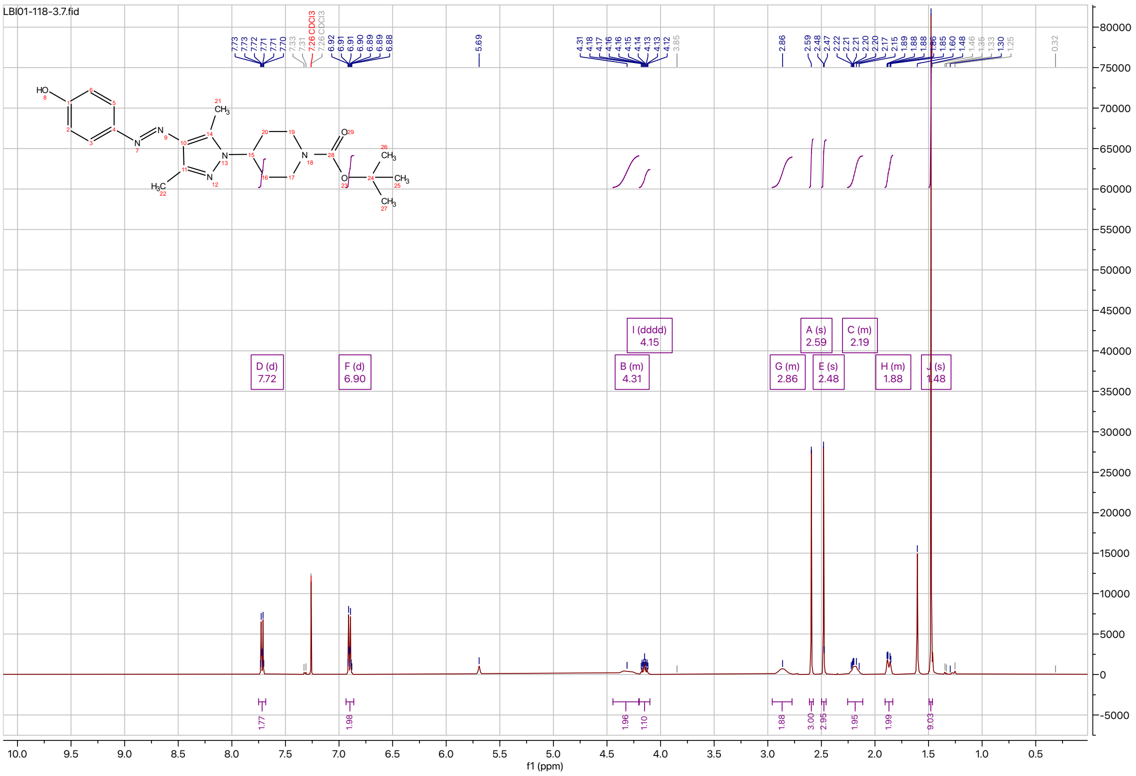

^{13}C NMR spectrum of **6d**

^{13}C NMR (126 MHz, $CDCl_3$) δ 157.3, 154.6, 148.2, 142.2, 137.4, 135.0, 123.7, 115.7, 80.1, 77.4, 77.2, 76.9, 55.9, 42.9#, 31.6, 28.6, 14.2, 9.8.

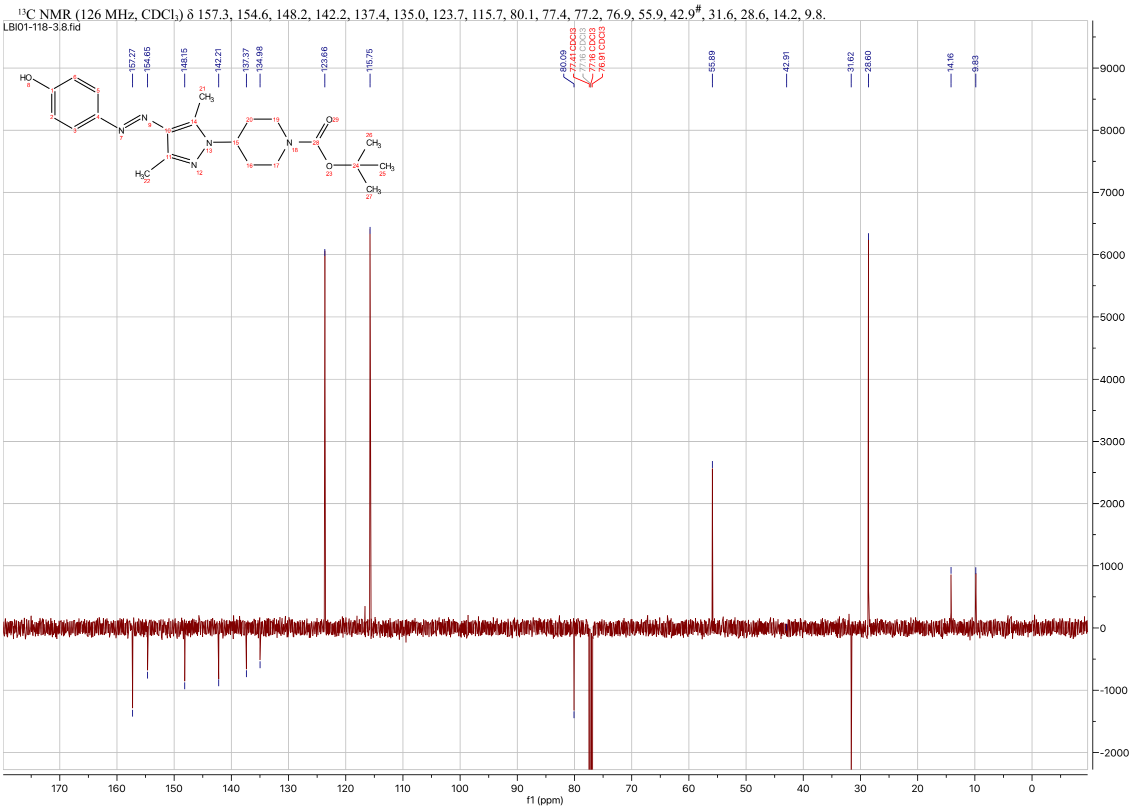

LCMS chromatogram of **6d**

Acquired by	: Admin
Date Acquired	: 12/16/2022 2:08:17 PM
Sample Name	: LBI01-118-2
Sample ID	:
Tray#	: 1
Vial#	: 33
Injection Volume	: 1
Data File	: C:\LabSolutions\Data\2022\2022-wk41-50\2022-wk50\LBI01-118-2.lcd
Background File	: blanco 16122022.lcd
Method File	: Method SCAN ACID standard azo.lcm
Report Format	: DefaultLCMS.lcr
Tuning File	: C:\LabSolutions\Tuning File\Tuning-ESI-pos-neg01072015.lct
Processed by	: Admin
Modified Date	: 12/16/2022 2:31:21 PM

Chromatogram
LBI01-118-2 C:\LabSolutions\Data\2022\2022-wk41-50\2022-wk50\LBI01-118-2.lcd

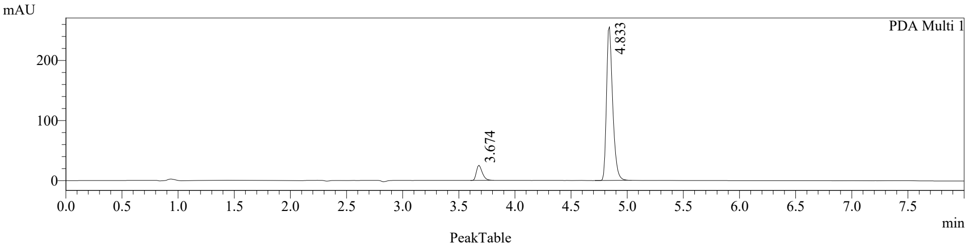

PeakTable

PDA Ch1 254nm 4nm

Peak#	Ret. Time	Area	Height	Area %
1	3.674	91056	25241	8.520
2	4.833	977649	256025	91.480
Total		1068705	281266	100.000

MS Chromatogram
LBI01-118-2 C:\LabSolutions\Data\2022\2022-wk41-50\2022-wk50\LBI01-118-2.lcd

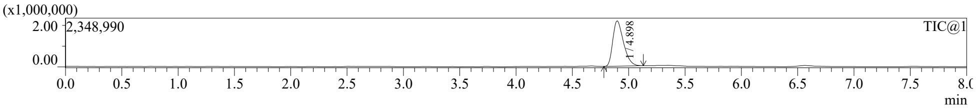

MS Spectrum Graph

#:1 Ret.Time:Averaged 4.890-4.910(Scan#:490-492)
BG Mode:Calc 4.780<->5.130(479<->514)
Mass Peaks:10 Base Peak:400.25(1101337) Polarity:Pos Segment1 - Event1

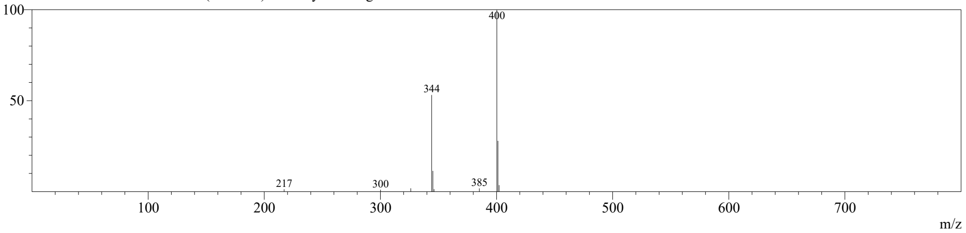

MS Spectrum Table

#:1 Ret.Time:
BG Mode:Calc 4.780<->5.130(479<->514
Mass Peaks:10 Base Peak:400.25(1101337) Polarity:Pos Segment1 - Event

#	m/z	Abs.Inten.	Rel.Inten.	Charge	Polarity	Monoisotopic	#	m/z	Abs.Inten.	Rel.Inten.	Charge	Polarity	Monoisotopic
1	217.10	14992	1.36				6	346.20	15298	1.39			
2	300.15	11689	1.06				7	385.20	20086	1.82			
3	326.15	20250	1.84				8	400.25	1101337	100.00			
4	344.15	585539	53.17				9	401.25	308004	27.97			
5	345.15	126245	11.46				10	402.25	39905	3.62			

^{1}H NMR spectrum of **6f**

^{1}H NMR (500 MHz, DMSO) δ 9.91 (s, 1H), 7.61 (d, *J* = 8.8 Hz, 2H), 6.86 (d, *J* = 8.9 Hz, 2H), 4.12 (dddd, *J* = 10.9, 10.9, 5.1, 5.1 Hz, 1H), 2.92 – 2.82 (m, 2H), 2.55 (s, 3H), 2.35 (s, 3H), 2.21 (s, 3H), 2.12 – 2.00 (m, 4H), 1.84 – 1.75 (m, 2H).

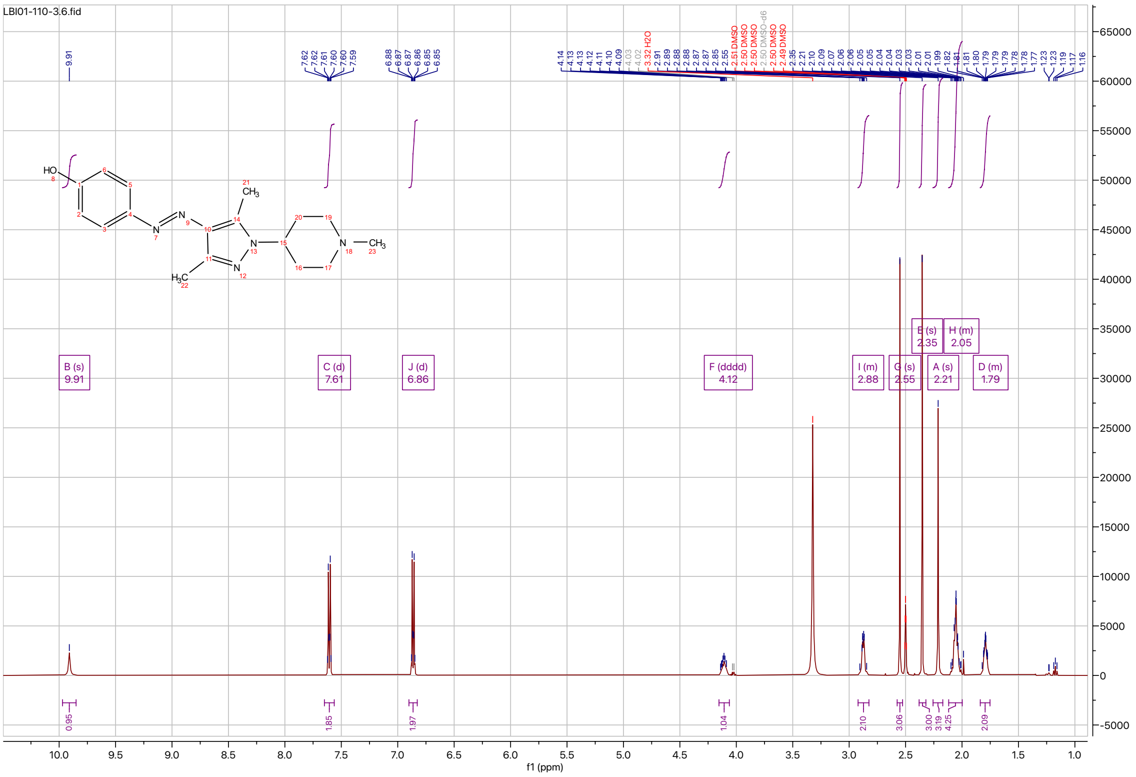

^{13}C NMR spectrum of **6f**

^{13}C NMR (126 MHz, DMSO) δ 159.0, 146.1, 139.7, 137.8, 133.9, 123.1, 115.6, 54.5, 54.3, 45.9, 31.1, 14.2, 9.0.

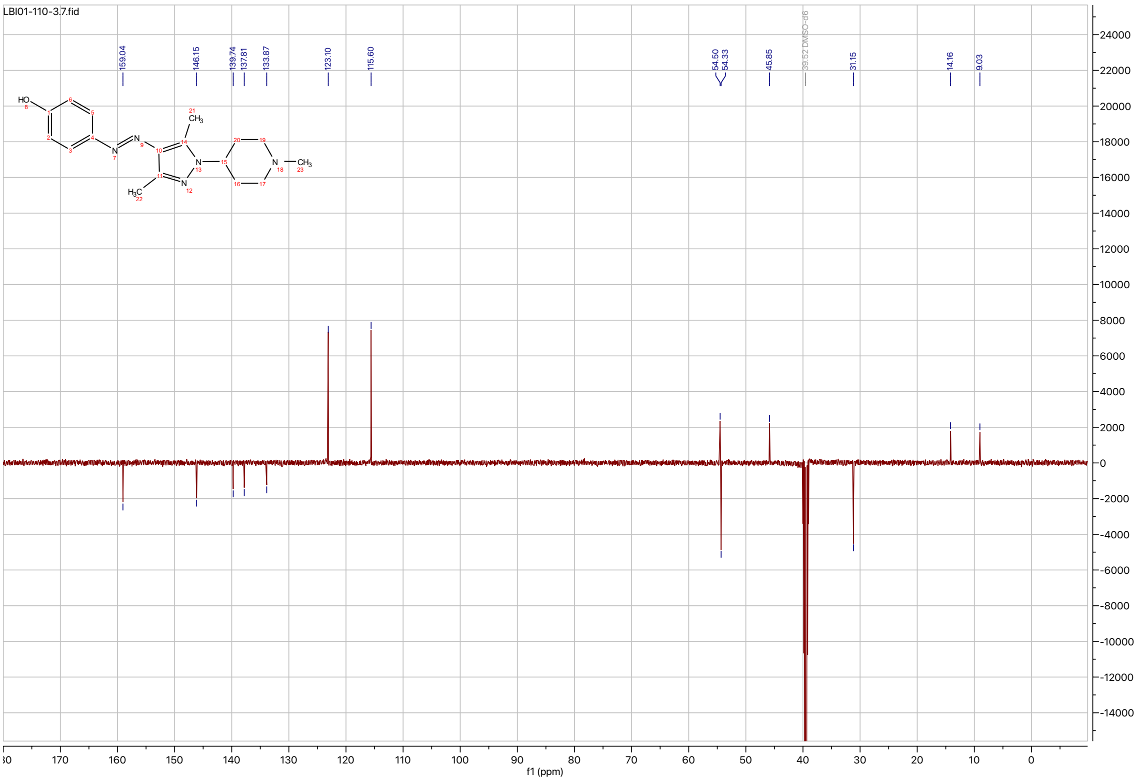

LCMS chromatogram of **6f**

Acquired by	: Admin
Date Acquired	: 12/19/2022 5:26:40 PM
Sample Name	: LBI01-110-3
Sample ID	:
Tray#	: 1
Vial#	: 20
Injection Volume	: 1
Data File	: C:\LabSolutions\Data\2022\2022-wk51\LBI01-110-3.lcd
Background File	: blanco 19122022.lcd
Method File	: Method SCAN ACID standard azo.lcm
Report Format	: DefaultLCMS.lcr
Tuning File	: C:\LabSolutions\Tuning File\Tuning-ESI-pos-neg01072015.lct
Processed by	: Admin
Modified Date	: 12/19/2022 5:53:35 PM

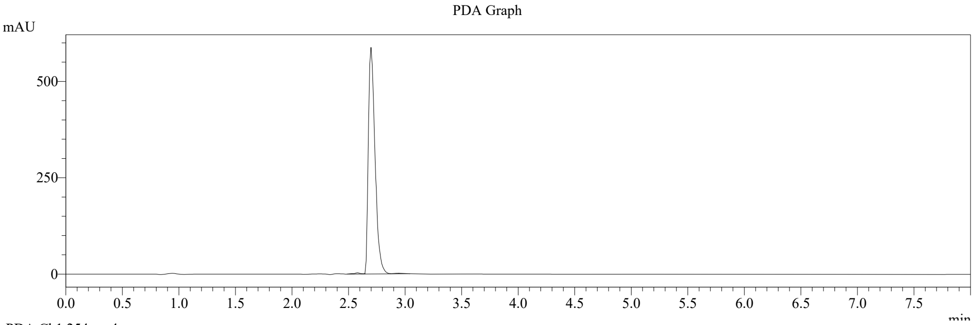

PDA Ch1 254nm 4nm

Peak#	Name	Ret. Time	Area	Area %
1		2.574	14158	0.578
2		2.693	2424929	99.020
3		2.937	9841	0.402

MS Spectrum Graph
#:1 Ret.Time:Averaged 2.760-2.780(Scan#:277-279
Mass Peaks:3 Base Peak:314.20(4038889) Polarity:Pos Segment1 - Event1

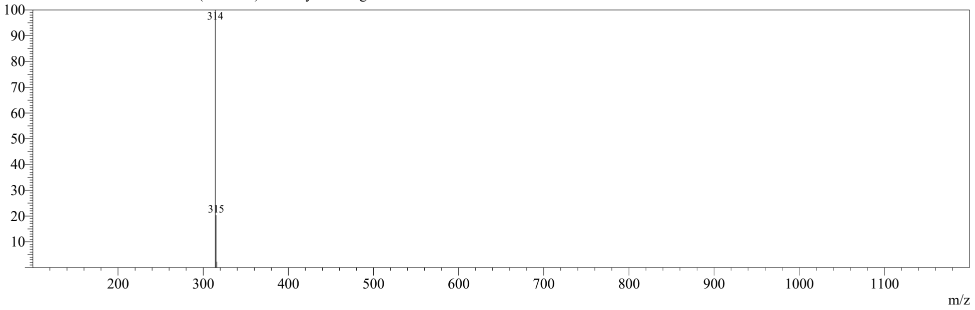

MS Spectrum Table
#:1 Ret.Time:
BG Mode:Calc 2.630<->3.180(264<->319
Mass Peaks:3 Base Peak:314.20(4038889) Polarity:Pos Segment1 - Event

#	m/z	Abs.Inten.	Rel.Inten.	Charge	Polarity	Monoisotopic	#	m/z	Abs.Inten.	Rel.Inten.	Charge	Polarity	Monoisotopic
1	314.20	4038889	100.00				3	316.20	91962	2.28			
2	315.20	821404	20.34										

^{1}H NMR spectrum of **3a**

^{1}H NMR (600 MHz, MeOD) δ 7.79 – 7.70 (m, 2H), 6.96 – 6.88 (m, 2H), 6.71 (s, 2H), 4.93 (ddd, J = 7.0, 4.7, 2.1 Hz, 1H), 3.92 – 3.84 (m, 1H), 3.77 (s, 3H), 2.85 – 2.73 (m, 2H), 2.62 – 2.52 (m, 5H), 2.43 (s, 3H), 1.99 – 1.55 (m, 6H).

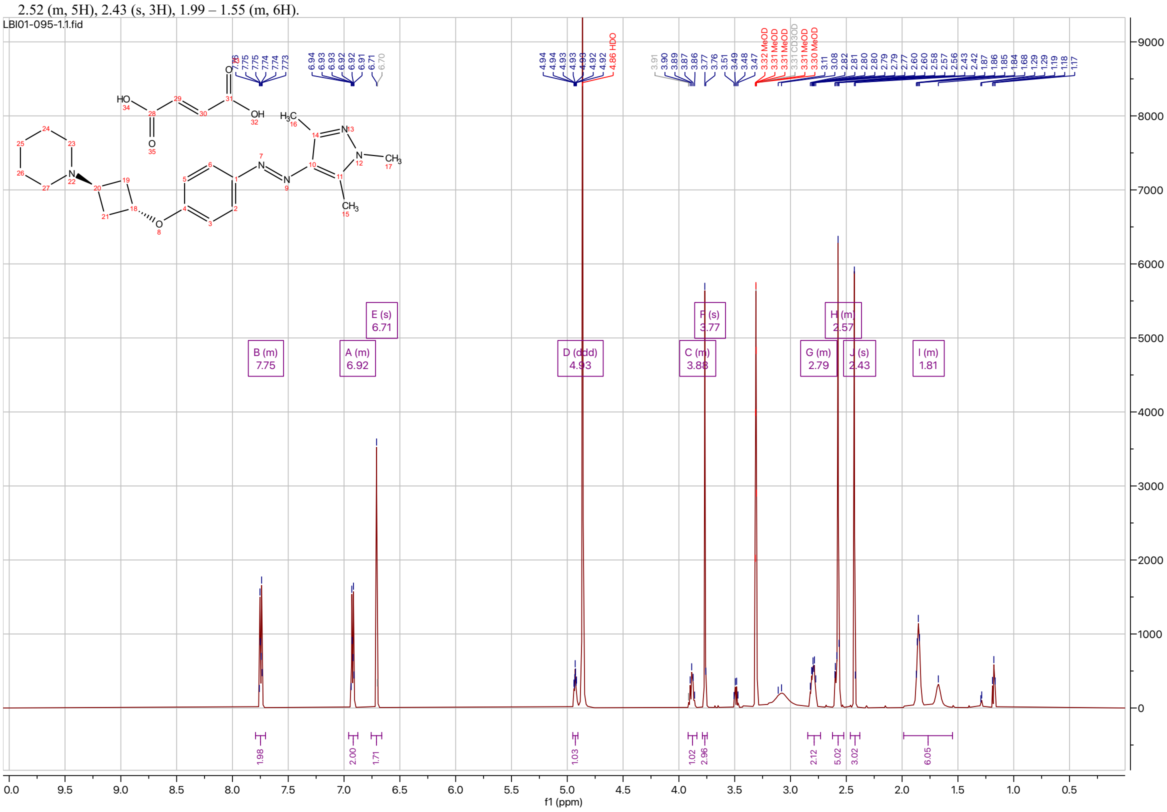

^{13}C NMR spectrum of **3a**

^{13}C NMR (151 MHz, MeOD) δ 171.4, 159.7, 149.5, 142.8, 140.4, 136.3, 135.7, 124.4, 116.3, 68.9, 58.6, 51.7, 49.4, 49.3, 49.1, 49.0, 48.9, 48.7, 36.0, 33.3, 24.2, 22.9, 13.8, 9.7.

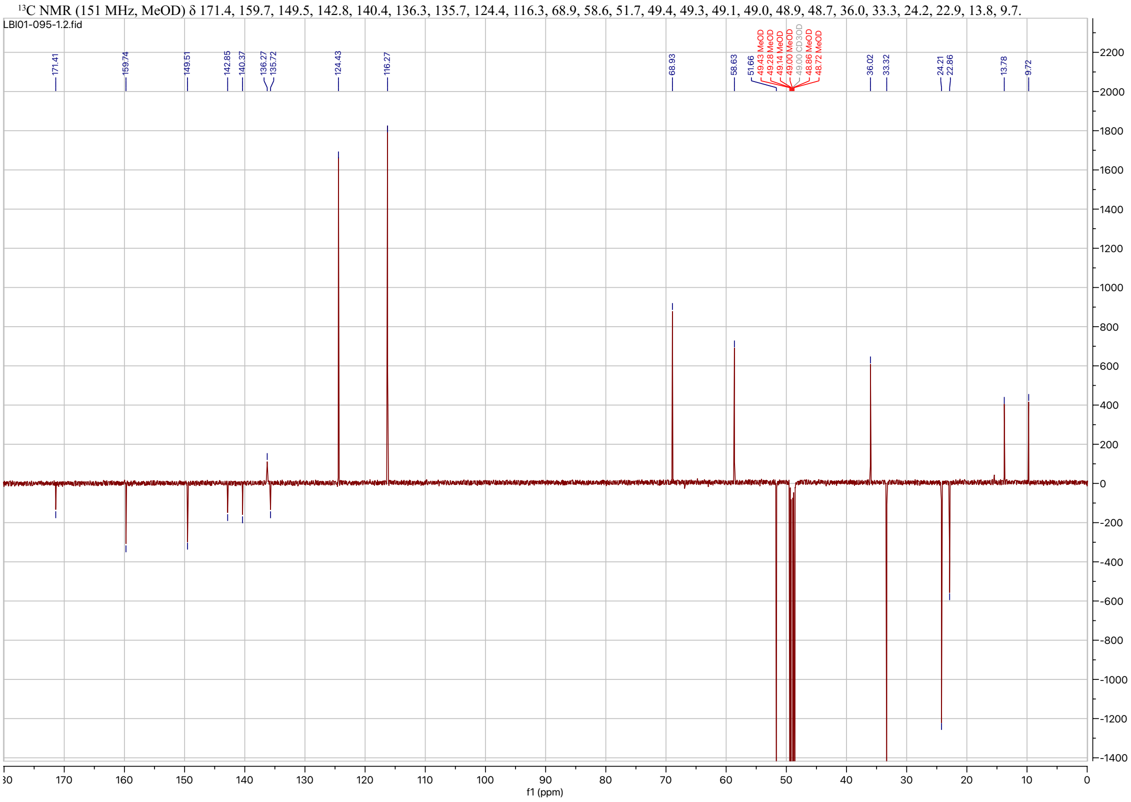

LCMS chromatogram of **3a**

Acquired by	: Admin
Date Acquired	: 7/28/2022 4:06:01 PM
Sample Name	: LBI01-095-1
Sample ID	:
Tray#	: 1
Vial#	: 31
Injection Volume	: 1
Data File	: C:\LabSolutions\Data\2022\2022-wk30\LBI01-095-1.lcd
Background File	: azoblanco 28072022.lcd
Method File	: Method SCAN ACID standard azo.lcm
Report Format	: DefaultLCMS.lcr
Tuning File	: C:\LabSolutions\Tuning File\Tuning-ESI-pos-neg01072015.lct
Processed by	: Admin
Modified Date	: 7/28/2022 4:15:48 PM

Medicinal Chemistry
Vrije Universiteit Amsterdam

Chromatogram
LBI01-095-1 C:\LabSolutions\Data\2022\2022-wk30\LBI01-095-1.lcd

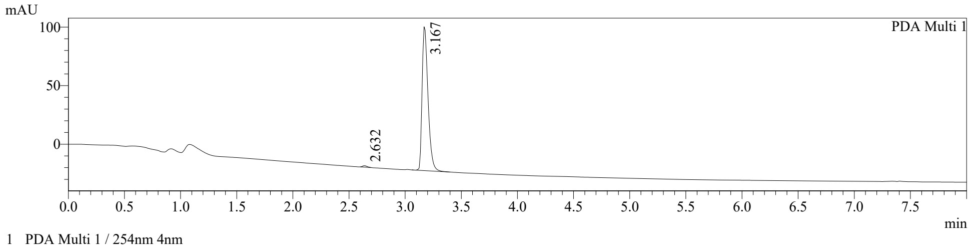

1 PDA Multi 1 / 254nm 4nm

PeakTable

PDA Ch1 254nm 4nm

Peak#	Ret. Time	Area	Height	Area %	Height %
1	2.632	3322	1283	0.729	1.035
2	3.167	452390	122749	99.271	98.965
Total		455712	124032	100.000	100.000

MS Chromatogram
LBI01-095-1 C:\LabSolutions\Data\2022\2022-wk30\LBI01-095-1.lcd

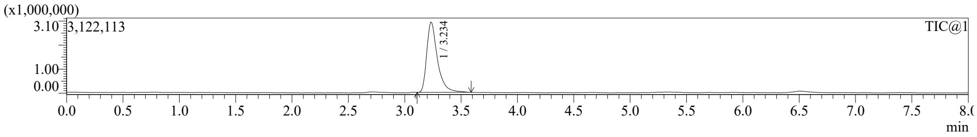

MS Spectrum Graph

#:1 Ret.Time:Averaged 3.220-3.240(Scan#:323-325)
BG Mode:Calc 3.110<->3.590(312<->360)
Mass Peaks:7 Base Peak:368.25(2137849) Polarity:Pos Segment1 - Event1

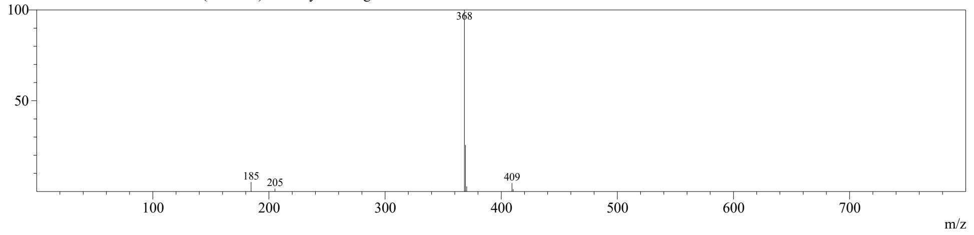

MS Spectrum Table

#:1 Ret.Time:
BG Mode:Calc 3.110<->3.590(312<->360
Mass Peaks:7 Base Peak:368.25(2137849) Polarity:Pos Segment1 - Event

#	m/z	Abs.Inten.	Rel.Inten.	Charge	Polarity	Monoisotopic
1	184.65	113243	5.30			
2	205.20	35789	1.67			
3	368.25	2137849	100.00			
4	369.25	547840	25.63			

#	m/z	Abs.Inten.	Rel.Inten.	Charge	Polarity	Monoisotopic
5	370.25	60822	2.85			
6	409.25	100227	4.69			
7	410.30	29289	1.37			

HRMS spectrum of **3a**

HRMS MedChem

**Analysis Info**		Acquisition Date	4/11/2023 9:41:02 AM
Analysis Name	D:\Data\ServiceMS\Hans\2023-wk15\VUF26010_4-11-2023_09-40-16_100-1200mzrange.d		
Method	100-1200mz range.m	Operator	Demo User
Sample Name	VUF26010	Instrument	impact II
Comment			

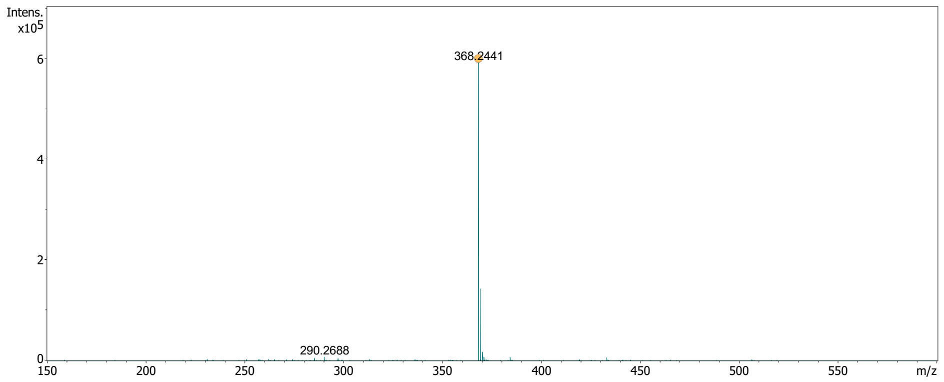

Meas. m/z	#	Ion Formula	m/z	err [ppm]	mSigma	#mSigma	Score	rdb	e⁻ Conf	N-Rule
368.2441	1	C21H30N5O	368.2445	0.9	4.4	1	100.00	10.0	even	ok

^{1}H NMR spectrum of **3b**

^{1}H NMR (500 MHz, MeOD) δ 7.74 (d, J = 9.5 Hz, 2H), 6.93 (d, J = 9.5 Hz, 2H), 6.71 (s, 2H), 4.93 (tt, J = 6.9, 2.2 Hz, 1H), 4.21 – 4.10 (m, 1H), 3.92 (tt, J = 8.0, 8.0 Hz, 1H), 3.18 – 3.02 (m, 2H), 2.80 (ddd, J = 11.2, 9.1, 5.2 Hz, 2H), 2.63 – 2.55 (m, 5H), 2.44 (s, 3H), 1.97 – 1.79 (m, 10H), 1.79 – 1.60 (m, 3H), 1.55 – 1.45 (m, 2H), 1.32 (dddd, J = 16.6, 13.1, 8.1, 4.7 Hz, 2H).

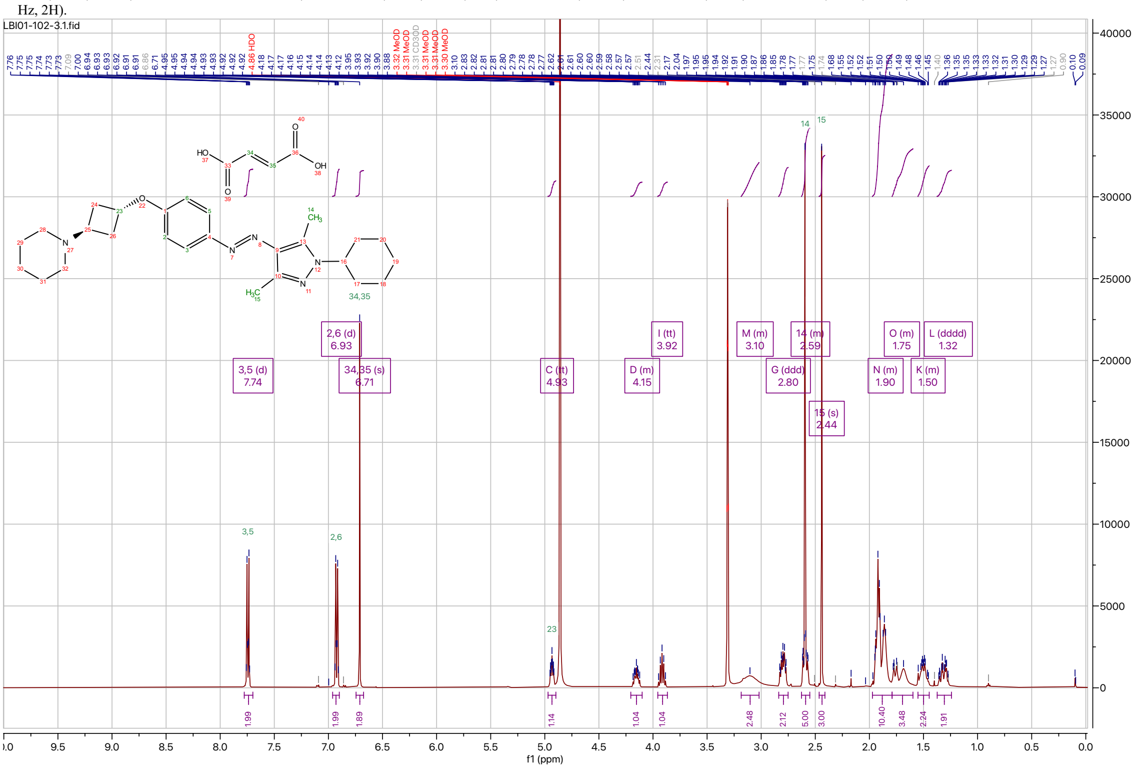

^{13}C NMR spectrum of **3b**

^{13}C NMR (126 MHz, MeOD) δ 169.6, 158.2, 148.2, 141.2, 138.2, 134.7, 134.1, 123.0, 114.9, 67.5, 57.3, 57.3, 50.3, 48.1, 47.9, 47.8, 47.6, 47.4, 47.3, 47.1, 32.1, 31.9, 25.2, 25.0, 22.8, 21.4, 12.7, 8.1.

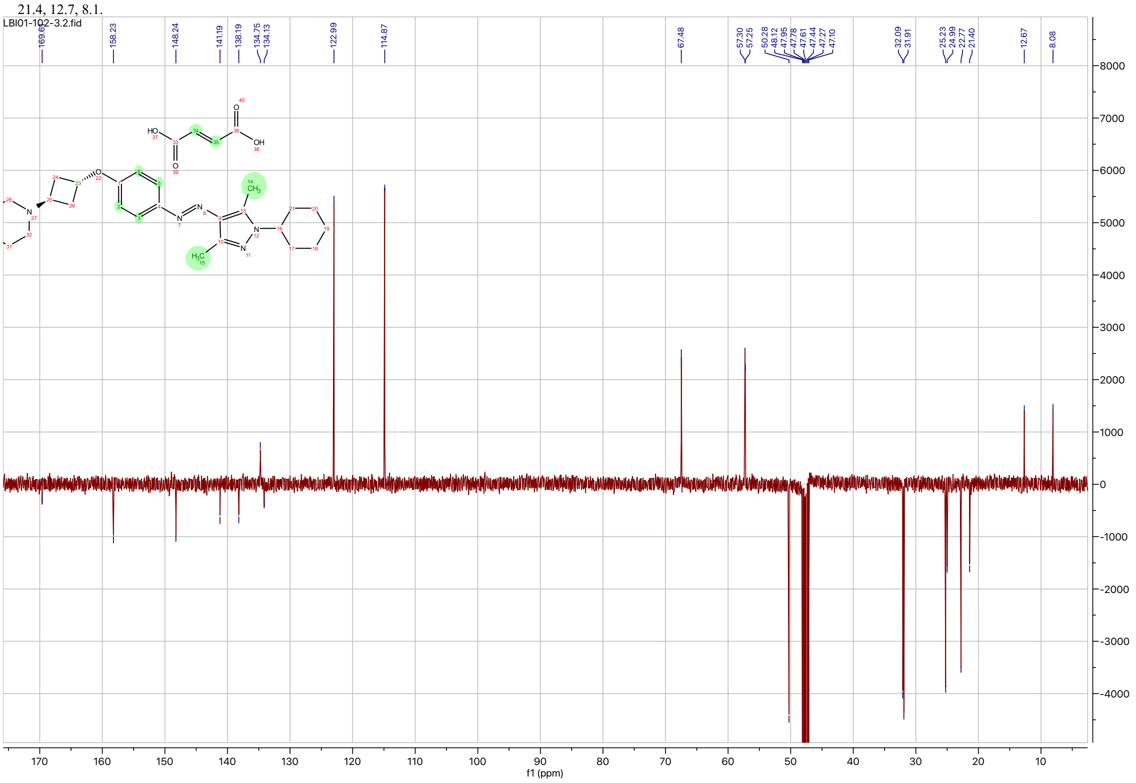

LCMS chromatogram of **3b**

Acquired by : Admin
Date Acquired : 10/5/2022 11:06:49 AM
Sample Name : LBI01-102-3
Sample ID :
Tray# : 1
Vial# : 11
Injection Volume : 1
Data File : C:\LabSolutions\Data\2022\2022-wk40\LBI01-102-3.lcd
Background File : azoblanco05102022 .lcd
Method File : Method SCAN ACID standard azo.lcm
Report Format : DefaultLCMS.lcr
Tuning File : C:\LabSolutions\Tuning File\Tuning-ESI-pos-neg01072015.lct
Processed by : Admin
Modified Date : 10/5/2022 11:31:35 AM

Chromatogram
LBI01-102-3 C:\LabSolutions\Data\2022\2022-wk40\LBI01-102-3.lcd

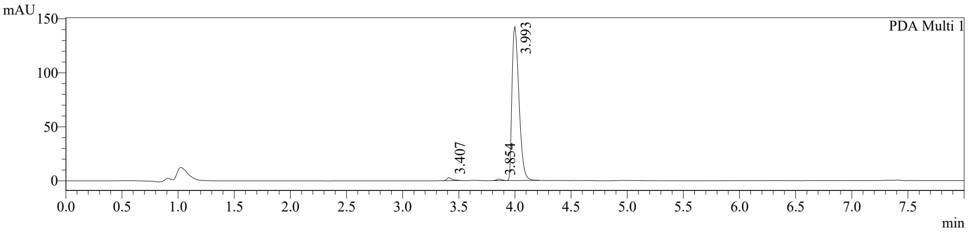

1 PDA Multi 1 / 254nm 4nm

PeakTable

PDA Ch1 254nm 4nm

Peak#	Ret. Time	Area	Height	Area %
1	3.407	9135	2797	1.453
2	3.854	4476	1411	0.712
3	3.993	615139	142722	97.835
Total		628750	146929	100.000

MS Chromatogram
LBI01-102-3 C:\LabSolutions\Data\2022\2022-wk40\LBI01-102-3.lcd

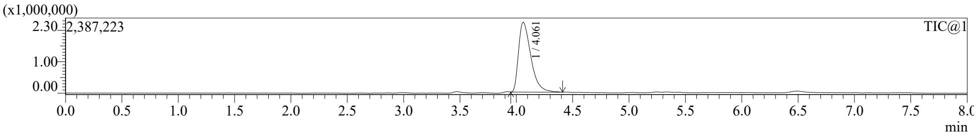

MS Spectrum Graph

#:1 Ret.Time:Averaged 4.050-4.070(Scan#:406-408)
BG Mode:Calc 3.950<->4.410(396<->442)
Mass Peaks:10 Base Peak:436.35(1121215) Polarity:Pos Segment1 - Event1

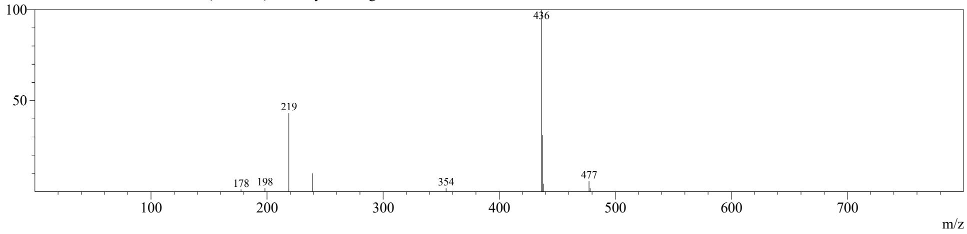

MS Spectrum Table

#:1 Ret.Time:
BG Mode:Calc 3.950<->4.410(396<->442)
Mass Peaks:10 Base Peak:436.35(1121215) Polarity:Pos Segment1 - Event1

#	m/z	Abs.Inten.	Rel.Inten.	Charge	Polarity	Monoisotopic
1	177.60	13483	1.20			
2	198.20	21926	1.96			
3	218.75	484441	43.21			
4	239.25	112443	10.03			
5	354.25	21342	1.90			
6	436.35	1121215	100.00			
7	437.35	348376	31.07			
8	438.35	48937	4.36			
9	477.40	64196	5.73			
10	478.35	21179	1.89			

HRMS spectrum of **3b**

HRMS MedChem

Analysis Info

Analysis Name	D:\Data\ServiceMS\Hans\2023-wk15\VUF26025_4-11-2023_09-46-05_100-1200mzrange.d	Acquisition Date	4/11/2023 9:46:47 AM
Method	100-1200mz range.m	Operator	Demo User
Sample Name	VUF26025	Instrument	impact II
Comment			

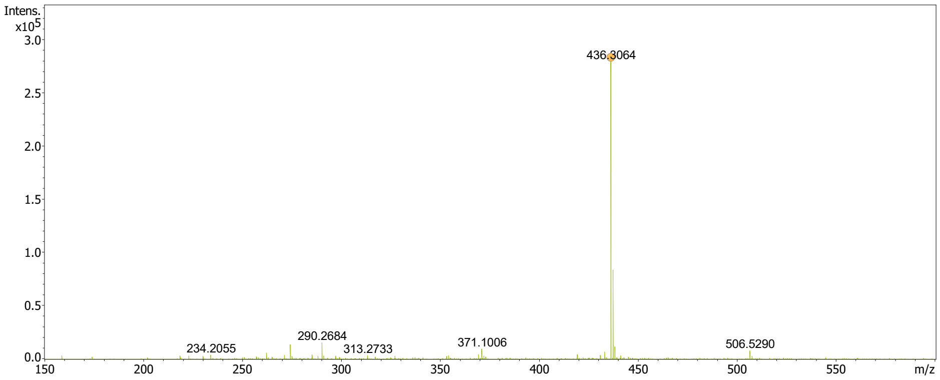

Meas. m/z	#	Ion Formula	m/z	err [ppm]	mSigma	#mSigma	Score	rdb	e⁻ Conf	N-Rule
436.3064	1	C26H38N5O	436.3071	1.6	3.6	1	100.00	11.0	even	ok

^{1}H NMR spectrum of **3c**

^{1}H NMR (500 MHz, MeOD) δ 7.65 (d, J = 9.0 Hz, 2H), 6.83 (d, J = 9.0 Hz, 2H), 6.61 (s, 2H), 4.84 (tt, J = 7.0, 2.1 Hz, 1H), 4.35 (tt, J = 11.6, 4.1 Hz, 1H), 3.98 (dd, J = 11.4, 4.7 Hz, 2H), 3.88 – 3.79 (m, 1H), 3.51 (ddd, J = 12.1, 12.0, 2.0 Hz, 2H), 3.18 – 2.79 (m, 4H), 2.76 – 2.68 (m, 2H), 2.57 – 2.45 (m, 5H), 2.35 (s, 3H), 2.20 – 2.08 (m, 2H), 1.85 – 1.70 (m, 6H), 1.65 – 1.48 (m, 2H).

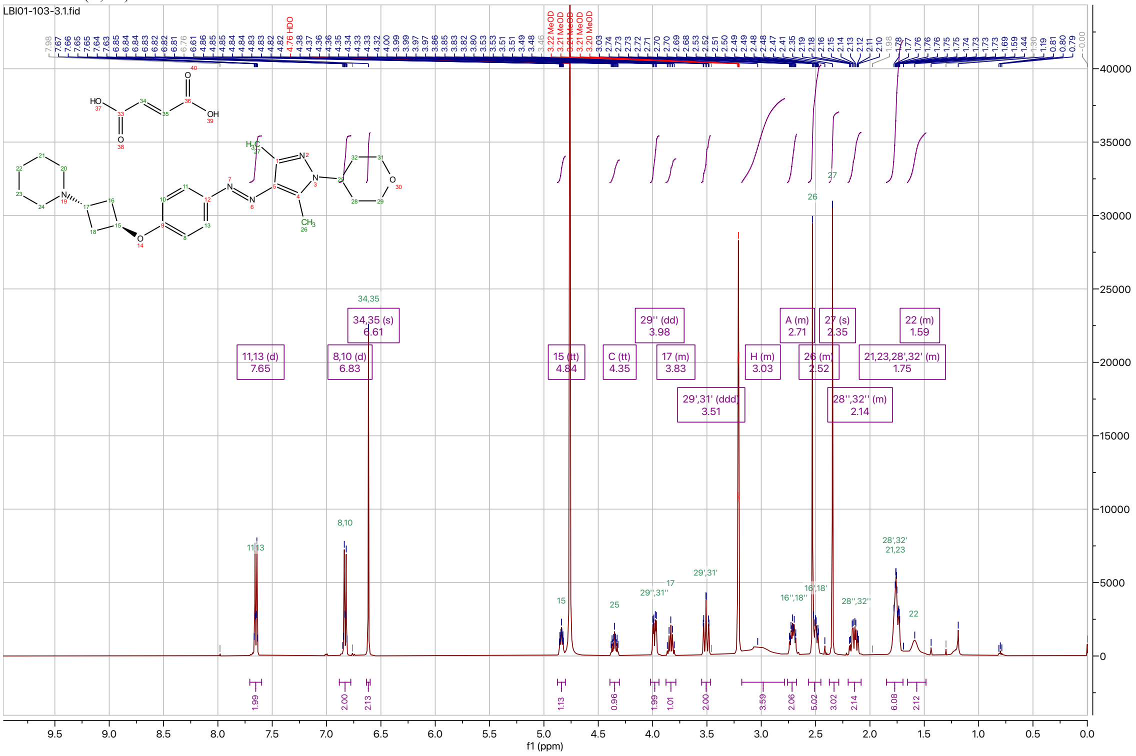

^{13}C NMR spectrum of **3c**

^{13}C NMR (126 MHz, MeOD) δ 169.3, 158.3, 148.2, 141.5, 138.5, 134.7, 134.3, 123.0, 114.9, 67.5, 66.6, 57.3, 54.2, 50.3, 32.0, 31.9, 22.7, 21.4, 12.7, 8.0.

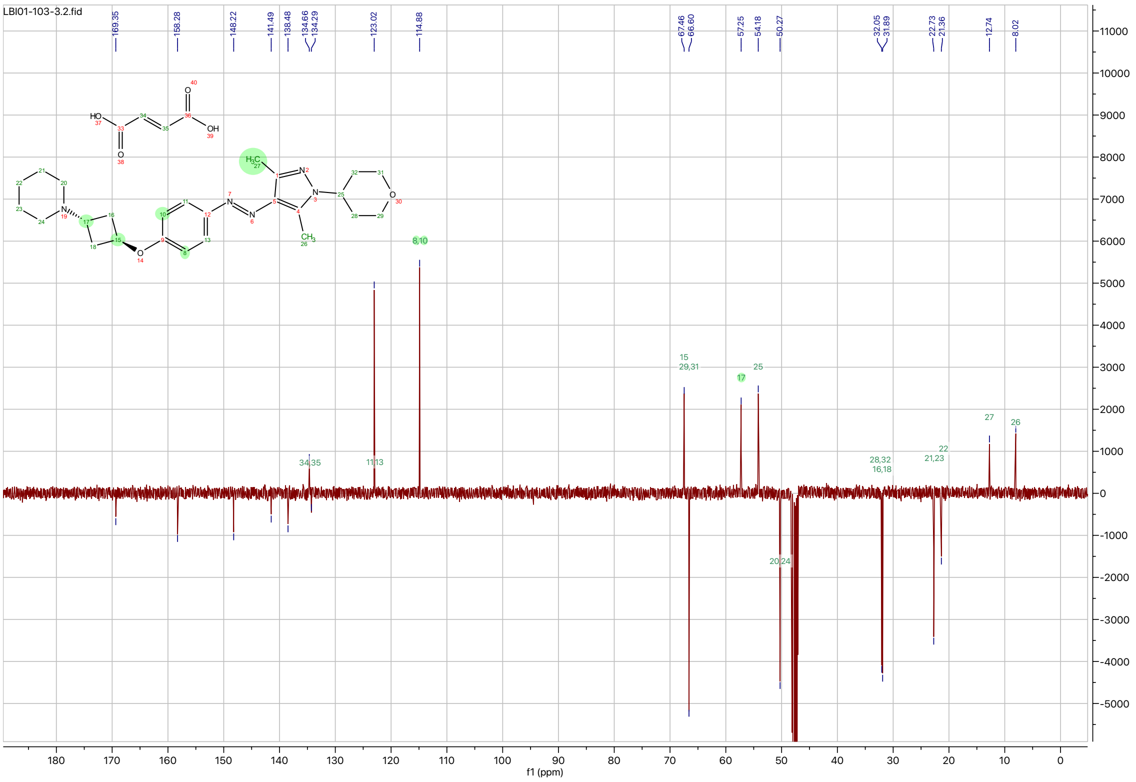

LCMS chromatogram of **3c**

Acquired by : Admin
Date Acquired : 10/5/2022 11:15:28 AM
Sample Name : LBI01-103-3
Sample ID :
Tray# : 1
Vial# : 12
Injection Volume : 1
Data File : C:\LabSolutions\Data\2022\2022-wk40\LBI01-103-3.lcd
Background File : azoblanco05102022 .lcd
Method File : Method SCAN ACID standard azo.lcm
Report Format : DefaultLCMS.lcr
Tuning File : C:\LabSolutions\Tuning File\Tuning-ESI-pos-neg01072015.lct
Processed by : Admin
Modified Date : 10/5/2022 11:33:04 AM

Chromatogram
LBI01-103-3 C:\LabSolutions\Data\2022\2022-wk40\LBI01-103-3.lcd

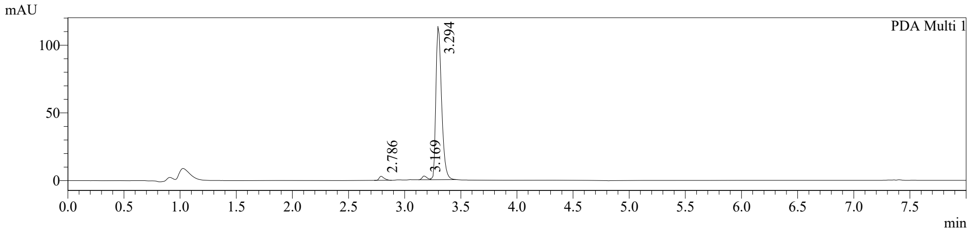

1 PDA Multi 1 / 254nm 4nm

PeakTable

PDA Ch1 254nm 4nm

Peak#	Ret. Time	Area	Height	Area %
1	2.786	9003	2966	2.153
2	3.169	8635	2691	2.065
3	3.294	400505	113163	95.782
Total		418144	118820	100.000

MS Chromatogram
LBI01-103-3 C:\LabSolutions\Data\2022\2022-wk40\LBI01-103-3.lcd

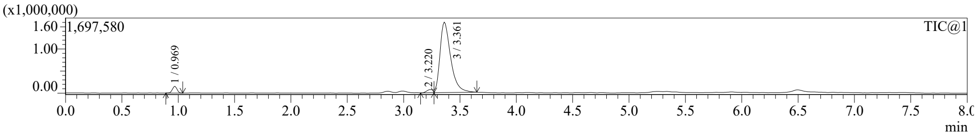

MS Spectrum Graph

#:1 Ret.Time:Averaged 3.350-3.370(Scan#:336-338)
BG Mode:Calc 3.270<->3.650(328<->366)
Mass Peaks:7 Base Peak:438.35(1101191) Polarity:Pos Segment1 - Event1

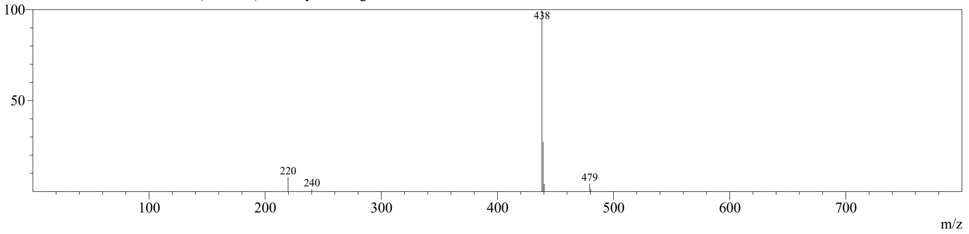

MS Spectrum Table

#:1 Ret.Time:
BG Mode:Calc 3.270<->3.650(328<->366)
Mass Peaks:7 Base Peak:438.35(1101191) Polarity:Pos Segment1 - Event1

#	m/z	Abs.Inten.	Rel.Inten.	Charge	Polarity	Monoisotopic
1	219.70	88521	8.04			
2	240.25	15659	1.42			
3	438.35	1101191	100.00			
4	439.35	302258	27.45			
5	440.35	47139	4.28			
6	479.35	49336	4.48			
7	480.40	16827	1.53			

HRMS spectrum of **3c**

HRMS MedChem

Analysis Info

Analysis Name	D:\Data\ServiceMS\Hans\2023-wk15\VUF26026_4-11-2023_09-51-50_100-1200mzrange.d	Acquisition Date	4/11/2023 9:52:36 AM
Method	100-1200mz range.m	Operator	Demo User
Sample Name	VUF26026	Instrument	impact II
Comment			

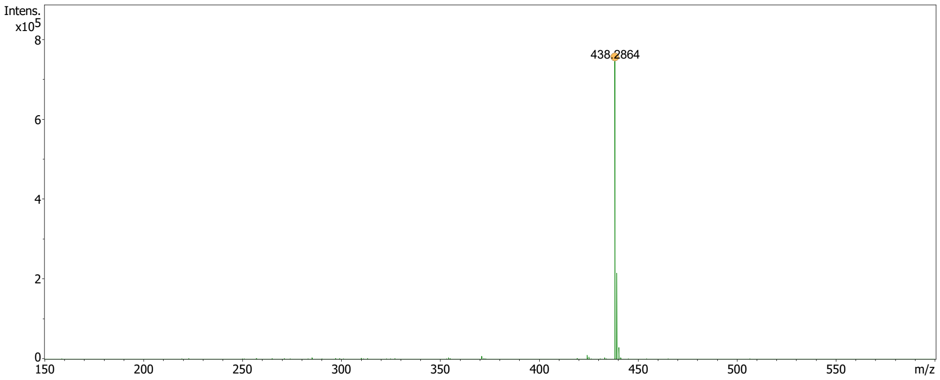

Meas. m/z	#	Ion Formula	m/z	err [ppm]	mSigma	#mSigma	Score	rdb	e⁻ Conf	N-Rule
438.2864	1	C25H36N5O2	438.2864	-0.2	4.0	1	100.00	11.0	even	ok

^{1}H NMR spectrum of **3d**

^{1}H NMR (600 MHz, MeOD) δ 7.72 (d, *J* = 8.9 Hz, 2H), 6.88 (d, *J* = 8.9 Hz, 2H), 4.84 – 4.81 (m, 1H), 4.38 (tt, *J* = 11.6, 4.1 Hz, 1H), 4.23 (d, *J* = 13.5 Hz, 2H), 3.20 – 3.09 (m, 1H), 3.04 – 2.82 (m, 2H), 2.61 (s, 3H), 2.58 – 2.19 (m, 11H), 2.04 (ddd, *J* = 12.6, 12.4, 4.3 Hz, 2H), 1.95 – 1.83 (m, 2H), 1.72 – 1.61 (m, 4H), 1.60 – 1.42 (m, 11H).

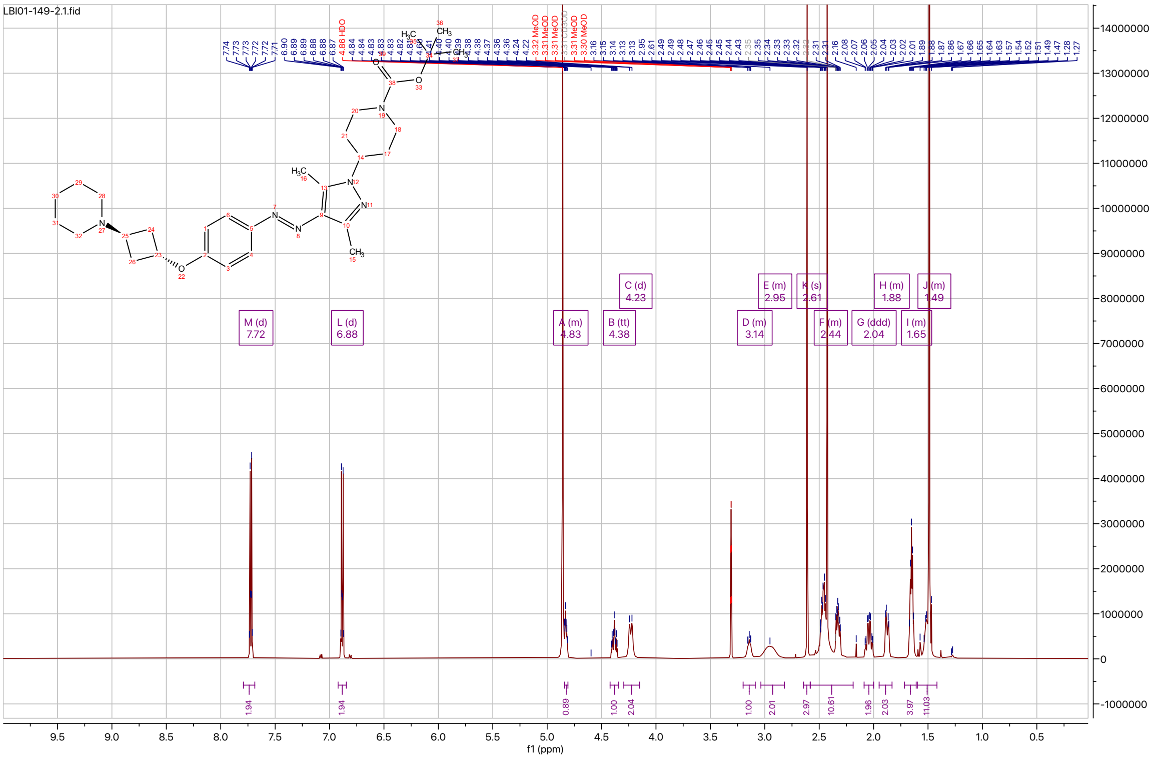

^{13}C NMR spectrum of **3d**

^{13}C NMR (151 MHz, MeOD) δ 160.3, 156.3, 149.2, 142.9, 139.8, 135.6, 135.6, 124.4, 116.2, 81.3, 70.3, 58.7, 56.4, 51.9, 34.2, 32.5, 28.7, 25.9, 24.8, 14.1, 9.4.

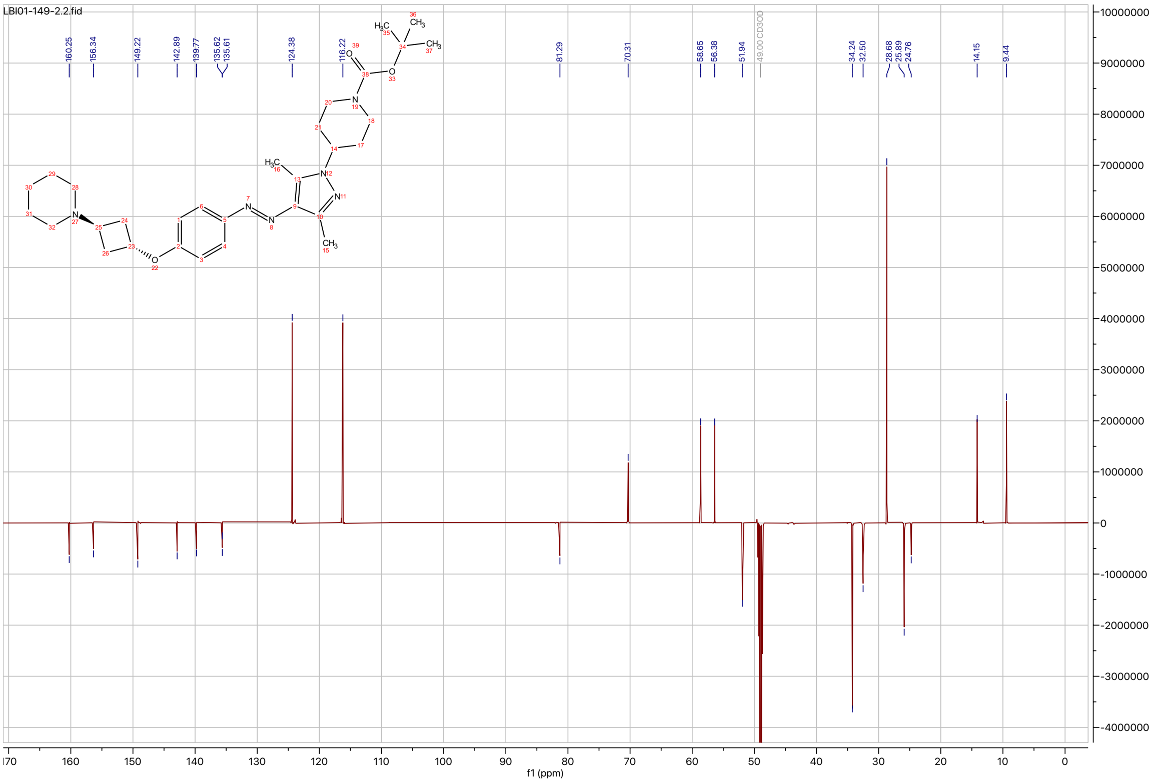

LCMS chromatogram of **3d**

Acquired by	: Admin
Date Acquired	: 11/1/2024 11:59:51 AM
Sample Name	: LBI01-149-2
Sample ID	:
Tray#	: 1
Vial#	: 5
Injection Volume	: 2
Data File	: C:\LabSolutions\Data\2024\2024-wk44\LBI01-149-2.lcd
Background File	: azoblanco 01112024.lcd
Method File	: Method SCAN ACID standard azo.lcm
Report Format	: DefaultLCMS.lcr
Tuning File	: C:\LabSolutions\Tuning File\Tuning-ESI-pos-neg01072015.lct
Processed by	: Admin
Modified Date	: 11/1/2024 12:12:39 PM

Chromatogram
LBI01-149-2 C:\LabSolutions\Data\2024\2024-wk44\LBI01-149-2.lcd

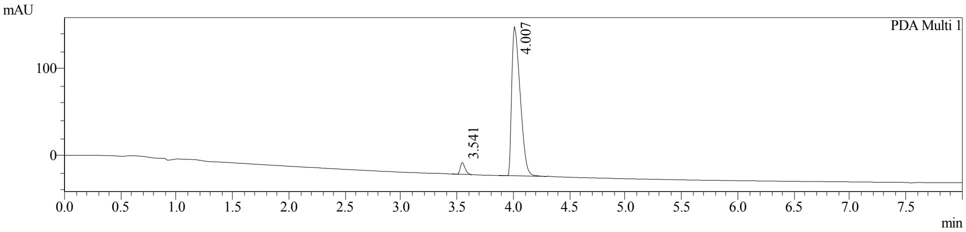

1 PDA Multi 1 / 254nm 4nm

PeakTable

PDA Ch1 254nm 4nm

Peak#	Ret. Time	Area	Height	Name	Area %
1	3.541	39531	13303		4.169
2	4.007	908664	172018		95.831
Total		948195	185321		100.000

MS Chromatogram
LBI01-149-2 C:\LabSolutions\Data\2024\2024-wk44\LBI01-149-2.lcd

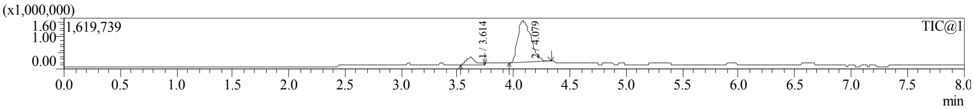

MS Spectrum Graph

#:1 Ret.Time:Averaged 4.070-4.090(Scan#:408-410)
BG Mode:Calc 3.960<->4.330(397<->434)
Mass Peaks:10 Base Peak:537.40(774965) Polarity:Pos Segment1 - Event1

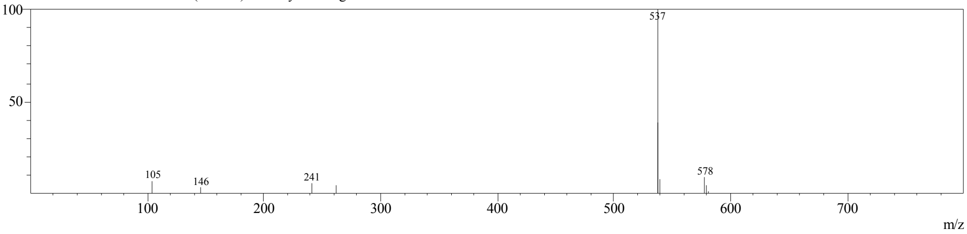

#:2 Ret.Time:Averaged 3.600-3.620(Scan#:361-363)
BG Mode:Calc 3.530<->3.740(354<->375)
Mass Peaks:24 Base Peak:537.45(104973) Polarity:Pos Segment1 - Event1

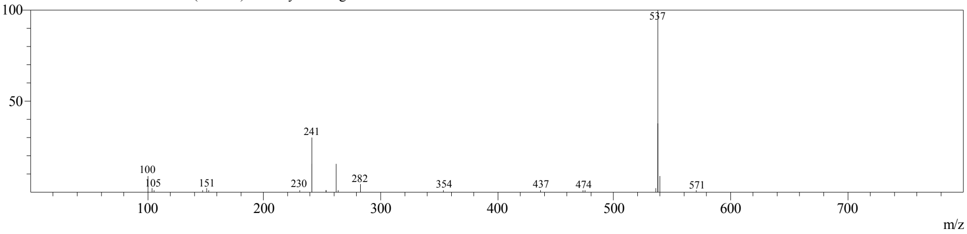

MS Spectrum Table

^{1}H NMR spectrum of **3e**

^{1}H NMR (600 MHz, MeOD) δ 7.73 (d, *J* = 9.2 Hz, 2H), 6.89 (d, *J* = 8.9 Hz, 2H), 4.83 (tt, *J* = 4.1, 2.7 Hz, 2H), 4.34 (tt, *J* = 11.7, 4.0 Hz, 1H), 3.26 – 3.17 (m, 2H), 3.04 (tt, *J* = 7.5, 7.5 Hz, 1H), 2.80 (dd△, *J* = 13.0, 2.6 Hz, 2H), 2.62 (s, 3H), 2.48 – 2.40 (m, 6H), 2.53 – 2.22 (m, 11H), 2.27 (d, *J* = 0.8 Hz, 3H), 2.10 (ddd, *J* = 12.7, 12.6, 4.2 Hz, 2H), 1.95 – 1.88 (m, 2H), 1.64 (tt, *J* = 5.7, 5.7 Hz, 4H), 1.56 – 1.44 (m, 2H).

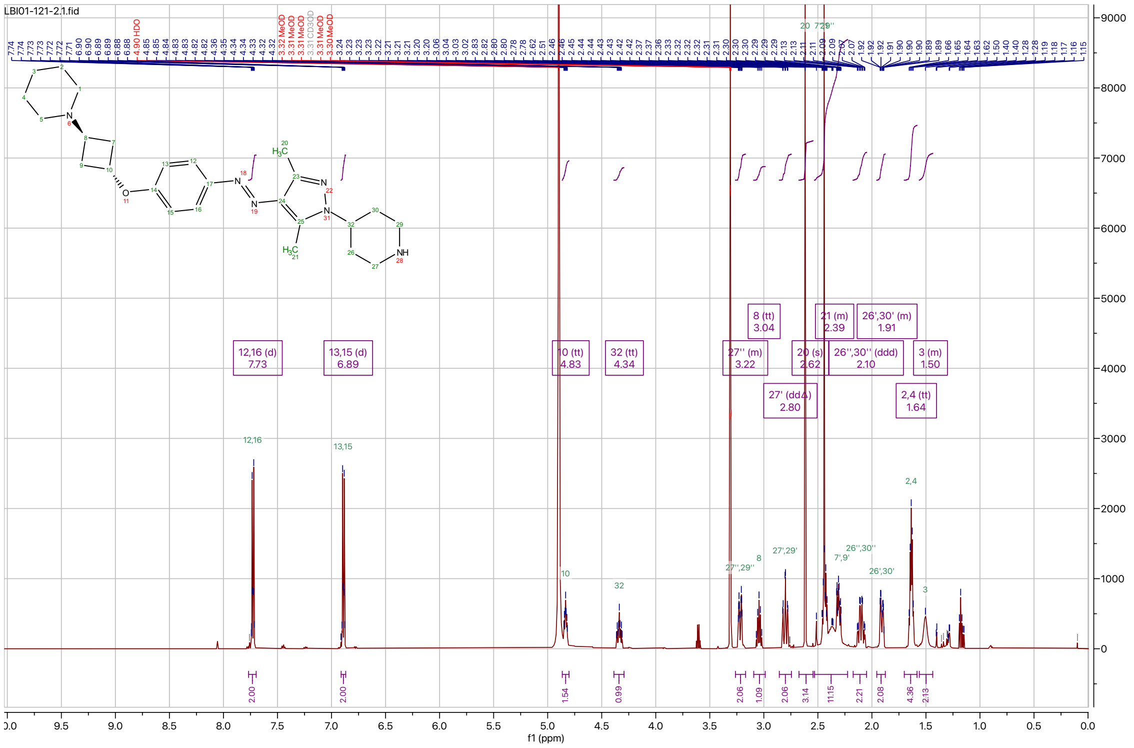

^{13}C NMR spectrum of **3e**

^{13}C NMR (151 MHz, MeOD) δ 160.3, 149.2, 142.7, 139.7, 135.6, 124.3, 116.2, 70.5, 58.7, 56.5, 52.0, 46.0, 34.4, 33.2, 26.1, 25.1, 14.2, 9.4.

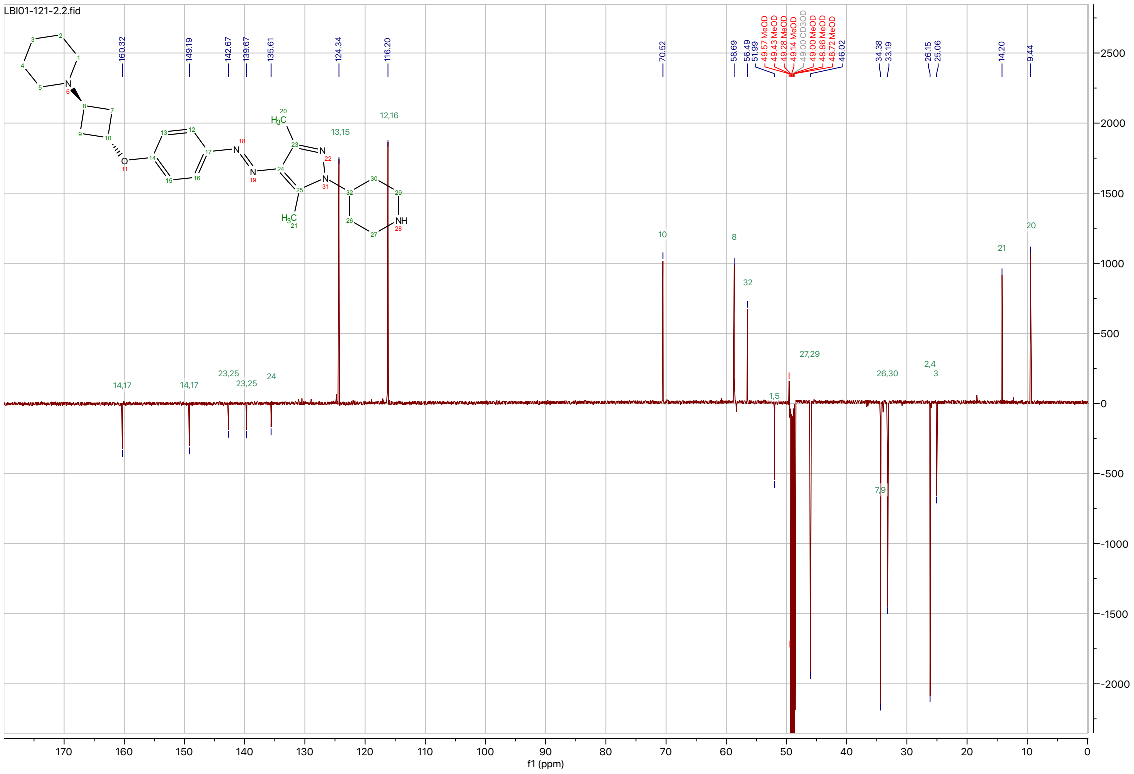

LCMS chromatogram of **3e**

Acquired by	: Admin
Date Acquired	: 2/28/2023 4:19:15 PM
Sample Name	: LBI01-121-3
Sample ID	:
Tray#	: 1
Vial#	: 22
Injection Volume	: 3
Data File	: C:\LabSolutions\Data\2023\2023-wk09\LBI01-121-3.lcd
Background File	: azoblanco 28022024.lcd
Method File	: Method SCAN ACID standard azo.lcm
Report Format	: DefaultLCMS.lcr
Tuning File	: C:\LabSolutions\Tuning File\Tuning-ESI-pos-neg01072015.lct
Processed by	: Admin
Modified Date	: 2/28/2023 4:28:54 PM

Chromatogram
LBI01-121-3 C:\LabSolutions\Data\2023\2023-wk09\LBI01-121-3.lcd

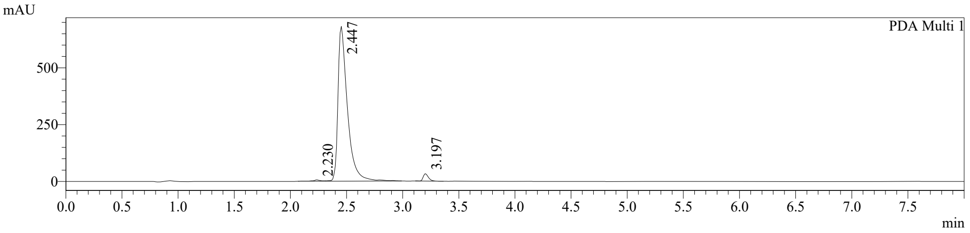

1 PDA Multi 1 / 254nm 4nm

PeakTable

PDA Ch1 254nm 4nm

Peak#	Ret. Time	Area	Height	Name	Area %
1	2.230	25927	5712		0.631
2	2.447	3983832	680577		96.888
3	3.197	102044	32422		2.482
Total		4111803	718710		100.000

MS Chromatogram
LBI01-121-3 C:\LabSolutions\Data\2023\2023-wk09\LBI01-121-3.lcd

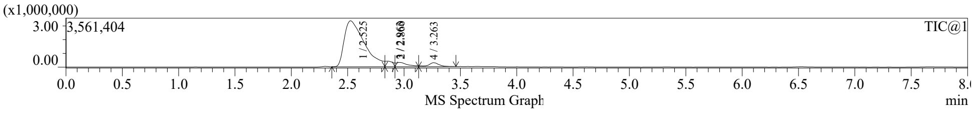

MS Spectrum Graph

#:1 Ret.Time:Averaged 2.510-2.530(Scan#:252-254)
BG Mode:Calc 2.360<->2.830(237<->284)
Mass Peaks:7 Base Peak:219.20(2418377) Polarity:Pos Segment1 - Event1

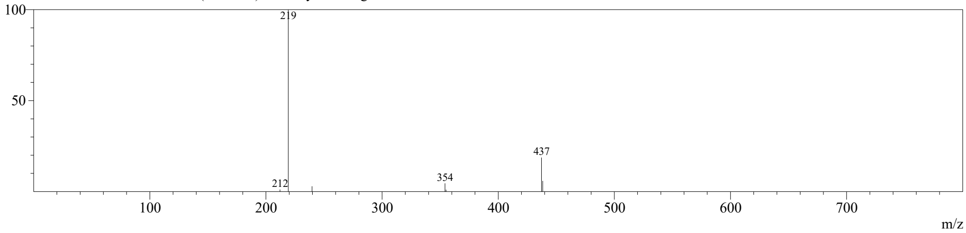

MS Spectrum Table

#:1 Ret.Time:
BG Mode:Calc 2.360<->2.830(237<->284
Mass Peaks:7 Base Peak:219.20(2418377) Polarity:Pos Segment1 - Event

#	m/z	Abs.Inten.	Rel.Inten.	Charge	Polarity	Monoisotopic
1	212.10	28181	1.17			
2	219.20	2418377	100.00			
3	239.70	71895	2.97			
4	354.15	110021	4.55			
5	355.15	25740	1.06			
6	437.30	452176	18.70			
7	438.30	142164	5.88			

HRMS spectrum of **3e**

HRMS MedChem

Analysis Info

Analysis Name	D:\Data\ServiceMS\Hans\2023-wk15\VUF26064_4-11-2023_09-28-42_100-1200mzrange.d	Acquisition Date	4/11/2023 9:29:25 AM
Method	100-1200mz range.m	Operator	Demo User
Sample Name	VUF26064	Instrument	impact II
Comment			

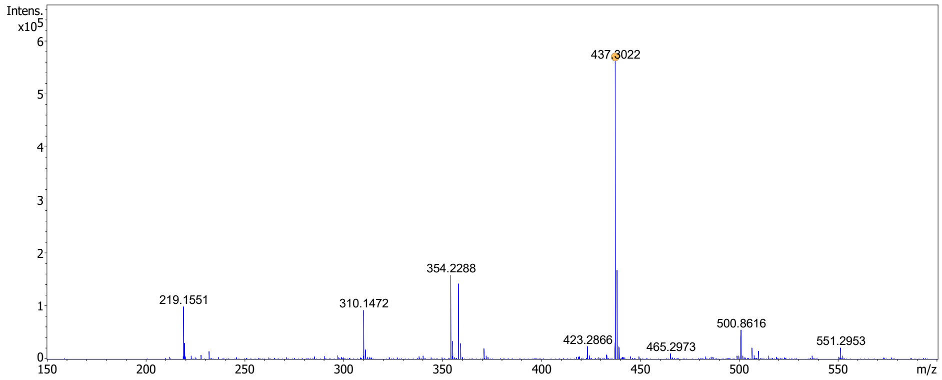

Meas. m/z	#	Ion Formula	m/z	err [ppm]	mSigma	#mSigma	Score	rdb	e⁻ Conf	N-Rule
437.3022	1	C25H37N6O	437.3023	0.2	1.4	1	100.00	11.0	even	ok

Bruker Compass DataAnalysis 6.0 printed: 4/11/2023 9:37:02 AM by: demo Page 1 of 1

^{1}H NMR spectrum of **3f**

^{1}H NMR (600 MHz, MeOD) δ 7.72 (d, J = 8.9 Hz, 2H), 6.89 (d, J = 8.9 Hz, 2H), 4.83 (tt, J = 6.8, 3.4 Hz, 1H), 4.20 (ddd△, J = 11.1, 7.8, 4.2 Hz, 1H), 3.09 – 2.99 (m, 3H), 2.61 (s, 3H), 2.55 – 2.14 (m, 18H), 1.97 – 1.82 (m, 2H), 1.63 (tt, J = 5.7, 5.7 Hz, 4H), 1.57 – 1.40 (m, 2H).

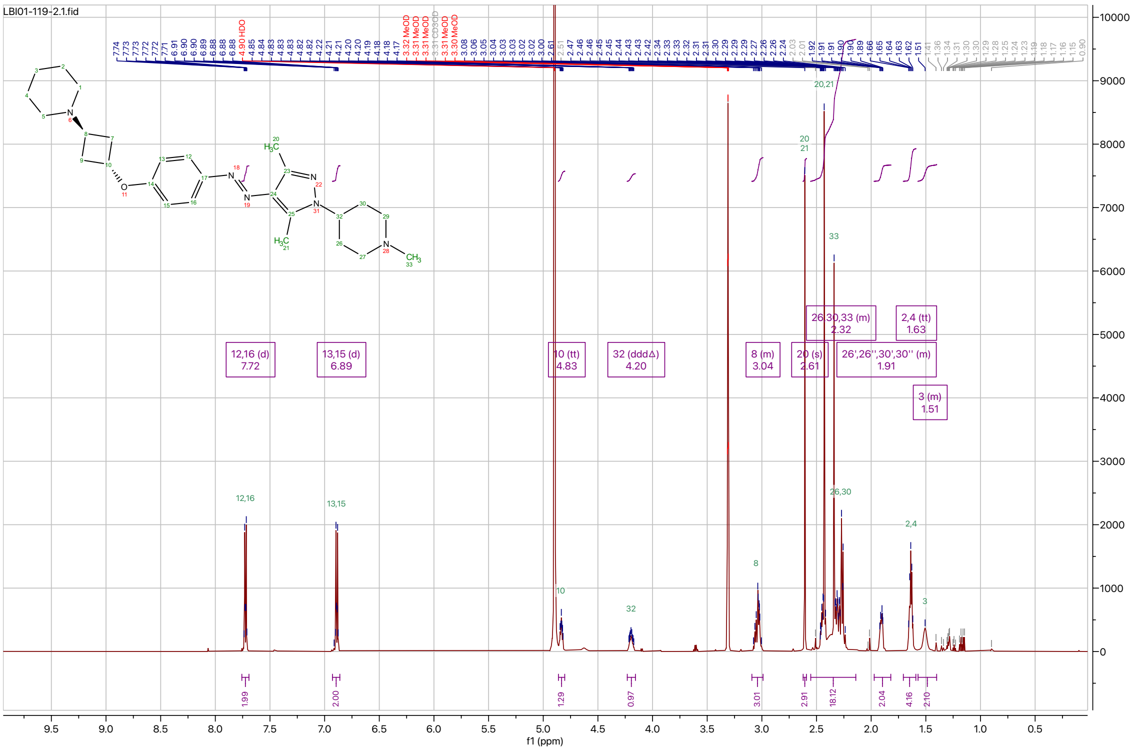

^{13}C NMR spectrum of **3f**

^{13}C NMR (151 MHz, MeOD) δ 160.3, 149.2, 142.6, 139.8, 135.8, 124.3, 116.2, 70.5, 58.7, 56.3, 55.7, 52.0, 46.1, 34.4, 32.2, 26.1, 25.1, 14.2, 9.4.

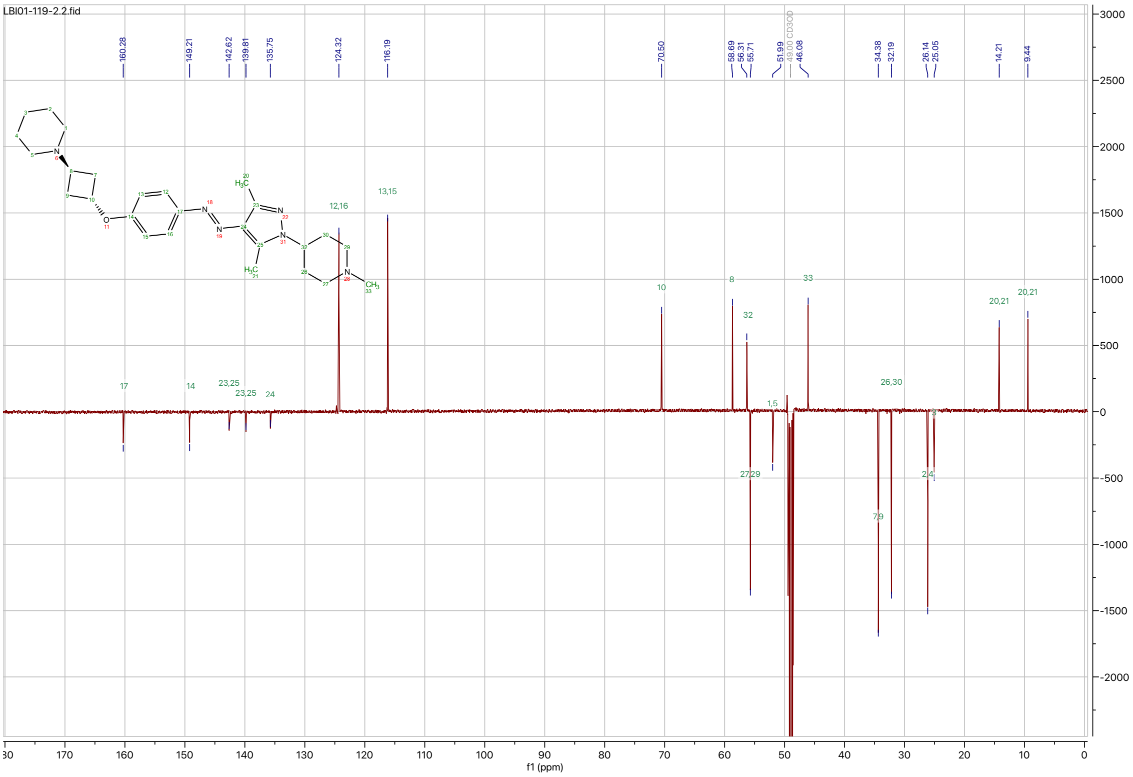

LCMS chromatogram of **3f**

Acquired by	: Admin
Date Acquired	: 2/28/2023 4:10:37 PM
Sample Name	: LBI01-119-3
Sample ID	:
Tray#	: 1
Vial#	: 21
Injection Volume	: 3
Data File	: C:\LabSolutions\Data\2023\2023-wk09\LBI01-119-3.lcd
Background File	: azoblanco 28022024.lcd
Method File	: Method SCAN ACID standard azo.lcm
Report Format	: DefaultLCMS.lcr
Tuning File	: C:\LabSolutions\Tuning File\Tuning-ESI-pos-neg01072015.lct
Processed by	: Admin
Modified Date	: 2/28/2023 4:24:50 PM

Chromatogram
LBI01-119-3 C:\LabSolutions\Data\2023\2023-wk09\LBI01-119-3.lcd

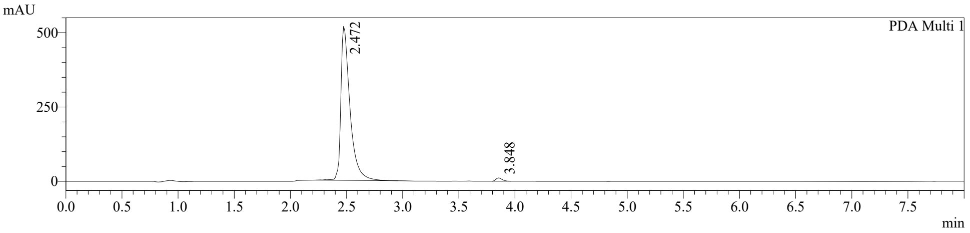

1 PDA Multi 1 / 254nm 4nm

PeakTable

PDA Ch1 254nm 4nm

Peak#	Ret. Time	Area	Height	Name	Area %
1	2.472	2940086	517790		98.584
2	3.848	42215	11002		1.416
Total		2982301	528793		100.000

MS Chromatogram
LBI01-119-3 C:\LabSolutions\Data\2023\2023-wk09\LBI01-119-3.lcd

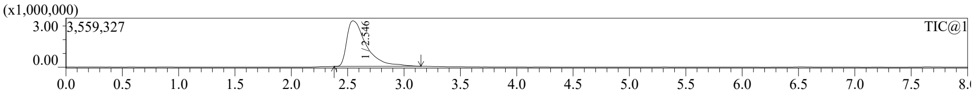

MS Spectrum Graph

#:1 Ret.Time:Averaged 2.540-2.560(Scan#:255-257)
BG Mode:Calc 2.380<->3.150(239<->316)
Mass Peaks:5 Base Peak:226.25(2691780) Polarity:Pos Segment1 - Event1

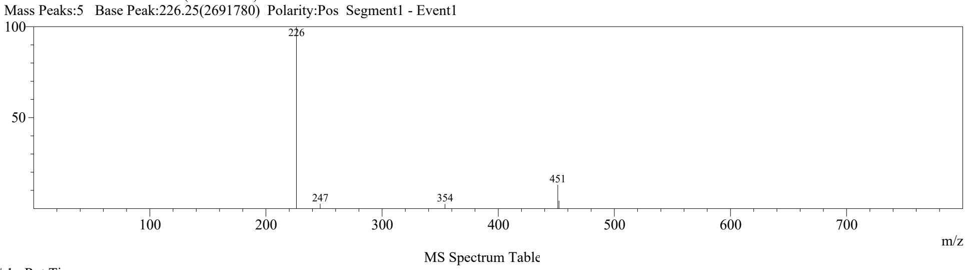

MS Spectrum Table

#:1 Ret.Time:
BG Mode:Calc 2.380<->3.150(239<->316
Mass Peaks:5 Base Peak:226.25(2691780) Polarity:Pos Segment1 - Event

#	m/z	Abs.Inten.	Rel.Inten.	Charge	Polarity	Monoisotopic
1	226.25	2691780	100.00			
2	246.65	71496	2.66			
3	354.15	67945	2.52			
4	451.30	352064	13.08			
5	452.30	116078	4.31			

HRMS spectrum of **3f**

HRMS MedChem

Analysis Info

Analysis Name	D:\Data\ServiceMS\Hans\2023-wk15\VUF26063_4-11-2023_10-26-41_100-1200mzrange.d	Acquisition Date	4/11/2023 10:27:30 AM
Method	100-1200mz range.m	Operator	Demo User
Sample Name	VUF26063	Instrument	impact II
Comment			

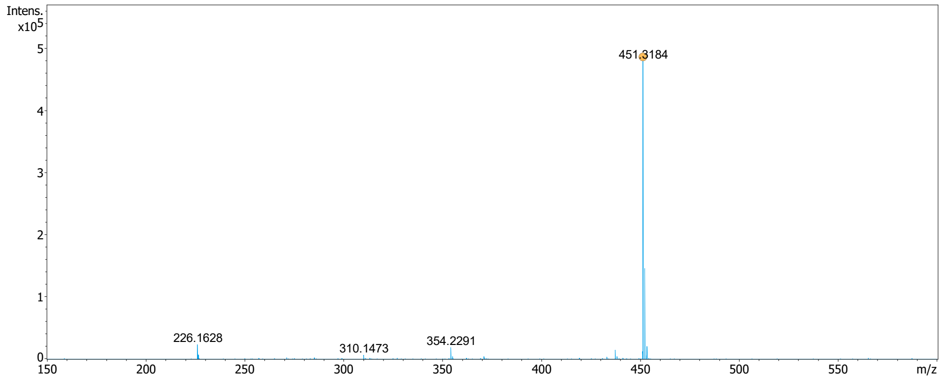

Meas. m/z	#	Ion Formula	m/z	err [ppm]	mSigma	#mSigma	Score	rdb	e⁻ Conf	N-Rule
451.3184	1	C26H39N6O	451.3180	-1.0	3.1	1	100.00	11.0	even	ok

References

(1) Stricker, L.; Fritz, E. C.; Peterlechner, M.; Doltsinis, N. L.; Ravoo, B. J. Arylazopyrazoles as Light-Responsive Molecular Switches in Cyclodextrin-Based Supramolecular Systems. *J Am Chem Soc* **2016**, *138* (13), 4547–4554. https://doi.org/10.1021/jacs.6b00484.

(2) Wijtmans, M.; Denonne, F.; Célanire, S.; Gillard, M.; Hulscher, S.; Delaunoy, C.; Van Houtvin, N.; Bakker, R. A.; Defays, S.; Gérard, J.; Grooters, L.; Hubert, D.; Timmerman, H.; Leurs, R.; Talaga, P.; De Esch, I. J. P.; Provins, L. Histamine H 3 Receptor Ligands with a 3-Cyclobutoxy Motif: A Novel and Versatile Constraint of the Classical 3-Propoxy Linker. *Medchemcomm* **2010**, *1* (1), 39–44. https://doi.org/10.1039/c0md00056f.

(3) Hauwert, N. J.; Mocking, T. A. M.; Da Costa Pereira, D.; Kooistra, A. J.; Wijnen, L. M.; Vreeker, G. C. M.; Verweij, E. W. E.; De Boer, A. H.; Smit, M. J.; De Graaf, C.; Vischer, H. F.; De Esch, I. J. P.; Wijtmans, M.; Leurs, R. Synthesis and Characterization of a Bidirectional Photoswitchable Antagonist Toolbox for Real-Time GPCR Photopharmacology. *J Am Chem Soc* **2018**, *140* (12), 4232–4243. https://doi.org/10.1021/jacs.7b11422.

(4) Peng, X.; Yang, L.; Liu, Z.; Lou, S.; Mei, S.; Li, M.; Chen, Z.; Zhang, H. Structural Basis for Recognition of Antihistamine Drug by Human Histamine Receptor. *Nat Commun* **2022**, *13* (1), 1–9. https://doi.org/10.1038/s41467-022-33880-y.

(5) Mocking, T. A. M.; Verweij, E. W. E.; Vischer, H. F.; Leurs, R. Homogeneous, Real-Time NanoBRET Binding Assays for the Histamine H3 and H4 Receptors on Living Cells. *Mol Pharmacol* **2018**, *94* (6), 1371–1381. https://doi.org/10.1124/mol.118.113373.

(6) Grätz, L.; Tropmann, K.; Bresinsky, M.; Müller, C.; Bernhardt, G.; Pockes, S. NanoBRET Binding Assay for Histamine H2 Receptor Ligands Using Live Recombinant HEK293T Cells. *Sci Rep* **2020**, *10* (1), 1–10. https://doi.org/10.1038/s41598-020-70332-3.

(7) Bosma, R.; Wang, Z.; Kooistra, A. J.; Bushby, N.; Kuhne, S.; Van Den Bor, J.; Waring, M. J.; De Graaf, C.; De Esch, I. J.; Vischer, H. F.; Sheppard, R. J.; Wijtmans, M.; Leurs, R. Route to Prolonged Residence Time at the Histamine H1 Receptor: Growing from Desloratadine to Rupatadine. *J Med Chem* **2019**, *62* (14), 6630–6644. https://doi.org/10.1021/acs.jmedchem.9b00447.

(8) Dixon, A. S.; Schwinn, M. K.; Hall, M. P.; Zimmerman, K.; Otto, P.; Lubben, T. H.; Butler, B. L.; Binkowski, B. F.; MacHleidt, T.; Kirkland, T. A.; Wood, M. G.; Eggers, C. T.; Encell, L. P.; Wood, K. V. NanoLuc Complementation Reporter Optimized for Accurate Measurement of Protein Interactions in Cells. *ACS Chem Biol* **2016**, *11* (2), 400–408. https://doi.org/10.1021/acschembio.5b00753.

(9) Yung-Chi, C.; Prusoff, W. H. Relationship between the Inhibition Constant (KI) and the Concentration of Inhibitor Which Causes 50 per Cent Inhibition (I50) of an Enzymatic Reaction. *Biochem Pharmacol* **1973**, *22* (23), 3099–3108. https://doi.org/10.1016/0006-2952(73)90196-2.

(10) Webb, B.; Sali, A. Comparative Protein Structure Modeling Using MODELLER. *Curr Protoc Bioinformatics* **2016**, *54* (1), 5.6.1-5.6.37. https://doi.org/10.1002/cpbi.3.

(11) Shen, Q.; Tang, X.; Wen, X.; Cheng, S.; Xiao, P.; Zang, S. K.; Shen, D. D.; Jiang, L.; Zheng, Y.; Zhang, H.; Xu, H.; Mao, C.; Zhang, M.; Hu, W.; Sun, J. P.; Zhang, Y.; Chen,

Z. Molecular Determinant Underlying Selective Coupling of Primary G-Protein by Class A GPCRs. *Advanced Science* **2024**, *11* (23), 2310120. https://doi.org/10.1002/advs.202310120.
(12) Miller-Gallacher, J. L.; Nehmé, R.; Warne, T.; Edwards, P. C.; Schertler, G. F. X.; Leslie, A. G. W.; Tate, C. G. The 2.1 Å Resolution Structure of Cyanopindolol-Bound B1-Adrenoceptor Identifies an Intramembrane Na+ Ion That Stabilises the Ligand-Free Receptor. *PLoS One* **2014**, *9* (3), e92727. https://doi.org/10.1371/journal.pone.0092727.
(13) Abagyan, R.; Totrov, M.; Kuznetsov, D. ICM—A New Method for Protein Modeling and Design: Applications to Docking and Structure Prediction from the Distorted Native Conformation. *J Comput Chem* **1994**, *15* (5), 488–506. https://doi.org/10.1002/jcc.540150503.
(14) Lee, J.; Cheng, X.; Swails, J. M.; Yeom, M. S.; Eastman, P. K.; Lemkul, J. A.; Wei, S.; Buckner, J.; Jeong, J. C.; Qi, Y.; Jo, S.; Pande, V. S.; Case, D. A.; Brooks, C. L.; MacKerell, A. D.; Klauda, J. B.; Im, W. CHARMM-GUI Input Generator for NAMD, GROMACS, AMBER, OpenMM, and CHARMM/OpenMM Simulations Using the CHARMM36 Additive Force Field. *J Chem Theory Comput* **2016**, *12* (1), 405–413. https://doi.org/10.1021/acs.jctc.5b00935.
(15) Vanommeslaeghe, K.; Hatcher, E.; Acharya, C.; Kundu, S.; Zhong, S.; Shim, J.; Darian, E.; Guvench, O.; Lopes, P.; Vorobyov, I.; Mackerell, A. D. CHARMM General Force Field: A Force Field for Drug-like Molecules Compatible with the CHARMM All-Atom Additive Biological Force Fields. *J Comput Chem* **2010**, *31* (4), 671–690. https://doi.org/10.1002/jcc.21367.
(16) Klaja, O.; Frank, J. A.; Trauner, D.; Bondar, A. N. Potential Energy Function for a Photo-Switchable Lipid Molecule. *J Comput Chem* **2020**, *41* (27), 2336–2351. https://doi.org/10.1002/jcc.26387.
(17) Huang, J.; Rauscher, S.; Nawrocki, G.; Ran, T.; Feig, M.; De Groot, B. L.; Grubmüller, H.; MacKerell, A. D. CHARMM36m: An Improved Force Field for Folded and Intrinsically Disordered Proteins. *Nat Methods* **2016**, *14* (1), 71–73. https://doi.org/10.1038/nmeth.4067.
(18) Páll, S.; Zhmurov, A.; Bauer, P.; Abraham, M.; Lundborg, M.; Gray, A.; Hess, B.; Lindahl, E. Heterogeneous Parallelization and Acceleration of Molecular Dynamics Simulations in GROMACS. *Journal of Chemical Physics* **2020**, *153* (13), 134110. https://doi.org/10.1063/5.0018516.
(19) Bouysset, C.; Fiorucci, S. ProLIF: A Library to Encode Molecular Interactions as Fingerprints. *J Cheminform* **2021**, *13* (1), 1–9. https://doi.org/https://doi.org/10.1186/s13321-021-00548-6.
(20) Ahmed, Z.; Siiskonen, A.; Virkki, M.; Priimagi, A. Controlling Azobenzene Photoswitching through Combined Ortho-Fluorination and -Amination. *Chem Commun* **2017**, *53* (93), 12520–12523. https://doi.org/10.1039/c7cc07308a.
